# Supplementary figures and images for: Taxonomic, molecular and ecological approach reveals high diversity of vector sand flies, varied blood source supply and a high detection rate of Leishmania DNA in Colombian Amazon region
Source: PLoS Negl Trop Dis. 2025 Sep 5;19(9):e0013445. doi: 10.1371/journal.pntd.0013445 (PMC12412933; doi:10.1371/journal.pntd.0013445)

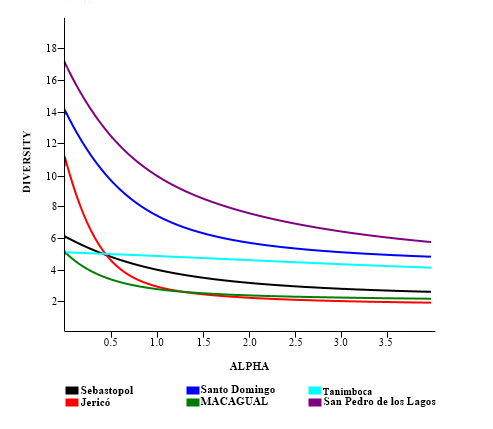

Supplement: S1 Fig — (TIF) [file pntd.0013445.s001.tif]

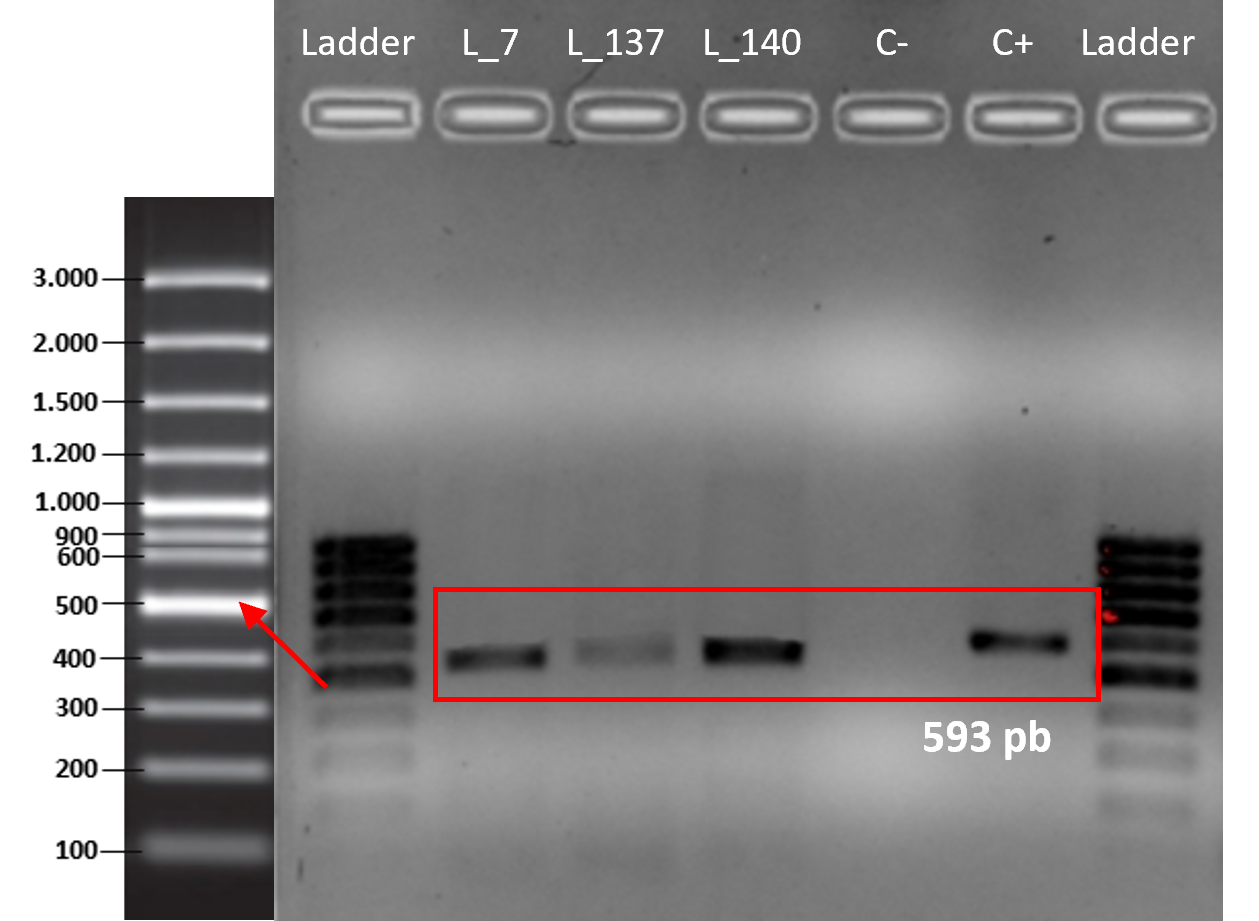

Supplement: S2 Fig — (TIF) [file pntd.0013445.s002.tif]

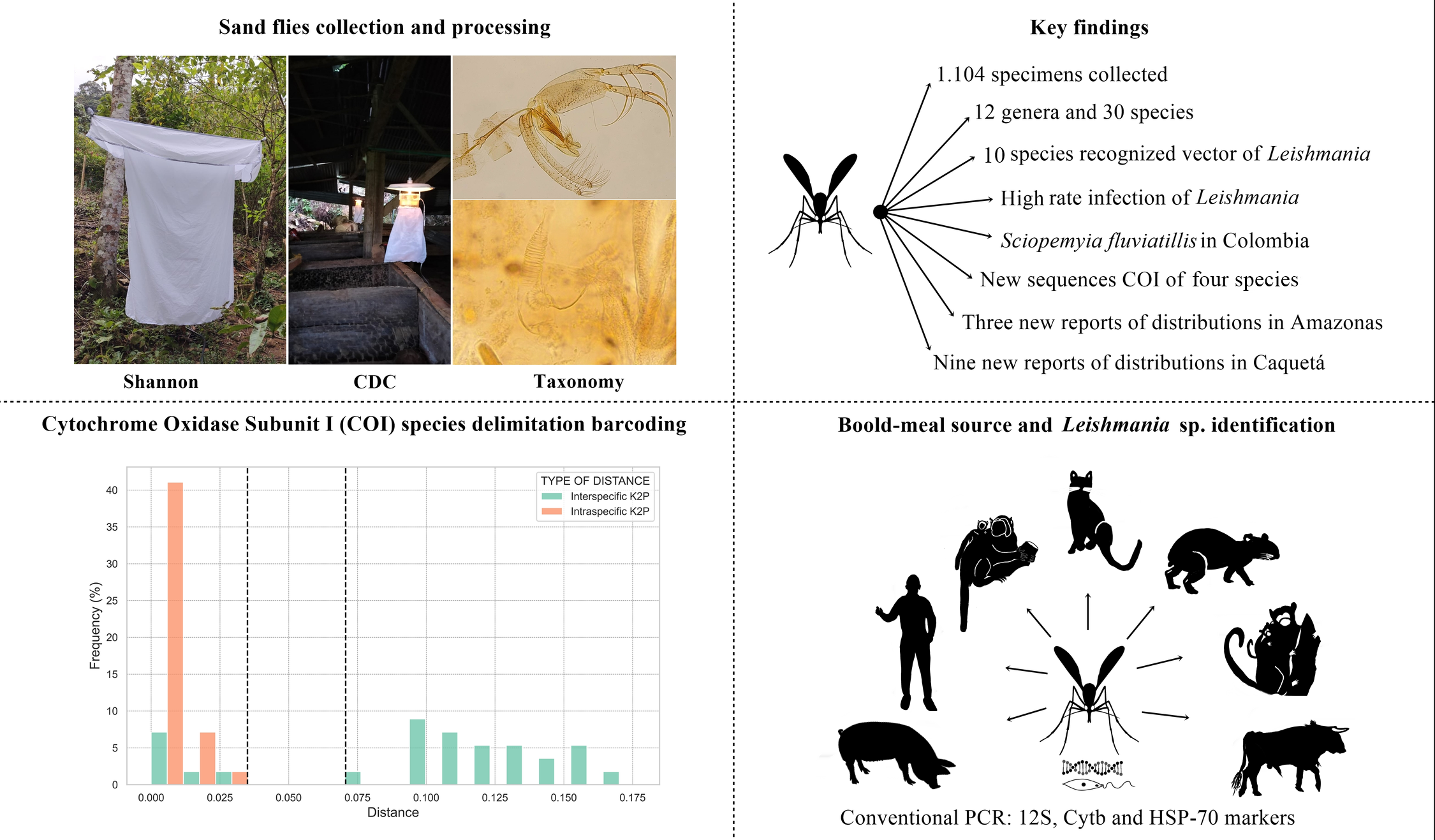

Supplement: S3 Fig — (TIF) [file pntd.0013445.s003.tif]
