# Supplementary material for: Taxonomic, molecular and ecological approach reveals high diversity of vector sand flies, varied blood source supply and a high detection rate of Leishmania DNA in Colombian Amazon region
Source: PLoS Negl Trop Dis. 2025 Sep 5;19(9):e0013445. doi: 10.1371/journal.pntd.0013445 (PMC12412933; doi:10.1371/journal.pntd.0013445)
Supplement: S2 Table — (DOCX) [file pntd.0013445.s005.docx]

| **Species** | **Analyzed sequences** | **Number of haplotypes** | **Number of Polymorphic Sites** | **Haplotype diversity** | **Nucleotide diversity** | | **Intraspecific diversity** | | **Nearest neighbor (NN) species (%)** | | | **% BLAST** | **GenBank Code** |
| --- | --- | --- | --- | --- | --- | --- | --- | --- | --- | --- | --- | --- | --- |
|  |  |  |  |  | ***Pi*** | ***Pi (JC)*** | ***P*** | **K2P** | **Species** | ***P*** | **K2P** |  |  |
| *Br*. *mesai* | 1 | 1 | 0 | 0.000 | 0.000 | 0.000 | 0.000 | 0.000 | *Lu*. (*Trl*.) *sherlocki* | 13.9 | 15.6 | 98.8 | [OP964209.1](https://www.ncbi.nlm.nih.gov/nucleotide/OP964209.1?report=genbank&log$=nuclalign&blast_rank=1&RID=43AS8N4T016) |
| *Ev*. (*Eva*.) *saulensis* | 1 | 1 | 0 | 0.000 | 0.000 | 0.000 | 0.000 | 0.000 | *Ty*. *witoto* | 14.6 | 16.2 | 99.5 | [OP346790.1](https://www.ncbi.nlm.nih.gov/nucleotide/OP346790.1?report=genbank&log$=nucltop&blast_rank=1&RID=4361329X016) |
| *Ev*. (*Eva*.) *georgii* | 2 | 1 | 0 | 0.000 | 0.000 | 0.000 | 0.000 | 0.000 | *Ny*. *antunesi* | 14.5 | 16.2 | 93.8 | [OQ922309.1](https://www.ncbi.nlm.nih.gov/nucleotide/OQ922309.1?report=genbank&log$=nucltop&blast_rank=1&RID=435XKCZU016) |
| *Ev*. (*Ald*.) *walkeri* | 2 | 2 | 8 | 1.000 | 0.013 | 0.013 | 0.013 | 0.013 | *Ny*. *fraihai* | 12.4 | 13.9 | 100 - 99.5 | [OP964213.1](https://www.ncbi.nlm.nih.gov/nucleotide/OP964213.1?report=genbank&log$=nucltop&blast_rank=1&RID=ZG0G3673013) |
| *Lu*. (*Hel*.) *tortura ** | 6 | 4 | 4 | 0.800 | 0.003 | 0.003 | 0.003 | 0.003 | *Vi*. *tuberculata* | 13.2 | 14.7 | 0.0 | N/A |
| *Lu*. (*Trl*.) *sherlocki* | 3 | 3 | 8 | 1.000 | 0.009 | 0.009 | 0.009 | 0.009 | *Ev*. (*Ald*.) *walkeri* | 12.4 | 13.9 | 99.2 | [OP964238.1](https://www.ncbi.nlm.nih.gov/nucleotide/OP964238.1?report=genbank&log$=nuclalign&blast_rank=1&RID=437AJY9E01N) |
| *Mi*. (*Mic*.) *pilosa* | 1 | 1 | 0 | 0.000 | 0.000 | 0.000 | 0.000 | 0.000 | *Vi*. *caprina* | 11.8 | 12.9 | 97.6 | [OR555653.1](https://www.ncbi.nlm.nih.gov/nucleotide/OR555653.1?report=genbank&log$=nucltop&blast_rank=4&RID=437YB5N601N) |
| *Ny*. *antunesi* | 5 | 3 | 3 | 0.700 | 0.002 | 0.002 | 0.002 | 0.002 | *Ny*. *yuilli pajoti* | 1.6 | 1.6 | 100 - 99.5 | [OP964267.1](https://www.ncbi.nlm.nih.gov/nucleotide/OP964267.1?report=genbank&log$=nucltop&blast_rank=1&RID=ZD24G68N01R) |
| *Ny*. *fraihai* | 5 | 3 | 11 | 0.800 | 0.010 | 0.010 | 0.002 | 0.002 | *Ny*. *antunesi* | 2.6 | 2.6 | 99.8 - 99.3 | [OP964268.1](https://www.ncbi.nlm.nih.gov/nucleotide/OP964268.1?report=genbank&log$=nucltop&blast_rank=1&RID=ZD4UK4WY013) |
| *Ny*. *yuilli pajoti* | 7 | 2 | 1 | 0.286 | 0.000 | 0.000 | 0.000 | 0.000 | *Ps*. *davisi* | 9.6 | 10.4 | 100 - 99.5 | [OP964289.1](https://www.ncbi.nlm.nih.gov/nucleotide/OP964289.1?report=genbank&log$=nucltop&blast_rank=1&RID=ZD72W3HD016) |
| *Pa*. (*Psa*.) *dendrophyla* | 1 | 1 | 0 | 0.000 | 0.000 | 0.000 | 0.000 | 0.000 | *Ps*. *davisi* | 11.3 | 12.5 | 98.5 - 98.3 | [OP964328.1](https://www.ncbi.nlm.nih.gov/nucleotide/OP964328.1?report=genbank&log$=nucltop&blast_rank=1&RID=ZG7R9Z8R013) |
| *Pa*. (*For*.) *aragaoi* | 6 | 3 | 20 | 0.600 | 0.012 | 0.012 | 0.012 | 0.012 | *Ny*. *fraihai* | 10.7 | 11.7 | 100 - 93.0 | [OP346809.1](https://www.ncbi.nlm.nih.gov/nucleotide/OP346809.1?report=genbank&log$=nucltop&blast_rank=1&RID=ZD8C74SU01R) |
| *Pi*. (*Pif*.) *nevesi* | 1 | 1 | 0 | 0.000 | 0.000 | 0.000 | 0.000 | 0.000 | *Ev*. (*Ald*.) *walkeri* | 15.5 | 17.6 | 96.9 | [OQ922568.1](https://www.ncbi.nlm.nih.gov/nucleotide/OQ922568.1?report=genbank&log$=nucltop&blast_rank=1&RID=70XXMNX8016) |
| *Ps*. *amazonensis* | 1 | 1 | 0 | 0.000 | 0.000 | 0.000 | 0.000 | 0.000 | *Ps*. *davisi* | 7.1 | 7.6 | 98.9 - 92.9 | [OP346812.1](https://www.ncbi.nlm.nih.gov/nucleotide/OP346812.1?report=genbank&log$=nucltop&blast_rank=1&RID=ZG25K2CN01R) |
| *Ps*. *ayrozai* | 6 | 5 | 16 | 0.933 | 0.016 | 0.016 | 0.016 | 0.016 | *Ps*. *carrerai thula* | 0.6 | 0.6 | 97.7 - 93.9 | [OP964336.1](https://www.ncbi.nlm.nih.gov/nucleotide/OP964336.1?report=genbank&log$=nucltop&blast_rank=1&RID=ZG49ECJK016) |
| *Ps*. *carrerai thula* | 2 | 2 | 1 | 1.000 | 0.002 | 0.002 | 0.002 | 0.002 | *Ps*. *davisi* | 10.1 | 11.0 | 89.9 - 89.6 | [AB984407.1](https://www.ncbi.nlm.nih.gov/nucleotide/AB984407.1?report=genbank&log$=nucltop&blast_rank=1&RID=ZG74HGRG013) |
| *Ps*. *chagasi* | 7 | 4 | 3 | 0.714 | 0.002 | 0.002 | 0.002 | 0.002 | *Ps*. *davisi* | 9.1 | 9.9 | 99.5 - 99.4 | [MH281893.1](https://www.ncbi.nlm.nih.gov/nucleotide/MH281893.1?report=genbank&log$=nucltop&blast_rank=1&RID=ZF17BEPZ013) |
| *Ps*. *davisi* | 10 | 9 | 36 | 0.978 | 0.026 | 0.026 | 0.026 | 0.026 | *Ps*. *carrerai thula* | 10.1 | 11.0 | 99.4 - 99.4 | [OL794300.1](https://www.ncbi.nlm.nih.gov/nucleotide/OL794300.1?report=genbank&log$=nucltop&blast_rank=1&RID=ZDHNGYJ5013) |
| *Ps*. *panamensis* | 1 | 1 | 0 | 0.000 | 0.000 | 0.000 | 0.000 | 0.000 | *Ps*. *davisi* | 10.5 | 11.6 | 97.1 | [GU001750.1](https://www.ncbi.nlm.nih.gov/nucleotide/GU001750.1?report=genbank&log$=nuclalign&blast_rank=1&RID=F1NZ6G77016) |
| *Ps*. *paraensis* | 3 | 1 | 0 | 0.000 | 0.000 | 0.000 | 0.000 | 0.000 | *Ps*. *davisi* | 9.3 | 10.1 | 99.2 | [OR555696.1](https://www.ncbi.nlm.nih.gov/nucleotide/OR555696.1?report=genbank&log$=nucltop&blast_rank=1&RID=ZEWBJETX01R) |
| *Sc*. *fluviatilis* | 1 | 1 | 0 | 0.000 | 0.000 | 0.000 | 0.000 | 0.000 | *Ps*. *ayrozai* | 12.6 | 14.0 | 93.8 | [OQ922795.1](https://www.ncbi.nlm.nih.gov/nucleotide/OQ922795.1?report=genbank&log$=nucltop&blast_rank=1&RID=4383D1ZA01N) |
| *Sc*. *sordellii* | 2 | 2 | 5 | 1.000 | 0.008 | 0.008 | 0.008 | 0.008 | *Lu*. (*Hel*.) *tortura* | 12.9 | 14.2 | 100 - 99.8 | [OP964349.1](https://www.ncbi.nlm.nih.gov/nucleotide/OP964349.1?report=genbank&log$=nuclalign&blast_rank=1&RID=438HH67A016) |
| *Th*. *howardi* | 3 | 3 | 14 | 1.000 | 0.015 | 0.015 | 0.015 | 0.015 | *Th*. *cellulana* | 1.0 | 1.0 | 99.2 - 98.7 | [OP964356.1](https://www.ncbi.nlm.nih.gov/nucleotide/OP964356.1?report=genbank&log$=nucltop&blast_rank=1&RID=ZF9Y14WS016) |
| *Th*. *velezbernali* | 1 | 1 | 0 | 0.000 | 0.000 | 0.000 | 0.000 | 0.000 | *Th*. *cellulana* | 0.2 | 0.2 | 99.8 | [OP964359.1](https://www.ncbi.nlm.nih.gov/nucleotide/OP964359.1?report=genbank&log$=nuclalign&blast_rank=1&RID=439F1280013) |
| *Ty*. *witoto* * | 3 | 1 | 0 | 0.000 | 0.000 | 0.000 | 0.000 | 0.000 | *Vi*. *caprina* | 10.9 | 11.8 | 0.0 | N/A |
| *Th*. *cellulana* | 14 | 5 | 4 | 0.593 | 0.001 | 0.001 | 0.001 | 0.001 | *Th*. *velezbernali* | 0.2 | 0.2 | 100 | [OP346840.1](https://www.ncbi.nlm.nih.gov/nucleotide/OP346840.1?report=genbank&log$=nucltop&blast_rank=1&RID=43A66ZU7013) |
| *Vi*. *caprina* * | 1 | 1 | 0 | 0.000 | 0.000 | 0.000 | 0.000 | 0.000 | *Ps*. *davisi* | 9.5 | 10.4 | 0.0 | N/A |
| *Vi*. *tuberculata* | 1 | 1 | 0 | 0.000 | 0.000 | 0.000 | 0.000 | 0.000 | *Vi*. *caprina* | 9.7 | 10.4 | 98.9 | [OQ922932.](https://www.ncbi.nlm.nih.gov/nucleotide/OQ922932.1?report=genbank&log$=nucltop&blast_rank=1&RID=438Y5THB016)1 |

**S2 Table**. Sand flies species, number of individuals with mitochondrial haplotypes, maximum intraspecific genetic divergence and minimum distance to nearest neighbor of the Colombian Amazonian sand flies species analyzed in this study.
