# Supplementary material for: Taxonomic, molecular and ecological approach reveals high diversity of vector sand flies, varied blood source supply and a high detection rate of Leishmania DNA in Colombian Amazon region
Source: PLoS Negl Trop Dis. 2025 Sep 5;19(9):e0013445. doi: 10.1371/journal.pntd.0013445 (PMC12412933; doi:10.1371/journal.pntd.0013445)
Supplement: S3 Table — (DOCX) [file pntd.0013445.s006.docx]

**S3 Table.** Vertebrates acting as sources of blood ingestion in sand flies detected with the molecular marker Ctyb in species collected in Amazonas and Caquetá, Colombian Amazon region.

| **Species** | **Vertebrates** | **Common name** | **Identity (%)** | **Coverage (%)** | **E_value** | **GenBank Code** |
| --- | --- | --- | --- | --- | --- | --- |
| *Ny*. *fraihai* | *Sus scrofa* | Domestic pig | 100 | 100 | 0.0 | [OQ982484.1](https://www.ncbi.nlm.nih.gov/nucleotide/OQ982484.1?report=genbank&log$=nucltop&blast_rank=1&RID=73FBF2ZF016) |
| *Ny*. *fraihai* | *Sus scrofa* | Domestic pig | 100 | 100 | 0.0 | [OQ982484.1](https://www.ncbi.nlm.nih.gov/nucleotide/OQ982484.1?report=genbank&log$=nucltop&blast_rank=1&RID=73FBF2ZF016) |
| *Ny*. *fraihai* | *Sus scrofa* | Domestic pig | 100 | 100 | 0.0 | [OQ982484.1](https://www.ncbi.nlm.nih.gov/nucleotide/OQ982484.1?report=genbank&log$=nucltop&blast_rank=1&RID=73G41AGN016) |
| *Ny*. *fraihai* | *Sus scrofa* | Domestic pig | 100 | 100 | 0.0 | [OQ982484.1](https://www.ncbi.nlm.nih.gov/nucleotide/OQ982484.1?report=genbank&log$=nucltop&blast_rank=1&RID=73KT0HAH013) |
| *Ev*. (*Ald*.) *walkeri* | *Sus scrofa* | Domestic pig | 100 | 100 | 0.0 | [OQ982484.1](https://www.ncbi.nlm.nih.gov/nucleotide/OQ982484.1?report=genbank&log$=nucltop&blast_rank=1&RID=73KT0HAH013) |
| *Th. cellulana* | *Sus scrofa* | Domestic pig | 100 | 100 | 2.00E-180 | [OQ982484.1](https://www.ncbi.nlm.nih.gov/nucleotide/OQ982484.1?report=genbank&log$=nucltop&blast_rank=1&RID=73G41AGN016) |
| *Ev*. (*Ald*.) *walkeri* | *Sus scrofa* | Domestic pig | 100 | 100 | 0.0 | [OQ982484.1](https://www.ncbi.nlm.nih.gov/nucleotide/OQ982484.1?report=genbank&log$=nucltop&blast_rank=1&RID=73G41AGN016) |
| *Ev*. (*Ald*.) *walkeri* | *Sus scrofa* | Domestic pig | 100 | 98 | 0.0 | [OQ982484.1](https://www.ncbi.nlm.nih.gov/nucleotide/OQ982484.1?report=genbank&log$=nucltop&blast_rank=1&RID=73G41AGN016) |
| *Th*. *cellulana* | *Sus scrofa* | Domestic pig | 100 | 98 | 0.0 | [OQ982484.1](https://www.ncbi.nlm.nih.gov/nucleotide/OQ982484.1?report=genbank&log$=nucltop&blast_rank=1&RID=73G41AGN016) |
| *Th*. *cellulana* | *Sus scrofa* | Domestic pig | 100 | 100 | 0.0 | [OQ982484.1](https://www.ncbi.nlm.nih.gov/nucleotide/OQ982484.1?report=genbank&log$=nucltop&blast_rank=1&RID=73G41AGN016) |
| *Ev*. (*Ald*.) *walkeri* | *Homo sapiens* | Human | 100 | 100 | 0.0 | [MG571125.1](https://www.ncbi.nlm.nih.gov/nucleotide/MG571125.1?report=genbank&log$=nucltop&blast_rank=1&RID=78BY34RA013) |
| *Th*. *cellulana* | *Homo sapiens* | Human | 100 | 100 | 0.0 | [MG571125.1](https://www.ncbi.nlm.nih.gov/nucleotide/MG571125.1?report=genbank&log$=nucltop&blast_rank=1&RID=78BY34RA013) |
| *Ev*. (*Ald*.) *walkeri* | *Homo sapiens* | Human | 100 | 100 | 0.0 | [MG571125.1](https://www.ncbi.nlm.nih.gov/nucleotide/MG571125.1?report=genbank&log$=nucltop&blast_rank=1&RID=78BY34RA013) |
| *Ny*. *yuilli pajoti* | *Homo sapiens* | Human | 99.7 | 98 | 0.0 | [MG571125.1](https://www.ncbi.nlm.nih.gov/nucleotide/MG571125.1?report=genbank&log$=nucltop&blast_rank=1&RID=78BY34RA013) |
| *Ny*. *yuilli pajoti* | *Homo sapiens* | Human | 100 | 100 | 0.0 | [MG571125.1](https://www.ncbi.nlm.nih.gov/nucleotide/MG571125.1?report=genbank&log$=nucltop&blast_rank=1&RID=78BY34RA013) |
| *Ny*. *yuilli pajoti* | *Homo sapiens* | Human | 99.7 | 98 | 0.0 | [MG571125.1](https://www.ncbi.nlm.nih.gov/nucleotide/MG571125.1?report=genbank&log$=nucltop&blast_rank=1&RID=78BY34RA013) |
| *Pi*. (*Pif*.) *nevesi* | *Dasyprocta leporina* | Brazilian agouti | 95.7 | 99 | 3.00E-144 | [AF437811.1](https://www.ncbi.nlm.nih.gov/nucleotide/AF437811.1?report=genbank&log$=nucltop&blast_rank=2&RID=A6PXMYU0016) |
| *Ny*. *yuilli pajoti* | *Homo sapiens* | Human | 99.7 | 100 | 3.00E-180 | [MG571125.1](https://www.ncbi.nlm.nih.gov/nucleotide/MG571125.1?report=genbank&log$=nucltop&blast_rank=1&RID=78BY34RA013) |
| *Ny*. *yuilli pajoti* | *Homo sapiens* | Human | 100 | 100 | 1.00E-142 | [MG571125.1](https://www.ncbi.nlm.nih.gov/nucleotide/MG571125.1?report=genbank&log$=nucltop&blast_rank=1&RID=78NHZ3RB016) |
| *Nyssomyia* sp. | *Homo sapiens* | Human | 99.7 | 100 | 3.00E-180 | [MG571125.1](https://www.ncbi.nlm.nih.gov/nucleotide/MG571125.1?report=genbank&log$=nucltop&blast_rank=1&RID=78NHZ3RB016) |
| *Ny*. *yuilli pajoti* | *Homo sapiens* | Human | 99.7 | 100 | 0.0 | [MG571125.1](https://www.ncbi.nlm.nih.gov/nucleotide/MG571125.1?report=genbank&log$=nucltop&blast_rank=1&RID=78NHZ3RB016) |
| *Ny*. *fraihai* | *Bos taurus* | Cow | 85.9 | 100 | 5.00E-77 | [EU365345.1](https://www.ncbi.nlm.nih.gov/nucleotide/EU365345.1?report=genbank&log$=nucltop&blast_rank=9&RID=A6S1KKHG013) |
| *Ny*. *yuilli pajoti* | *Homo sapiens* | Human | 99.7 | 100 | 2.00E-180 | [MG571125.1](https://www.ncbi.nlm.nih.gov/nucleotide/MG571125.1?report=genbank&log$=nucltop&blast_rank=1&RID=78NHZ3RB016) |
| *Ev*. (*Ald*.) *walkeri* | *Homo sapiens* | Human | 99.7 | 99 | 0.0 | [MG571125.1](https://www.ncbi.nlm.nih.gov/nucleotide/MG571125.1?report=genbank&log$=nucltop&blast_rank=1&RID=78NHZ3RB016) |
| *Ps*. *paraensis* | *Homo sapiens* | Human | 100 | 99 | 2.00E-145 | [MG571125.1](https://www.ncbi.nlm.nih.gov/nucleotide/MG571125.1?report=genbank&log$=nucltop&blast_rank=1&RID=78NHZ3RB016) |
| *Ev*. (*Ald*.) *walkeri* | *Homo sapiens* | Human | 99.7 | 100 | 2.00E-180 | [MG571125.1](https://www.ncbi.nlm.nih.gov/nucleotide/MG571125.1?report=genbank&log$=nucltop&blast_rank=1&RID=78NHZ3RB016) |
| *Ev*. (*Ald*.) *walkeri* | *Homo sapiens* | Human | 99.7 | 100 | 2.00E-180 | [MG571125.1](https://www.ncbi.nlm.nih.gov/nucleotide/MG571125.1?report=genbank&log$=nucltop&blast_rank=1&RID=78NHZ3RB016) |
| *Lutzomyia sp.* | *Homo sapiens* | Human | 99.7 | 99 | 0.0 | [MG571125.1](https://www.ncbi.nlm.nih.gov/nucleotide/MG571125.1?report=genbank&log$=nucltop&blast_rank=1&RID=78V5HN0B016) |
| *Ny*. *umbratilis* | *Homo sapiens* | Human | 99.6 | 98 | 4.00E-123 | [MG660589.1](https://www.ncbi.nlm.nih.gov/nucleotide/MG660589.1?report=genbank&log$=nucltop&blast_rank=1&RID=78V95KER013) |
