# Supplementary material for: Taxonomic, molecular and ecological approach reveals high diversity of vector sand flies, varied blood source supply and a high detection rate of Leishmania DNA in Colombian Amazon region
Source: PLoS Negl Trop Dis. 2025 Sep 5;19(9):e0013445. doi: 10.1371/journal.pntd.0013445 (PMC12412933; doi:10.1371/journal.pntd.0013445)
Supplement: S4 Table — (DOCX) [file pntd.0013445.s007.docx]

**S4 Table**. Vertebrates acting as sources of blood ingestion in sand flies detected with the molecular marker 12S in species collected in Amazonas and Caquetá, Colombian Amazon region.

| **Species** | **Vertebrates** | **Common name** | **Identity (%)** | **Coverage (%)** | **E_value** | **GenBank Code** |
| --- | --- | --- | --- | --- | --- | --- |
| *Ny*. *fraihai* | *Sus scrofa* | Domestic pig | 100 | 100 | 1.00E-54 | [MT253545.1](https://www.ncbi.nlm.nih.gov/nucleotide/MT253545.1?report=genbank&log$=nucltop&blast_rank=1&RID=A5HZBHG9013) |
| *Ny*. *fraihai* | *Sus scrofa* | Domestic pig | 100 | 99.0 | 5.00E-106 | [MN258706.1](https://www.ncbi.nlm.nih.gov/nucleotide/MN258706.1?report=genbank&log$=nucltop&blast_rank=2&RID=A5J5FJS3016) |
| *Ny*. *fraihai* | *Sus scrofa* | Domestic pig | 100 | 99.0 | 6.00E-105 | [MN258706.1](https://www.ncbi.nlm.nih.gov/nucleotide/MN258706.1?report=genbank&log$=nucltop&blast_rank=2&RID=A5J5FJS3016) |
| *Ev*. (*Ald*.) *walkeri* | *Cheracebus lugens* | White-chested Titi | 82.0 | 99.0 | 3.00E-16 | [OM328972.1](https://www.ncbi.nlm.nih.gov/nucleotide/OM328972.1?report=genbank&log$=nucltop&blast_rank=1&RID=A67BAZGE013) |
| *Th. cellulana* | *Homo sapiens* | Human | 100 | 95.0 | 3.00E-55 | [MG244225.1](https://www.ncbi.nlm.nih.gov/nucleotide/MG244225.1?report=genbank&log$=nucltop&blast_rank=1&RID=73P077C1016) |
| *Th. cellulana* | *Sus scrofa* | Domestic pig | 100 | 90.0 | 6.00E-63 | [MN258706.1](https://www.ncbi.nlm.nih.gov/nucleotide/MN258706.1?report=genbank&log$=nucltop&blast_rank=2&RID=A5J5FJS3016) |
| *Ny*. *yuilli pajoti* | *Homo sapiens* | Human | 100 | 91.0 | 2.00E-53 | [MG244225.1](https://www.ncbi.nlm.nih.gov/nucleotide/MG244225.1?report=genbank&log$=nucltop&blast_rank=1&RID=73P077C1016) |
| *Th. cellulana* | *Homo sapiens* | Human | 100 | 94.0 | 2.00E-58 | [MF437201.1](https://www.ncbi.nlm.nih.gov/nucleotide/MF437201.1?report=genbank&log$=nucltop&blast_rank=1&RID=A5KVD40K013) |
| *Th. cellulana* | *Felis catus* | Domestic cat | 96.9 | 81.0 | 4.00E-50 | [OR085764.1](https://www.ncbi.nlm.nih.gov/nucleotide/OR085764.1?report=genbank&log$=nucltop&blast_rank=3&RID=A64KDVSE013) |
| *Ny*. *yuilli pajoti* | *Homo sapiens* | Human | 98.0 | 100 | 4.00E-70 | [MF437201.1](https://www.ncbi.nlm.nih.gov/nucleotide/MF437201.1?report=genbank&log$=nucltop&blast_rank=1&RID=A5KVD40K013) |
| *Ny*. *antunesi* | *Homo sapiens* | Human | 100 | 100 | 1.00E-73 | [MG244225.1](https://www.ncbi.nlm.nih.gov/nucleotide/MG244225.1?report=genbank&log$=nucltop&blast_rank=1&RID=73P077C1016) |
| *Pi*. (*Pif*.) *nevesi* | *Homo sapiens* | Human | 97.0 | 100 | 1.00E-69 | [MG244225.1](https://www.ncbi.nlm.nih.gov/nucleotide/MG244225.1?report=genbank&log$=nucltop&blast_rank=1&RID=73P077C1016) |
| *Ny*. *yuilli pajoti* | *Homo sapiens* | Human | 96.8 | 64.0 | 1.00E-34 | [MG244225.1](https://www.ncbi.nlm.nih.gov/nucleotide/MG244225.1?report=genbank&log$=nucltop&blast_rank=1&RID=73P077C1016) |
| *Ev*. (*Ald*.) *walkeri* | *Homo sapiens* | Human | 100 | 94.0 | 1.00E-65 | [MG244225.1](https://www.ncbi.nlm.nih.gov/nucleotide/MG244225.1?report=genbank&log$=nucltop&blast_rank=1&RID=73P077C1016) |
| *Sc*. *sordellii* | *Homo sapiens* | Human | 100 | 100 | 5.00E-74 | [MG244225.1](https://www.ncbi.nlm.nih.gov/nucleotide/MG244225.1?report=genbank&log$=nucltop&blast_rank=1&RID=73P077C1016) |
| *Ev*. (*Ald*.) *walkeri* | *Homo sapiens* | Human | 92.8 | 100 | 3.00E-46 | [MG244225.1](https://www.ncbi.nlm.nih.gov/nucleotide/MG244225.1?report=genbank&log$=nucltop&blast_rank=1&RID=73P077C1016) |
| *Ps*. *davisi* | *Saimiri macrodon* | Squirrel monkey | 98.8 | 54.0 | 6.00E-34 | [NC_064183.1](https://www.ncbi.nlm.nih.gov/nucleotide/NC_064183.1?report=genbank&log$=nucltop&blast_rank=3&RID=A62MMY4G013) |
| *Ps*. *paraensis* | *Homo sapiens* | Human | 100 | 98.0 | 4.00E-70 | [MG244225.1](https://www.ncbi.nlm.nih.gov/nucleotide/MG244225.1?report=genbank&log$=nucltop&blast_rank=1&RID=73P077C1016) |
