# Supplementary material for: Taxonomic, molecular and ecological approach reveals high diversity of vector sand flies, varied blood source supply and a high detection rate of Leishmania DNA in Colombian Amazon region
Source: PLoS Negl Trop Dis. 2025 Sep 5;19(9):e0013445. doi: 10.1371/journal.pntd.0013445 (PMC12412933; doi:10.1371/journal.pntd.0013445)
Supplement: S5 Table — (DOCX) [file pntd.0013445.s008.docx]

**S5 Table**. Prevalence of *Leishmania* infection and minimum infection rate (MIR) in sand flies collected in the Colombian Amazon region.

| **Species** | **Specimens analyzed** | **Specimens positives for *Leishmania*** | **Infection prevalence** | **Minimal Infection Rate (MIR)** |
| --- | --- | --- | --- | --- |
| *Ev*. (*Ald*.) *walkeri* | 36 | 3 | 10.7 | 0.97 |
| *Ev*. (*Eva*.) *georgii* | 2 | 0 | 0.0 | 0.0 |
| *Lu*. (*Hel*.) *tortura* | 4 | 2 | 7.1 | 0.7 |
| *Lu*. (*Tri*.) *sherlocki* | 1 | 1 | 3.6 | 0.3 |
| *Ny*. *antunesi* | 42 | 0 | 0.0 | 0.0 |
| *Ny*. *fraihai* | 48 | 8 | 28.6 | 2.6 |
| *Ny*. *yuilli pajoti* | 13 | 4 | 14.3 | 1.3 |
| *Ps*. *ayrozai* | 8 | 1 | 3.6 | 0.3 |
| *Ps*. *chagasi* | 12 | 1 | 3.6 | 0.3 |
| *Ps*. *paraensis* | 20 | 1 | 3.6 | 0.3 |
| *Pi*. (*Pif*.) *nevesi* | 1 | 1 | 3.6 | 0.3 |
| *Th. cellulana* | 47 | 4 | 14.3 | 1.3 |
| *Vi*. *caprina* | 1 | 1 | 3.6 | 0.3 |
| *Nyssomyia* sp. | 2 | 1 | 3.6 | 0.3 |
| Other species | 73 | 0 | 0.0 | 0.0 |
| **Total** | **310** | **28** | **100** | **9**.**0** |

**Infection prevalence=**Sand flies positives for species X 100/Total sand flies positives

**Minimal Infection Rate (MIR) =** Sand flies positives for species/Total sand flies analyzed X 100
