# Supplementary material for: Taxonomic, molecular and ecological approach reveals high diversity of vector sand flies, varied blood source supply and a high detection rate of Leishmania DNA in Colombian Amazon region
Source: PLoS Negl Trop Dis. 2025 Sep 5;19(9):e0013445. doi: 10.1371/journal.pntd.0013445 (PMC12412933; doi:10.1371/journal.pntd.0013445)
Supplement: Attachment — Submitted filename: PNTD-D-25-00172_revised.pdf [file pntd.0013445.s011.pdf]

PLOS Neglected Tropical Diseases

Taxonomic, molecular and ecological approach reveals high diversity of vector phlebotomine sand flies, varied blood source supply and a high detection rate of Leishmania DNA in Colombian Amazon region

--Manuscript Draft--

**Manuscript Number:**	PNTD-D-25-00172
**Full Title:**	Taxonomic, molecular and ecological approach reveals high diversity of vector phlebotomine sand flies, varied blood source supply and a high detection rate of Leishmania DNA in Colombian Amazon region
**Short Title:**	Record of Sciopemyia fluviatilis in Colombia
**Article Type:**	Research Article
**Keywords:**	Blood meal, integrative taxonomy, leishmaniasis, natural infection, Nyssomyia
**Abstract:**	Background<br>The Amazon region is home to more than 30% of the phlebotomine sandfly species in Colombia, including vectors of Leishmania mainly in the genus Lutzomyia and Psychodopygus. Advances in morphological and molecular taxonomy of phlebotomine sand flies facilitate the development of updated and robust species inventories in understudied areas, such as the departments of Amazonas and Caquetá. Currently, integrating the detection of blood meal sources and Leishmania DNA represents a key approach under the “One Health” concept by providing insights into human and animal health and the dynamics of different ecosystems.<br>Methodology/Principal findings<br>This study characterized the sandfly fauna in Amazonas and Caquetá using an integrative taxonomic approach that included DNA detection from blood meal and Leishmania sources. Phlebotomine sand flies were collected using CDC, Shannon, Prokopack® traps and mouth aspirators. DNA was analyzed by conventional PCR targeting COI, Cytb, 12S rDNA and HSP-70N markers, respectively. A total of 1,104 specimens were collected, representing 12 genera and 30 species, eleven are recognized vectors of Leishmania, including Nyssomyia antunesi and Psychodopygus amazonensis. Our findings include new reports of regional distribution, particularly the first report of Sciopemyia fluviatilis in Colombia. Homo sapiens (28.81% Cytb; 18.64% 12S) and Sus scrofa (16.94% Cytb; 6.77% 12S) were the main food sources detected. While Nyssomyia fraihai (2,581%), Trichophoromyia auraensis (1,290%), Nyssomyia yuilli pajoti (1,290%) and Evandromyia (Aldamyia) walkeri (0,968%) grouped the highest detection rate of Leishmania DNA (9.032%).<br>Conclusions/Significance<br>The integration of molecular tools for the confirmation of phlebotomine species allowed the resolution of taxonomic conflicts, especially in the genus Trichophoromyia. These findings provide key information on ecological interactions (vectors-ingesta-Leishmania) related to leishmaniasis in the Colombian Amazon, suggesting a high diversity of phlebotomine sand flies and a significant zoonotic potential.
**Additional Information:**	
**Question**	**Response**
**Government Employee**<br><br>Are you or any of the contributing authors an employee of the United States government?<br><br>Manuscripts authored by one or more US Government employees are not copyrighted, but are licensed under a CC0	No - No authors are employees of the U.S. government.

Powered by Editorial Manager® and ProduXion Manager® from Aries Systems Corporation

Public Domain Dedication, which allows unlimited distribution and reuse of the article for any lawful purpose. This is a legal requirement for US Government employees.<br><br>This will be typeset if the manuscript is accepted for publication.	
**Financial Disclosure**<br><br>Enter a financial disclosure statement that describes the sources of funding for the work included in this submission. Review the submission guidelines for detailed requirements. View published research articles from *PLOS Neglected Tropical Diseases* for specific examples.<br><br>This statement is required for submission and **will appear in the published article** if the submission is accepted. Please make sure it is accurate.<br><br>**Funded studies**<br>Enter a statement with the following details:<br>• Initials of the authors who received each award<br>• Grant numbers awarded to each author<br>• The full name of each funder<br>• URL of each funder website<br>• Did the sponsors or funders play any role in the study design, data collection and analysis, decision to publish, or preparation of the manuscript?<br><br>Did you receive funding for this work?	Yes
Please add funding details.<br>as follow-up to "**Financial Disclosure**<br><br>Enter a financial disclosure statement that describes the sources of funding for the work included in this submission. Review the submission guidelines for detailed requirements. View published research articles from *PLOS Neglected Tropical Diseases* for specific examples.	This work was supported and financed by the project Hermes 57545 of the Universidad Nacional de Colombia, and framework of Call 15: Formation of High-Level Human Capital for the Department of Caquetá through a master's degree scholarship of MINCIENCIAS.

This statement is required for submission and **will appear in the published article** if the submission is accepted. Please make sure it is accurate.<br><br>**Funded studies**<br>Enter a statement with the following details:<br>• Initials of the authors who received each award<br>• Grant numbers awarded to each author<br>• The full name of each funder<br>• URL of each funder website<br>• Did the sponsors or funders play any role in the study design, data collection and analysis, decision to publish, or preparation of the manuscript?<br><br>Did you receive funding for this work?"	
Please select the country of your main research funder (please select carefully as in some cases this is used in fee calculation).<br>as follow-up to "**Financial Disclosure**<br><br>Enter a financial disclosure statement that describes the sources of funding for the work included in this submission. Review the submission guidelines for detailed requirements. View published research articles from *PLOS Neglected Tropical Diseases* for specific examples.<br><br>This statement is required for submission and **will appear in the published article** if the submission is accepted. Please make sure it is accurate.	COLOMBIA - CO

**Funded studies**<br>Enter a statement with the following details:<br>• Initials of the authors who received each award<br>• Grant numbers awarded to each author<br>• The full name of each funder<br>• URL of each funder website<br>• Did the sponsors or funders play any role in the study design, data collection and analysis, decision to publish, or preparation of the manuscript?<br><br>Did you receive funding for this work?"	
**Competing Interests**<br><br>On behalf of all authors, disclose any competing interests that could be perceived to bias this work.<br><br>This statement will be typeset if the manuscript is accepted for publication.<br><br>Review the instructions link below and PLOS NTDs' competing interests policy to determine what information must be disclosed at submission.	The authors declare that they have no conflict of interest that would interfere with publication.
**Data Availability**<br><br>Provide a **Data Availability Statement** in the box below. This statement should detail where the data used in this submission can be accessed. This statement will be typeset if the manuscript is accepted for publication.<br><br>Before publication, authors are required to make all data underlying their findings fully available, without restriction. Review our PLOS Data Policy page for detailed information on this policy. Instructions for writing your Data Availability statement can be accessed via the Instructions link below.	The sequence data associated with the COI,Cytb marker, 12S marker are available in the GenBank database.

Manuscript
Click here to access/download;Manuscript;ART1_PNTD_KCvied_0225.docx

RESEARCH ARTICLE

Taxonomic, molecular and ecological approach reveals high diversity of vector phlebotomine sand flies, varied blood source supply and a high detection rate of *Leishmania* DNA in Colombian Amazon region

Record of *Sciopemyia fluviatilis* in Colombia

Katerine Caviedes-Triana[1-2], Daniela Duque-Granda[1], Gloria Cadavid-Restrepo[1], Claudia X. Moreno-Herrera[1*], Rafael Vivero-Gomez[1,2*]

[1]Grupo de Microbiodiversidad y Bioprospección, Faculty of Sciences, Universidad Nacional de Colombia, Medellín, Colombia. Street 59 A # 63-20, Medellín Postal Code 050003
[2]PECET (Programa de Estudio y control de enfermedades tropicales), Facultad de Medicina, Universidad de Antioquia, Medellín, Colombia

Caviedes-Triana: https://orcid.org/0009-0005- 5838-3782, Duque-Granda: https://orcid.org/0000-0002-4994-2686, Cadavid-Restrepo: https://orcid.org/0000-0003-1026-6358, Vivero-Gómez: https://orcid.org/0000-0001-6768-4097, Moreno-Herrera: https://orcid.org/0000-0002-8132-5223

*Correspondence: rjviverog@unal.edu.co, R.J.V.G.; cxmoreno@unal.edu.co, C.X.M.H.

Abstract

Background
The Amazon region is home to more than 30% of the phlebotomine sandfly species in Colombia, including vectors of *Leishmania* mainly in the genus *Lutzomyia and Psychodopygus.* Advances in morphological and molecular taxonomy of phlebotomine sand flies facilitate the development of updated and robust species inventories in understudied areas, such as the departments of Amazonas and Caquetá. Currently, integrating the detection of blood meal sources and *Leishmania* DNA represents a key approach under the "One Health" concept by providing insights into human and animal health and the dynamics of different ecosystems.

Methodology/Principal findings
This study characterized the sandfly fauna in Amazonas and Caquetá using an integrative taxonomic approach that included DNA detection from blood meal and *Leishmania* sources. Phlebotomine sand flies were collected using CDC, Shannon, Prokopack® traps and mouth aspirators. DNA was analyzed by conventional PCR targeting COI, Cytb, 12S rDNA and HSP-70N markers, respectively.

A total of 1,104 specimens were collected, representing 12 genera and 30 species, eleven are recognized vectors of *Leishmania*, including *Nyssomyia antunesi* and *Psychodopygus amazonensis*. Our findings include new reports of regional distribution, particularly the first report of *Sciopemyia fluviatilis* in Colombia.

Homo sapiens (28.81% Cytb; 18.64% 12S) and *Sus scrofa* (16.94% Cytb; 6.77% 12S) were the main food sources detected. While *Nyssomyia fraihai* (2,581%), *Trichophoromyia auraensis* (1,290%), *Nyssomyia yuilli pajoti* (1,290%) and *Evandromyia (Aldamyia) walkeri* (0,968%) grouped the highest detection rate of *Leishmania* DNA (9.032%).

Conclusions/Significance
The integration of molecular tools for the confirmation of phlebotomine species allowed the resolution of taxonomic conflicts, especially in the genus *Trichophoromyia*. These findings provide key information on ecological interactions (vectors-ingesta-*Leishmania*) related to leishmaniasis in the Colombian Amazon, suggesting a high diversity of phlebotomine sand flies and a significant zoonotic potential.

Key words: Blood meal, integrative taxonomy, leishmaniasis, natural infection, *Nyssomyia*.

Abstract graphic

1. Introduction

Leishmaniases are a group of zoonotic diseases caused by *Leishmania (Le.) Leishmania (Le.) amazonensis, Le. Viannia (Vi.) braziliensis, Le. (Vi.) guyanensis, Le. (Le.) mexicana*, and *Le. (Vi.) panamensis* in the Colombian Amazon region [1,2]. These parasite species are transmitted by hematophagous Diptera belonging to the subfamily Phlebotominae Rondani, 1840. A significant number of leishmaniasis cases (4,403) were confirmed in the Colombian Amazon region alone, of which 37.50% (1,651 cases) were recorded in Amazonas and Caquetá departments during the period of 2020-2024 [3,4]. The fauna and flora biodiversity, the variety of ecologically distinct landscape features, as well as the constant changes in land use in the Amazon region, especially in both departments [5,6], allow the optimal development of phlebotomine sand flies and the increase of pathogen transmission risk, not only of parasites, but also of viruses and bacteria associated with these vectors [6–8].

In this context, human mobility between forest and jungle areas, driven by tourism, forced displacement, and cross-border economic activities, has led to significant disturbances in natural habitats. Additionally, deforestation, illegal mining, and extensive cattle ranching aggravate the problem of vector-borne diseases (ETVs) such as leishmaniasis [9,10].

This has favored the expansion of the geographical ranges and ecological niches of phlebotomine sand flies [11], increasing their interaction with wild and domestic vertebrates, which play a role in the epidemiological triad as food sources, but also as potential reservoirs of *Leishmania* [10,12,13] and/or other microorganisms of public health interest such as *Bartonella bacilliformis* [14].

Recent studies conducted in urban forest remnants and preserved forest environments in the Brazilian Amazon evidenced how the eclectic feeding behavior of phlebotomine sand flies in disturbed habitats increased the risk of contact with vertebrate reservoirs like *Psychodopygus (Ps.) ayrozai* (Barretto & Coutinho, 1940) which, besides resenting blood traces of *Homo (H.) sapiens*, was also associated with other sources of ingestion

such as *Dasypus novemcinctus*, a potential reservoir of *Le. (Vi.) naiffí* [15].

Other studies have detected blood traces of mammals such as *Caniculus paca* (lowland paca), a potential reservoir of *Le.* (*Vi.*) *lainsoni* [16] and *Tamandua (T.) tetradactyla* (collared anteater), proven host of *Le. (Vi). guyanensis* [17], in phlebotomine sand flies of *Nyssomyia (Ny.) antunesi* (Coutinho, 1939) [16]. *Trypanosoma minasense* DNA has also been detected in individuals of this species along with blood traces of *T. tetradactyla* and *H. sapiens* [15]. Feeding plasticity in these insects favors their survival and establishment in recently colonized habitats or areas where the primary food source is scarce [18,19] as demonstrated in *Ny. umbratilis* (Ward & Fraiha, 1977), *Lutzomyia (Lu.) gomezi* (Nitzulescu, 1931), and *Ny. antunesi* recorded in Amazonas and Caquetá [20]. Notably, these insects have also been found infected with different *Leishmania* species [21–23].

Due to the above, it is imperative to expand studies on blood ingestion in phlebotomine sand flies, which play a critical role as primary or secondary vectors, and that are involved in the increase of cutaneous leishmaniasis (CL) transmission risk, mainly in urban and rural areas of the Colombian Amazon [24,25]. Understanding the ecological interactions occurring in the Amazon region is essential to elucidate the *Leishmania* transmission cycle and assess whether habitat disturbances have facilitated the geographic expansion of vectors, parasites, and vertebrate hosts, especially in border areas [26], considering that previous studies have been conducted in the Andean and Caribbean regions of Colombia and in the Brazilian Amazon, demonstrating how the habitat disturbance of these insects favors the feeding plasticity of species such as *Ps. panamensis* (Shannon, 1926) and *Ny. antunesi*, while the Colombian Amazon region remains understudied in this aspect [16,18,19,27,28].

Specifically in the departments of Amazonas and Caquetá, previous research has focused on the distribution and diversity of phlebotomine sand flies without evaluating the role of these species in the transmission dynamics of the disease [29–31]. Although more than 46 phlebotomine sand flies species have been recorded in these departments, habitat disturbance activities suggest an expansion in their distribution ranges, highlighting the need for an increase in entomological surveys and comprehensive taxonomic inventories in a more holistic manner [11].

Entomological studies in the Caquetá department are limited to the year 2000, where species identification was conducted by comparing morphological characters [24]. Although with this approach it is possible to assign species, there are limitations associated with the presence of cryptic species mainly located within the genera *Psychodopygus* (Mangabeira, 1941), *Nyssomyia* (Barretto, 1962) and *Trichophoromyia* (Barretto, 1962). Among the limitations are the collection of individuals of a single sex, isomorphisms in female spermathecae, species complexes, the slow integration of newly described species into taxonomic keys and the use of ambiguous taxonomic characters that limit the correct differentiation of species [29]. For this reason, it is necessary to implement an approach that integrates classical and molecular taxonomy, referred to as "integrative taxonomy", through the use of DNA barcoding and specifically the analysis of partial sequences of the Cytochrome Oxidase Subunit I (COI) gene that serves as a tool for the rapid and accurate identification and delimitation of phlebotomine species at different taxonomic levels and with different degrees of complexity [32]. Despite the demonstrated efficacy of DNA barcoding for the precise identification of species, particularly in groups with isomorphic females [33–35], this methodology has not been adopted by local health entities, as resource allocation tends to prioritize the surveillance of other diseases such as malaria and dengue due to their high incidence and public health impact.

In this regard, studies such as the one conducted by Posada-López *et al.* are significant, as using integrative taxonomy from specimens collected in Leticia, Amazonas, led to the identification of 61 new mitochondrial haplotypes associated with 24 species and, for the first time, assigned Barcode sequences of *Evandromyia (Ev.) (Evandromyia)(Eva.) georgii* (Freitas & Barrett, 2002), *Psathyromyia (Pa.) (Psathyromyia)(Psa.) punctigeniculata* (Floch & Abonnenc, 1944) and *Ny. yuilli pajoti* (Abonnenc, Léger& Fauran 1979) [29]. Although it is currently the only study integrating classical and molecular taxonomy in the region, the results highlight the need for further research to expand the availability of sequences, particularly of missing species or those of epidemiological importance in the Amazon. This is especially critical in the department of Caquetá, where studies are even more limited.

Given the low number of sand fly studies recorded in the Colombian Amazon region and

the current relevance of this region, which is highly susceptible to climate change and experiences high circulation of pathogens due to constant human and animal migration, it is crucial to provide new information to better understand the dynamics and risk of leishmaniasis transmission. In this context, the present study characterized the sandfly fauna associated with rural localities in Leticia, Amazonas, and Florencia, Caquetá, using an integrative taxonomy approach (taxonomical keys and COI as barcode), including the detection of *Leishmania* DNA, and the identification of blood ingestion sources through different molecular markers.

2. Materials and methods

Collection permits

The collection in the Amazonas and Caquetá departments was conducted under the Framework Permit for Collecting Specimens of Wild Species of Biological Diversity for Non-commercial Scientific Research Purposes, granted by the Autoridad Nacional de Licencias Ambientales (ANLA) to the Universidad Nacional de Colombia by Resolution No. 0255 of March 14, 2014 (article 3), in accordance with the Decree No. 1376, 2013 of the Ministerio de Ambiente y Desarrollo Sostenible, considering the mobility certificate 59453 and with prior consent of the landowners and indigenous community.

2.1 Study area

An entomological survey was conducted in San Pedro de los Lagos (S: -4.142304, O: -69.953815) and Tanimboca (S: -4.127948276, O: -69.95326736) localities of Leticia, Amazonas, and in Jericó (N:1.72855556, W: -75.64913), Sebastopol (N: 1.65894444, W: -75.605111), Santo Domingo (N: 1.59536111, W: -75.657417) localities and in the Amazonian Research Center-CIMAZ-Macagual (N: 1.61750000, W: -75.616750) of Florencia, Caquetá (Fig 1).

The study area is part of the Colombian Amazon region, characterized by the presence of tropical rainforest (Bh-T) (Holdridge, 1996). The average annual temperature in the department of Amazonas ranges between 25.3 °C and 25.7 °C, the annual rainfall varies between 2,660 mm and 3,538 mm and the relative humidity is above 80% [36]. In the department of Caquetá, the average temperature ranges from 24.8 °C to 25.9 °C, the

annual average precipitation varies between 4,385 mm and 2,483 mm and relative humidity is above 80% [36]. The most important productive activities in the region are livestock, agriculture, forestry, mining, ornamental fishing, and border trade [9,37].

sand fly collection was conducted in secondary and primary forests. In the department of Amazonas, near the collection area, cassava and sugarcane crops, palm and fruit trees, domestic animals such as *Canis (C.) lupus* (dogs), *Felis (F.) catus* (cats) and *Gallus (G.) gallus* (hens) were observed. In the department of Caquetá, traps were placed in areas near vegetable gardens, arazá crops, cocoa, coffee, and banana plantations, all located close to natural water sources, and with presence of domestic animals such as *Sus (S.) scrofa* (domestic pig), *C. lupus*, *F catus*, *G. gallus*, and *Oryctolagus cuniculus* (rabbits). However, in both departments, no evidence of animals associated with livestock was observed.

Sampling sites selection was made considering access conditions to the area, the history of leishmaniasis cases reported by health departments [38] and the existence of favorable microenvironments for sand fly development.

Fig 1. Geographic location of the study area and phlebotomine sand fly collection sites: (A) Departments of Amazonas and Caquetá, Colombia, South America. (B) Municipality of Florencia, locality Jericó, Macagual, Santo Domingo and Sebastopol. (C) Municipality of Leticia, locality Leticia, San Pedro de los Lagos and Tanimboca. Source of layers [39–41] and Qgis, v. 3.34.12 [42].

2.3 Sand fly collection and processing

Two collection campaigns were performed in Caquetá and Amazonas departments in August and November 2023 respectively, for three consecutive days per sampling area. Twelve CDC (Centers for Disease Control and Prevention) light traps were installed for 12 hours (18:00 to 6:00) in the intra, peri and extra-domicile. Each collection point was georeferenced with a GPSmap_60CSx, GARMIN. A Shannon trap was installed between 18:00 and 19:00 for one night at the point of major collection, while active search was conducted day and night in resting phlebotomine sites with entomological aspirators and Prockopack® in potential spots such as broiler houses or poultry farms. Specimens were stored and transported dry in 1.5 ml tubes, in racks with silica gel, and then to the

laboratory of medical entomology of the Program for the Study and Control of Tropical Diseases (PECET), from the Universidad de Antioquia where all samples were stored at -20 °C until processing.

Collected insects were fragmented for taxonomic identification. The head and the last three abdominal segments were mounted in Euparal medium (Carl Roth GmbH + Co. KG, Alemania) for taxonomic allocation using the Galati 2024 nomenclature system [43], supported by the key of Young and Duncan 1994 [44] and bibliographical records of phlebotomine sand flies in the Colombian and Brazilian Amazon region. Some identified specimens were deposited in the Francisco Luis Gallego - MEFLG Entomological Museum of the Universidad Nacional de Colombia, at Medellín. Codes: 5089 to 65091, 65079-65081, 65082-65088, 65142-65146, 65147-65156, 65160-65162, 65157-65159, 65141, 65078). The use of generic abbreviations was conducted following the scheme proposed by Marcondes, 2007 [45].

2.4 Molecular analyses: DNA extraction of phlebotomine sand flies

After sand fly identification by classical taxonomy, DNA extraction was performed from the thorax, legs, wings, and proximal abdominal segments of specimens using a high salt concentration protocol [46]. The extraction process included mechanical lysis with pistil, thermal shock lysis, protein precipitation with proteinase K (Scientific Inc., Waltham, MA, USA), washing with alcohol at 70 and 96%, and final elution in TE Buffer (1X). DNA concentrations were determined using a NanoDrop spectrophotometer (IMPLEN, Germany) and stored at -20 ºC for molecular identification, blood source and *Leishmania* detection.

2.4.1 PCR for phlebotomine barcoding

The representation and total DNA of four females and four males of each phlebotomine species previously identified by classical taxonomy was considered, using partial amplification of the COI gene, using the primers LCO1490 5'-GGTCAACAAATCATAAAGATATTGG-3' and HCO2198 5'-TAAACTTCA GGGTGACCAAAAAATC A-3' [47], amplifying a product of approximately 710 base pairs (bp). PCR reaction contained 1X buffer, 0.25 mM $MgCl_2$, 0.2 mM dNTPs, 0.4 µM of each oligonucleotide (forward and reverse), 1.5 U of Taq DNA polymerase (Excel taq,

SMOBIO Technology, Hsinchu Science Park, Taiwan), 1mg/ml BSA (Scientific Inc., Waltham, MA, USA), 10-25 ng/µl of total DNA and ultrapure water until a final reaction volume of 25 µl was completed (28). *Ny. antunesi* DNA was included as a positive control and ultrapure water as a negative control. The thermal profile consisted of an initial cycle of 1 min at 94°C, 1 min at 58°C and 2 min at 72°C followed by 35 cycles of 15 s at 94°C, 1 min at 58°C, 1.30 min at 72°C and finally a cycle of 15 s at 94°C, 1 min at 58°C, 7 min at 72°C [33].

2.4.2 Blood-meal source identification

DNA from the previously identified phlebotomine females with evidence of blood traces was amplified using the ribosomal 12S gene with the primers L1085 5'-CCCAAACTGGGATTAGATACCC-3' and H1259 5'-GTTTGCTGAAGATGGCGGTA-3' amplifying a fragment of approximately 215 bp [48]. Additionally, a partial region of the Cytochrome B gene (Cytb) was targeted using the primers L14841 5'-AAAAAGCTTCCATCAACATC-3' and H15148 5'-AAACTGCAGCCCCTCAGGATTTTGTTCCTC-3' that amplify a fragment of approximately 305 bp [49]. PCR reaction contained 1X buffer, 1.5 mM $MgCl_2$, 0.2 mM dNTPs, 0.5 µM of each oligonucleotide (forward and reverse), 1.5 U of Taq DNA polymerase (Excel taq, SMOBIO Technology, Hsinchu Science Park, Taiwan) 1mg/ml BSA (Scientific Inc., Waltham, MA, USA), 10-25 ng/µl of total DNA and ultrapure water until a final reaction volume of 25 µl was completed. The thermal cycling conditions consisted of an initial cycle of 5 min at 95°C, 35 cycles of 30 s at 95°C, 15 s at 57°C, 30 s at 72°C and finally a 10 min cycle at 72°C [50]. DNA from *Aedes (Ae.) aegypti* fed with *H. sapiens* and *C. lupu*s was used as a positive control and *Ae. aegypti* Rockefeller strain without blood meal ingestion, donated by the medical entomology laboratory of PECET, was also included as a negative control.

2.4.3 Molecular detection of *Leishmania* DNA

The N-terminal region of the gene encoding the heat shock protein 70 (*hsp-70*) was amplified from the DNA of 37.9% of all females collected (with and without evidence of blood ingestion) to detect the presence of *Leishmania* DNA. These specimens were

then organized into 168 samples, of which 41 contained 183 females grouped in pools of maximum six specimens, the remaining 127 samples belonged to individual females.

The protocol described by Montalvo *et al.* was used, with the primers F25 5'-GGACGCCGGCACGATTKCT-3' and R617 5'-GAAGAAGTCCGATACGAGGGA-3' amplifying a product of approximately 593 bp (48), *Le. (V.) braziliensis* strain WA140 was donated by PECET and was used as a positive control, and *Ny. antunesi* DNA previously negative for parasite detection was used as a negative control.

PCR reaction contained 1X buffer, 1mM $MgCl_2$, 0.2 mM dNTPs, 0.4 µM of each oligonucleotide (forward and reverse), 1 U of Taq DNA polymerase (Excel taq, SMOBIO Technology, Hsinchu Science Park, Taiwan), 1mg/ml BSA (Scientific Inc., Waltham, MA, USA), 10-40 ng/ µl of total DNA and ultrapure water until a final reaction volume of 25 µl was completed. The thermal cycling conditions consisted of an initial cycle of 1 min at 95°C, 35 cycles of 30 s at 95°C, 45 s at 61°C, 45 s at 72°C and finally a 10 min cycle at 72°C [51].

2.5 Sequence analysis PCR products

The PCR fragments were visualized with the EZ-Vision dye (Amresco, USA) on an agarose gel at 1.5 %. 100 bp plus Scientific Inc., Waltham, MA, USA) molecular weight marker were used and the images were captured through the transilluminator Essential V6 (UVITEC-CAMBRIDGE). PCR products of the expected size were subjected to DNA sequencing in both directions using ABI PRISM 3700 DNA analyzer service of Applied Biosystem.

COI, 12S rRNA, Cytb and HSP-70 N sequences were edited and aligned using the Finch TV software V 4.0 (Geospiza, Inc.) and MEGA (Molecular Evolutionary Genetics Analysis) programs respectively [52] X v11. Sequence similarity analysis was performed by comparing sequences recorded in the GenBank database with the Basic Local Alignment Tool (BLASTN) [53].

The filtered reference sequences were downloaded, and multiple alignments were constructed with the sequences from this study using ClustalW with predetermined parameters. The phylogenetic analysis was performed by constructing dendrograms with the neighbor-joining (NJ) method with genetic distances per pair and a bootstrap value

of 1000 replicates in the MEGA v11 software. Specifically in the COI marker sequences, the pair-based genetic distances for the maximum intra-specific and minimum inter-specific distances (nearest neighbor, NN) were generated using the Analysis tool with uncorrected distances (p) and the Kimura 2-parameter model (K2P) in the MEGA v11 software, while the number of haplotypes (h), polymorphic sites (s), and nucleotide diversity were determined using DnaSP 6.12.03 [54].

The graphs were made in the Linux environment. The sequence of *Nemopalpus* sp. (KM896679.1) was used as the external group for the COI dendrogram, while *Bufo montfontamus* (MK284968.1) was the external group selected for the blood intake dendrogram.

All sequences obtained in this study were submitted to GenBank with the following access codes: Cytochrome Oxidase Subunit I (COI): PQ783849 - PQ783902. Cytochrome B gene (Cytb): PQ963791 - PQ963793. Ribosomal 12S gene (12S): PQ895496 - PQ895500.

2.6 Analysis of interactions between blood-meal sources, *Leishmania* and sand fly species

The standardization of ranges in relation to the abundance of each species for each study site was calculated by the Standardized Index of Species Abundance (SISA), described by Roberts and Hsi (1979). The distribution and diversity of species was analyzed using the ANAFAU software, following the methodology described by Silveria *et al.* 1995 [55]. Through this analysis, species were classified into three categories: constant (species present in more than 50% of catches), incidental (present in 25-50% of catches) or accidental (present in less than 25% of catches). Shannon's Diversity (H) and Equity (J) indices were used to estimate species richness and abundance uniformity. The alpha diversity indices were calculated in the PAST software (V 4.10). Diversity was calculated by the Simpson 1-D index, dominance by (Dominance_D) and equity was calculated by the Shannon_H index. The distribution of species was performed by a Principal Component Analysis (PCoA) with Bray-Curtis distances.

To estimate the infection rate in positive *Leishmania* samples, the minimum infection rate is the following formula: Minimal Infection Rate (MIR) = Number of infected

phlebotomine sand flies/Total phlebotomine sand flies tested x 100 [56]. Finally, circus plot plots [57] were used to show the interactions between sand fly species, blood intake source, *Leishmania* and their detection frequencies.

3.Results

3.1 Sand fly fauna recorded in the departments of Amazonas and Caquetá

A total of 1,104 phlebotomine sand flies were collected during the entomological survey, with females comprising 68.4% (N= 755) and males 31.6% (N= 349). Of these, 191 specimens (17.3%) were collected in Amazonas and 913 (82.7%) in Caquetá. The specimens belonged to 30 species distributed across 12 genera. The most species-rich genera were *Psychodopygus* (7 species), *Nyssomyia* (4 species), *Trichophoromyia* (3 species) and *Evandromyia* Mangabeira, 1941 (3 species). The genera *Micropygomyia* Barretto, 1962 and *Brumptomyia* França & Parrot, 1921 were each represented by a single species (Table 1).

Viannamyia (Vi.) caprina (Osorno-Mesa, Morales & Osorno, 1972) (1 ♀), *Vi. tuberculata* (Mangabeira, 1941) (1 ♀) and *Sciopemyia (Sc.) fluviatilis* (Floch & Abonnenc, 1944) (3 ♀) were recorded for the first time in Amazonas, the latter representing the first record for Colombia, as far as we know.

Additionally, in Caquetá, several species were reported for the first time: *Brumptomyia (Br.) mesai* Sherlock, 1962 (1 ♂), *Ev. (Ald.) georgii* (2 ♀), *Ny. fraihai* (Martins, Falcão & Silva, 1979) (98♀, 7♂), *Ny. yuilli pajoti* (195♀, 10♂), *Lu. Tricholateralis (Trl.) sherlocki* Martins, Silva & Falcão, 1971 (1♀, 1♂), *Lu. Helcocyrtomyia (Hel.) tortura* Young & Rogers, 1984 (25♀, 15♂), *Ps. carrerai thula* (Young, 1979) (3♀, 2♂), *Trichophoromyia (Th.) howardi* (Young, 1979) (2 ♂) and *Pintomyia (Pi.) Pifanomyia (Pif.) serrana* (Damasceno & Arouck, 1949) (1♀, 1♂) (Table 1).

Table 1. Taxonomic inventory of phlebotomine sand flies, sex and feeding status of the females collected in the departments of Amazonas (Tanimboca and San Pedro de los Lagos localities) and Caquetá (Sebastopol, Jericó, Macagual, and Santo Domingo localities).

	Caquetá

	Caraño				Santo Domingo			San Martín		
		Jericó			Santo Domingo			Macagual		Tanim
ale	Female	Female B-F	Male	Female	Female B-F	Male	Female	Female B-F	Male	Female
-	-	-	1	-	-	-	-	-	-	-
-	-	2	-	-	-	-	-	-	-	-
-	-	-	-	-	-	-	-	-	-	-
-	4	9	73	-	-	1	1	-	-	-
-	-	-	-	4	1	13	101	1	-	-
-	28	4	-	40	1	7	17	-	-	-
-	-	-	-	-	-	-	-	-	-	1
-	-	2	1	17	8	8	166	1	1	-
-	1	-	-	-	-	1	-	-	-	-
-	-	-	-	25	-	15	-	-	-	-
-	-	-	-	-	-	1	-	-	-	-
2	-	-	-	-	-	-	-	-	-	1
-	-	-	-	2	1	2	-	-	-	-
-	-	-	-	3	-	2	-	-	-	-
13	-	-	-	2	-	5	-	-	-	-
-	-	-	-	5	-	-	-	-	-	-
-	-	-	1	6	-	3	-	-	-	-
-	1	-	-	-	-	-	-	-	-	-
6	-	-	-	-	-	-	-	-	-	-
-	-	-	-	-	-	1	-	-	-	-
-	-	-	-	1	-	1	-	-	-	-
-	-	-	-	1	-	-	-	-	-	-
-	-	-	-	-	-	-	-	-	-	1
-	-	-	-	-	-	-	-	-	-	-
-	112	8	116	-	-	-	-	-	-	-
-	-	-	2	-	-	-	-	-	-	-
-	-	-	-	-	-	-	-	-	-	-
-	-	-	-	-	-	-	-	-	-	-
-	-	-	-	-	-	-	-	-	-	1

-	-	-	-	-	-	-	-	-	-	-	
-	11	1	3	8	-	3	8	1	-	1	
43,75)	157 (41,32)	26 (6,84)	197 (51,84)	114 (60,64)	11 (5,85)	63 (33,51)	293 (98,65)	3 (1,01)	1 (0,34)	5 (71,43)	
		380 (41,62)			188 (20,59)			297 (32,53)			7 (3,[illegible]
				913 (82,70)							

*****New record for the department of Amazonas, ****** New record for the department of Caquetá, ° New record for Colombia, + Species of epidemiological relevance, **N (%)**: Relative abundance of captured phlebotomine sand flies, **B-F**: Fed females.

When considering the relative abundances between the two departments, *Nyssomyia* (n=449, 40.7%), *Trichophoromyia* (n=252, 22.8%), *Evandromyia* (n=136, 12.3%) and *Psychodophygus* (n=133, 12.0%) represented the most abundant genera (Fig 2), while at species level *Th. auraensis* (Mangabeira, 1942) (n=236, 21.4%), *Ny. yuilli pajoti* (n=205, 18.5 *Ev. (Ald.) walkeri* (Newstead, 1914) (n=133, 12.0%), *Ny. antunesi* (n=122, 11.0%) and *Ny. fraihai* (n=120, 10.8%) were the most abundant. Specifically in Amazon, *Ev. (Ald.) walkeri* (n=45, 23.6%) and *Ps. amazonensis* (Root, 1934) (n=26, 13.6%) were the most abundant species, while in Caquetá it was *Th. auraensis* (n=236, 25.9%), *Ny. yuilli pajoti* (n=205, 22.5%) and *Ny. antunesi* (n=120, 13.3%).

Fig 2. Relative abundances of sand fly species collected in the departments of Amazonas (Tanimboca and San Pedro de los Lagos) and Caquetá (Sebastopol, Jericó, Macagual and Santo Domingo). **N**: Total specimens by species and by genus, **%**: Percentage abundance by species and by genus.

3.2 Ecological descriptors of phlebotomine sand flies captured

The analysis of the standardized index of species abundance (SISA) by sampling area shows that *Ny. umbratilis* (SISA = 1; 28.6%) was the most abundant species associated with the Tanimboca locality, while in San Pedro de los Lagos *Ev. (Ald.) walkeri* (SISA = 0.4; 24.4%) and *Ps. amazonensis* (SISA = 0.9; 13.5) were the most abundant species. Conversely, *Ev. (Eva.) saulensis* (Floch & Abonnenc, 1944), *Lu. (Trl.) sherlocki*, *Ny. antunesi*, *Sc. fluviatilis*, *Sc. sordellii*, *Vi. Tuberculata*, and *Th. velezbernali* Posada-López, Galvis-Ovallos & Galati, 2018 were classified as rare species (Chao-1= 16.6)

associated with the department of Amazonas (Table S1). Regarding Caquetá, the most abundant species in Jericó was *Th. auraensis* (SISA= 1; 62.1%), followed by *Ev. (Ald.) walkeri* (SISA= 0.88; 22.6%). In Sebastopol, *Ps. chagasi* (Costa Lima, 1941) (SISA=0.96; 55.3%) and *Pa. Foratiiniella (For.) aragaoi* (Costa Lima, 1932) (SISA= 0.85; 20.8%) predominated.

Ny. fraihai (SISA= 0.7; 25.5%) was the most abundant in Santo Domingo, followed by *Lu. (Hel.) tortura* (SISA=1; 21.3%) while *Ny. yuilli pajoti* (SISA= 0.98; 56.5%) and *Ny. antunesi* (SISA= 0.98; 34.3%) were predominated in San Martín; *Ev. (Ald.) walkeri*, *Lu. (Trl.) sherlocki*, *Micropygomyia (Mi.) Micropygomyia (Mic.) pilosa* (Damasceno & Causey, 1944), *Ps. davisi* (Root, 1934), *Pa. (Psa.) dendrophyla* (Mangabeira, 1942) and *Ps. paraensis* (Costa Lima, 1941) were classified as rare and sporadic species (Chao-1= 20) (Table S1).

Alpha diversity indices evidenced that species richness varied considerably between locations. Santo Domingo, and San Pedro de los Lagos exhibited the highest richness with 15 and 16 species respectively, represent the localities with the greatest diversity (Simpson 1-D: 0.8235 and 0.8676), lowest dominance (Dominance_D: 0.1765 and 0.1324), and have an equitable distribution of species (Shannon_H: 2.014 2.268). Jericó and Macagual presented the highest dominance values (Dominance_D of 0.4814 and 0.4692), the lowest diversity indices (Simpson 1-D: 0.5186 and 0.5308) and the lowest evenness (Shannon_H: 0.9971 and 0.8676) (Fig S1).

The PcoA analysis with Bray-Curtis distances exhibits clear differences in species composition between localities (Fig 3). In plane I species are grouped based on ecological indices of low dominance and abundance (D: ND-D; A:C-MA). These species are similar in terms of their distribution and abundance, indicating a balance in the community. Plan II mainly represents the most abundant and dominant species. In some sites, one or more species predominated within the community, such as *Th. auraensis* in Jericó Caquetá, which could influence community dynamics, affecting resource availability and interactions among species.

Fig 3. Principal component analysis (PCoA) indicates the distribution of phlebotomine sandfly species from Bray-Curtis similarity and standardized species abundance index

(SISA). **SD**: super dominant, **D**: dominant, **ND**: non-dominant; **SA**: super abundant, **MA**: Very abundant, **C**: common, **R**: rare, **W**: Constant.

Conversely, planes III and IV include non-dominant species, characterized by their rarity and low specimen counts. Their low frequency may be associated with unfavorable environmental conditions or limited interactions with dominant species, which could contribute to their displacement.

3.3 Sequence analysis, genetic diversity, and phylogenetic information

A total of 97 sand fly sequences were obtained, representing 12 genera and 30 species from the surveyed localities in the Colombian Amazon. For most species, between three and nine specimens were included in the analysis. However, in cases of low abundance, only one or two specimens were analyzed. Additionally, 10 new COI sequences were obtained for the species *Lu. (Hel.) tortura* (6), *Trichopygomyia (Ty.) witoto* (Young & Morales, 1987) (3) and *Vi. caprina* (1).

After manually editing, the consensus sequences fluctuated from 550 to 675 bp. The fragment was aligned between positions 17 and 669 of the 5' segment of the mitochondrial COI gene of *Ae. aegypti* (OM214532.1) used as a reference genome, a region that coincides with that proposed by Hebert et al. as a barcode for species identification [58]. Visual inspection of alignment indicated the absence of termination codons in the medium of sequences suggesting the presence of pseudogenes or nuclear copies of mitochondrial origin NUMTs. The similarity percentage of between the sequences obtained when compared with the COI sequences of neotropical phlebotomine sand flies available at GenBank ranged from 93 to 100%.

During the nucleotide alignment including only the phlebotomine sand flies identified in our study, 442 preserved sites and 134 polymorphic sites were identified, of which 57 are sparingly informative. This is reflected by the nucleotide heterogeneity according to FINGERPRINT in Fig 4A. The A+T ratio (66.73%) was significantly higher than the G-C ratio (33.26%). We detected 64 haplotypes (Table S2), from 97 sequences, indicating a high degree of total nucleotide diversity per site ($\pi = 0.11596$; Standard deviation of $\pi = 0.00346$).

The number of haplotypes per species ranged from 1 to 9, the species with the greatest

haplotype diversity (Hd) were *Ps. davisi* (N= 9; Hd: 0.9778), *Ps. ayrozai* (N=5; Hd: 0.9333), *Th. auraensis* (N=5; Hd: 0.593), *Ps. chagasi* (N=4; Hd: 0.7143), and *Lu. (Hel.) tortura* (N=4; Hd: 0.8000). In these species, the percentage of nucleotide diversity ranged from 0.14 to 2.55% and 0.14 to 2.60% by the Pi and Pi (JC) models respectively (Table S2). In contrast, *Ev. (Eva.) georgii*, *Ps. paraensis* and *Ty. witoto* presented unique haplotypes from different specimens (Table S2).

The intraspecific *p* uncorrected and K2P genetic distances ranged from 0.0 % to 2.55 % (average= 0.007; standard deviation= 0.002) and 0.0 to 2.62 % (average= 0.007; standard deviation= 0.002), respectively (Table S2). The species with the highest percentages of intraspecific distance were *Pa. (For.) aragaoi* (1.19% p; 1.19% K2P), *Ps. ayrozai* (1.55% p; 1.57% K2P), *Ps. davisi* (2.55% p; 2.62% K2P), *Th. howardi* (1.50% p; 1.53% K2P), and *Ev. (Ald.) walkeri* (1.31% p; 1.33% K2P). The minimum interspecific distance (NN) for each species ranged from 0.18% to 15.45% (p distances) and 0.18% to 16.61% (K2P distances) (Table S2).

The genetic divergence analysis using the Automatic Barcode Gap Discovery ABGD method with the K2P model exhibited a marked "barcode gap" between the intra and interspecific distances of the phlebotomine sand flies collected in the Amazon, ranging from 0.25 to 0.75. This result suggests a clear separation between most of the species analyzed, as a delimiting criterion and indicator with an interspecific distance range from 0.075 to 0.175 (Fig 4B-4C). There is a slight overlap in the intraspecific distances (average 0.0042) associated with *Th. auraensis*, *Th. howardi* and *Th. velezbernali* which demonstrates limitations in the complete delimitation of these species and the need to use alternative markers with different replacement rate and inheritance. The transition/transversion rates versus genetic distance graph (Fig 4D) shows a grouping of transversions as genetic distance increases, and a pattern of substitutions that contributes to species differentiation, supporting interspecific separation.

Fig 4. Genetic divergence analysis from phlebotomine sand fly sequences collected in the departments of Amazonas and Caquetá. (A) Nucleotide heterogeneity according to FINGERPRINT, (B) ABGD “barcode gap” K2P, (C) ABGD “barcode gap” uncorrected

p distances. (D) Transitions/transversions rates versus genetic distance.

3.4 Dendrogram constructed by the Neighbor-joining method (NJ), showing the phylogenetic relationship between species divided by genera

Intra-specific taxonomic association, that is clusters of sequences of the same species and redistribution and/or rearrangement of these clusters based on genetic divergence of different genera and/or other taxonomic levels of, in this case, Amazon phlebotomine sand flies, was obtained by constructing several dendrograms of NJ using 97 COI sequences from our study and 121 reference sequences available at GenBank. For a more detailed analysis, NJ was estimated by genera.

The NJ shows a correct specific clustering supported by high Bootstrap values, between 95 and 100%, for the genus *Psychodopyguss* (Fig 5). The formation of two external clusters is evident. The first one formed by *Ps. davisi* that presented greater variability, along with the species *Ps. amazonensis*, *Ps. panamensis*, *Ps. wellcomei* Fraiha, Shaw & Lainson, 1971, *Ps. chagasi*, and *Ps. paraensis*, while the second was formed by haplotypes of *Ps. ayrozai* (Fig 5).

Fig 5. Neighbor-joining gene tree and morphology of phlebotomine sand fly COI barcode sequences, with emphasis on *Psychodopygus*. (A) NJ Dendrogram. (B-C) Male and female genitalia of *Ps. amazonensis*. (D-E) Male and female genitalia of *Ps. chagasi*. (F-G) Male and female genitalia of *Ps. paraensis*.

In *Nyssomyia*, lower Bootstrap values were also found, between 85 and 96%, and a main external cluster was formed including *Ny. antunesi*, *Ny. yuilli pajoti* and *Ny. fraihai*, while *Ny. yuilli yuilli* (Young & Porter, 1972) is shown in a different cluster (Fig 6).

Fig 6. Neighbor-joining gene tree and morphology of phlebotomine sand fly COI barcode sequences, with emphasis on *Nyssomyia*. (A) NJ Dendrogram. (B-C) Male and female genitalia of *Ny. antunesi*. (D-E) Male and female genitalia of *Ny. pajoti*. (F-G) Male and female genitalia of *Ny. fraihai*.

The NJ dendrogram that integrated *Evandromyia* and *Psathyromyia* shows Bootstrap

values of 100% for the grouping of all species (Fig 7), which demonstrates a clear consistency with the morphological allocation and low genetic variability of these species. Internally, a cluster was formed for *Evandromyia* (Bootstrap 81%) and two groups for *Psathyromyia*, one for *Pa. (For.) aragaoi* and another for *Pa. (Psa.) dendrophyla*, both with 100% support.

Fig 7. Neighbor-joining gene tree and morphology of phlebotomine sand fly COI barcode sequences, with emphasis on *Evandromyia* and *Psathyromyia*. (A) NJ Dendrogram. (B) female genitalia of *Ev. (Eva.) georgii*. (C) female genitalia of *Ev. saulensis.* (D-E) male and female genitalia of *Ev. (Ald.) walkeri*. (F) male genitalia of *Pa. (For.) aragaoi*. (G) male genitalia of *Pa. (Psa.) dendrophyla.*

The dendrogram clustering species with morphologically indistinguishable females, shows the formation of two external clusters, the first of *Trichophoromyia* and the second *Trichopygomyia* represented only by *Ty. witoto* (Fig 8). Internally, the first cluster is divided into two groups. The first comprising *Th. auraensis*, *Th. velezbernali* sequences with high genetic variability (Bootstrap of 79%) while *Th. howardi* is grouped in the second with Bootstrap values of 82% (Fig 8).

Fig 8. Neighbor-joining gene tree and morphology of phlebotomine sand fly COI barcode sequences, with emphasis on *Trichophoromyia* and *Trichopygomyia*. (A) NJ Dendrogram. (B-C) male and female genitalia of *Th. auraensis*. (D-E) male and female genitalia of *Th. howardi*. (F) male genitalia of *Ty. witoto*.

The dendrogram grouping the genera *Lutzomyia* França, 1924 and *Sciopemyia* Barretto, 1962 (Fig 9) shows three outer clusters, all with Bootstrap support values of 100%. The first cluster comprises sequences of *Lu. (Tri.) sherlocki*, the second of *Lu. (Hel.) tortura*, and the third includes the *Sciopemyia* group, which is subdivided into three well-defined clusters: the first one corresponding to *Sc. fluviatilis*, the second to *Sc. preclara*, and the third to *Sc. sordellii*, each with Bootstrap supports of 100%.

Fig 9. Neighbor-joining gene tree and morphology of phlebotomine sand

fly COI barcode sequences, with emphasis on *Lutzomyia* and *Sciopemyia*. (A) NJ Dendrogram. (B-C) male and female genitalia of *Lu. (Trl.) sherlocki*. (D-E) male and female genitalia of *Lu. (Hel.) tortura*.

The NJ dendrogram with genera of low abundances (Fig 10) clusters well-defined species with Bootstrap values of 100%, with evident low genetic variability, demonstrating clear consistency with morphological allocation. Two external clusters were formed, the first comprising sequences of *Pintomyia* Costa Lima, 1932, *Brumptomyia*, and *Micropygomyia*, while the second was formed by *Vi. furcata* and *Vi. tuberculata*

Fig 10. Neighbor-joining gene tree and morphology of phlebotomine sand fly COI barcode sequences, with emphasis on genera of lesser abundance. (A) NJ Dendrogram. (B) male genitalia of *Br. mesai*. (C) female genitalia of *Vi. caprina*. (D) female genitalia of *Vi. tuberculata*.

3.5 Identification of Feeding Sources

A total of 59 females out of the 755 collected presented visible traces of blood ingestion in the abdomen (7.81%). *Ev. (Ald.) walkeri* was the species with the highest number of females with evident blood intake (n=17; 28.81%), followed by *Ny. yuilli pajoti* (n= 11; 18.64%), *Th. auraensis* (n=8; 13.56%) and *Ny. fraihai* (n=6; 10.17%). It was possible to detect the blood source in 12 of the 13 species analyzed, and the blood source was characterized in 39 females with the Cytb molecular marker (Table S3) and 29 females (92.30%) with the marker rDNA 12S (Table S4).

After manual editing, the size of the fragment sequenced with the 12S rDNA marker ranged from 150 to 155 bp, while the length of the Cytb sequences ranged from 280 to 345 bp. The identity percentage by using the two markers was greater than 98% with a coverage of 91 to 100%, except for the *Bos (B.) taurus* (cow) and *Cheracebus (C.) lugens* (white-chested Titi), with 85.92 and 82.00% of identity, respectively (Table S3-S4).

The dendrogram generated from the Cytb marker reveals four clearly defined clusters (Bootstrap support of 100%), which highlight the genetic relationships and associated blood sources. The first cluster groups sequences (n= 17, species= 7) corresponding to *H. sapiens*, confirming its role as a frequent source of blood. The second cluster, with a

is related to *Dasyprocta (D.) leporina* (brazilian aguti) (n= 1, species= 1) and *S. scrofa* (n=10, species= 3). Finally, the third cluster, composed of *B. taurus* (n= 1, species= 1) (Fig 11).

Fig 11. Neighbor-joining gene tree and blood food sources detected in phlebotomine sand flies collected in the Colombian Amazon region, using the molecular marker Cytochrome B (Cytb). (A) Dendrogram NJ. (B) Vertebrate species identified.

The dendrogram grouping the vertebrates identified by the 12S marker reveals the formation of two main clusters, supported by Bootstrap values of 65% and 100%, respectively (Fig 12). The first cluster groups genetically close samples, such as *S. scrofa* and *H. sapiens* (n=3, species=2) with a Bootstrap value of 99%, evidencing genetic variability within each group. The second main cluster includes samples associated with *H. sapiens* (n=11, species= 7) as well as *C. lugens* (n=1, species= 1), *Saimiri (S.) macrodon* (ecuadorian squirrel monkey) (n=1, species= 1) and *F. catus* (n=1, species= 1).

Fig 12. Neighbor-joining gene tree and blood food sources detected in phlebotomine sand flies collected in the Colombian Amazon region, using the molecular marker 12S. (A) Dendrogram NJ. (B) Vertebrate species identified.

H. sapiens was the most common blood source detected in 10 of the 12 species of phlebotomine sand flies analyzed, followed by *S. scrofa* detected in *Ev. (Ald.) walkeri, Ny. fraihai*, and *Th. auraensis*. DNA of *B. taurus* and *F. catus* was also detected in the latter two. In addition, mixed feeding (*H. sapiens/D. leporina* was evidenced in a specimen of *Pi. (Pif.) nevesi* and *S. scrofa*/*C. lugens* in a specimen of *Ev. (Ald.) walkeri*, while *S. macrodon* was the only blood source detected in *Ps. davisi* (Fig 13).

Fig 13. Relationship between sources of blood ingestion and the detection of *Leishmania* DNA in phlebotomine sand flies from the department of Amazonas and Caquetá. ***Ny.a***: *Ny. antunesi*, **Ny. yp**: *Ny. yuilli pajoti*; **Ev. w**: *Ev. (Ald.) walkeri*; **Ny. fr**: *Ny. fraihai*, **Th. au**: *Th. auraensis*, **Pi. ne**: *Pi. (Pif.) nevesi*, **Sc. so:** *Sc. sordellii*, **Ps. da**: *Ps. davisi*, **Ps.pr**:

Ps. paraensis.

3.6 Detection of *Leishmania*

Leishmania DNA detection was performed in 37.9% (n=310) (Fig 14) of the total of females captured (n=816), belonging to 28 species previously identified by integrative taxonomy. Using the HSP-70 N marker, *Leishmania* DNA was amplified in 28 samples from individual females with a minimum infection rate (MIR) of 9.03% (Table S5).

Fig 14. Partial amplification of the HSP-70N gene Molecular for detections of *Leishmania* sp. in DNA total of phlebotomine sand flies collected in the Colombian Amazon region. **Ladder:** marker of 100 bp plus Scientific Inc., Waltham, MA, USA; samples **L_7, L_137** and **L_140:** DNA of females from *Ny. fraihai*; **C-** negative control; **C+**: positive control.

The species with the highest *Leishmania* detection was *Ny. fraihai* (n=8, 2,58%), collected in the peri and extra-domicile of all the localities sampled in Caquetá and in the extra-domiciliary of San Pedro de los Lagos (Table S5). All *Ny. fraihai* specimens detected with DNA from *S. scrofa* and *B. taurus* collected in Caquetá were positive as well for *Leishmania*. Additionally, the DNA of the parasite was detected in a female sand fly of the same species classified as unfed and that was collected in the peridomicile areas of Sebastopol (Fig 13).

Th. auraensis was the second species with the highest detection rate (n=4, 1,29%) (Table S5). In these sand fly specimens collected in the extradomiciliar environment of Jericó, *Leishmania* DNA was detected in females with blood traces of *H. sapiens* as well as in those without evidence of blood feeding (Fig 13). *Ny. yuilli pajoti* presented the same rate as *Th. auraensis* (n=4, 1,29%) (Table S5). It was possible to detect *Leishmania* DNA in specimens collected from Santo Domingo that were also fed with *H. sapiens* (Fig 13). *Ev. (Ald.) walkeri* presented parasite detection rates lower than 0,96% in three specimens of this sand fly collected in the extradomicile of Jericó the presence of *Leishmania* was detected, one of them with blood meal associated with *S. scrofa* (Table S5). Finally, *Leishmania* DNA was present in a single specimen of *Pi. (Pif.) nevesi* (n=1, 0,32%) collected in the extra-domiciliary environment of Santo Domingo (Table S5), which also

exhibited a mixed feeding of *D. leporina/H. sapiens* (Fig 13).

4. Discussion

This study integrates morphological, molecular and ecological approaches for the identification of phlebotomine sandflies in the Colombian Amazon, a region of high biodiversity and great vulnerability to the effects of climate change and human intervention. To the best of our knowledge, this work presents the first DNA barcoding (COI) sequences for phlebotomine sand flies from the department of Caquetá and the second study of this type in Amazonas [29]. Sixty-four new haplotypes were detected and sequences of de *Lu. (Hel.) tortura*, *Ty. witoto,* and *Vi. caprina* not previously analyzed with this marker are provided. Three new circulation records are presented for Amazonas, nine for Caquetá, and one for Colombia. Eleven species of epidemiological relevance were identified, seven vertebrate species that act as a source of blood ingestion and the presence of *Leishmania* sp. was detected in areas where cases of cutaneous leishmaniasis have been historically recorded.

Phlebotomine sand flies and epidemiological relevance

In this study, *Ev. (Ald.) walkeri* and *Ps. amazonensis* were the most abundant species in the Amazonas department, representing 36.64% of the total number of specimens. Despite limited information on these species in the region, previous studies that use a similar collection approach evidenced that *Ev. (Ald.) walkeri* does not present high dominance [59]. This can be attributed to the ecotope selected for collection, the number of traps used, the sampling effort time, and differences in bioclimatic variables recorded during collection. The department of Caquetá showed a greater richness of species, with 22 species found in the different localities, being a 18.2% higher when compared to the amount of species collected in the Amazonas department, which can be attributed to a greater sampling effort and a wider geographical range of collection in the first case. The results of species richness in this department are congruent with those recorded at Araracuara, Caquetá, during 1981 where Morales and Minter collected 35 species of phlebotomine sand flies, including six new records for the country and one new for science [31].

In Caquetá, *Th. auraensis*, *Ny. pajoti* and *Ny. antunesi* are the most abundant species, representing 61.44% of the total number of specimens caught. These results differ from previous studies in the same region, where collection during rainy seasons showed low abundance of these species [31,60]. However, studies in the Brazilian Amazon evidenced that *Th. auraensis* and *Ny. antunesi* represented approximately 40% of the fauna captured [61,62]. Notably, *Th. auraensis* has been found to be naturally infected with *Le. (V.) lainsoni* [63] and *Le. (V.) braziliensis* [63,64] in Peru and Brazil, where is considered a putative vector of *Le. Viannia* [65].

Ny. antunesi is another abundant species in Caquetá that exhibits highly anthropophilic behavior [62,66] and has been found during leishmaniasis outbreaks in the Meta department (Colombian eastern plains) with DNA of *Leishmania* not genotyped [67]. In Brazil, this species has been found infected with *L. guyanensis* [62], and is recognized as a vector of *L. infantum* and *L. braziliensis* [68]. Other phlebotomine sand flies like *Ny. yuilli pajoti*, are recognized to have the widest geographical distribution in Colombia with presence in Guaviare, Putumayo, Guainía, and Amazonas departments [20], has been registered as an anthropophilic species that is highly abundant in areas of intra, peri and extra-domiciliary in Putumayo [69], and has been collected frequently in rainy seasons and with low abundance in Amazonas and Guaviare department of Colombia [59,70].

The presence, absence, abundance, and richness of the species recorded in this study, compared with those previously reported, may differ due to changes in environmental conditions and the level habitat disturbance at the time of collection, as fluctuations in temperature, precipitation and humidity, combined with an increasing anthropogenic pressures, has favored heat waves and prolonged periods of drought, that may reduce the survival of some species, but can also accelerate immature development and promote the adaptation of others to new environments [10]. This dynamic alters vector composition and ecology, favoring their expansion in rainy seasons and their presence in previously non-endemic areas [10,71].

During this study, a new geographical distribution of various sand fly species was found. Previous records of *Vi. caprina* have been associated with its distribution in the Colombian Amazon, specifically in the Guaviare department [70] but not in the Amazonas department as found in this study. This sand fly is not classified as a vector considering that it is not involved in the transmission cycle while its distribution in the country also includes the Andean and Pacific regions [20]; *Vi. tuberculata*, vector of *Le. utingensis*, previously documented in the Amazon region of Brazil and Colombia (Caquetá and Guaviare), as well as in the Andean and Pacific regions of Colombia, in addition to the Orinoco region [20], and *Sc. fluviatilis*, whose presence was unknown in the country until now, presents a jungle-like distribution behavior, with distributions recorded in French Guiana and several states of the Brazilian Amazon [43]. Although flagellate DNA has been detected in *Sc. fluviatilis*, it has not yet been considered as a probable vector of *Leishmania* [72].

Among the nine species reported for the first time in the department of Caquetá, two represent, to our knowledge, the first record for the Colombian Amazon region, *Pi. (Pif.) serrana*, an anthropophilic species previously recorded in the Andean, Caribbean, and Orinoco regions [20], is associated with an endemic zone of *Le. (V.) brasiliensis* in Norte de Santander [73], while other species like *Ps. carrerai thula*, previously recorded in the Pacific and Andean regions of the country, is not currently associated with *Leishmania* transmission [20,74].

In addition to the species previously recorded with epidemiological relevance due to their proven or suspected role in the transmission of *Leishmania* parasites, the presence of *Ny. umbratilis*, the main vector of *L. (V.) guyanensis* in Brazil [75], is noteworthy. In Colombia, no studies have examined its role as a vector, however, it is closely associated with *L. (V.) guyanensis* in the Amazonas [76]. Currently, there are no records in Colombia of *Leishmania* infection in phlebotomine sand flies of *Ny. fraihai*, however, this species has been detected with trypanosomatids of the genus *Endotrypanum* in Porto Velho, Brazil [77]. In the case of *Lu. (Tri.) sherlocki*, captured in Caquetá and Amazonas in this study, there are records of this species in an endemic area for CL and visceral leishmaniasis (VL) in Pará, Brazil [78] and detected with *Leishmania* sp. DNA in a highly endemic area for leishmaniasis in the Peruvian Amazon [79]. Phlebotomine sand flies

like *Lu. (Hel.) tortura*, have been reported in eight municipalities in the department of Putumayo and in Puerto Nariño locality, in the Amazonas department [59,69,80]. This species is highly anthropophilic, and similar to this study, it has been captured in the peri and extradomiciliary areas [69,81]. It has not been detected with *Leishmania* DNA in the Colombian territory, but in 2013 Kato and collaborators detected high rates of natural infection by *Le.* (*V.*) *naiffi* suggesting its participation in the transmission cycle of this parasite in the Ecuadorian Amazon region [81].

The significance of finding phlebotomine sand flies like *Ps. ayrozai*, *Ps. davisi*, and *Ps. amazonensis* relies on the fact that they have been detected with *Le. naiffi* in some regions of Brazil [82]. Specifically, *Ps. ayrozai* has also been detected with DNA from *Le. amazonensis*, *Le. braziliensis*, and *Le. (V.) guyanensis* [83,84]. *Ps. panamensis*, associated with cutaneous leishmaniasis outbreaks, was recently classified as a level 4 vector [85], is highly anthropophilic, predominates in intra- and peridomiciliary areas [86], and has been detected with DNA from *Le. (V.) panamensis* [86] and *Le. (Le.) amazonensis* [87]. *Ps. chagasi* is of epidemiological relevance in Brazil and recently, DNA from *Le. braziliensis* and *Le. lainsoni* was detected in the municipality of Itapuã [83]. Although the role of *Evandromyia (Ald.) walkeri* in the transmission of leishmaniasis in Colombia has not been conclusively demonstrated, DNA from *L. braziliensis* [13] and *L. guyanensis* [62] has been detected in Acre state, Brazil.

Barcoding, a tool for species identification phlebotomine sand flies

Genetic analysis of the COI gene made it possible to differentiate species where the minimal macrometric differences of the males, associated with their parameres, require considerable expertise for their identification, as is the case with *Ny. yuilli pajoti* and *Ny. fraihai*. Improvements in the Barcode library allowed the consolidation of taxonomy within the genera *Trichophoromyia* and *Trichopygomyia*, facilitating the differentiation of species with isomorphic females, mainly *Th. auraensis*, *Th. howardi*, and *Ty. witoto*. The use of COI to differentiate species is justified by clear intraspecific (2.55%) and interspecific (16.61%) genetic distances. In some species with a considerable degree of morphological differentiation, as in the case of genera like *Nyssomyia*, *Evandromyia* and *Psychodopygus* may indicate the presence of a "barcode gap" within species evidenced

by the clear separation into two groups in the NJ.

However, when species are closely related, the analysis of genetic distances may imply an overlapping of data, preventing the recognition of a clear gap, as occurred in this study with *Th. auraensis* and *Th. velezbernali*. This is consistent with the evidence reported for species of the genus *Trichophoromyia* collected in the department of Amazonas [29]. The limitation of barcoding to discriminate sand fly species has been documented in several cases, especially when genetic distances overlap due to a possible relatively recent divergence as reported in *Ev. carmelinoi*, *Ev. evandroi* and *Ev. lenti* [88] or *Ps. complexus*, and *Ps. wellcomei* [89]. This type of integrative taxonomy studies can be complemented with the use of multilocus tools that include nuclear markers such as the Second Inward Transcribed Spacer (ITS2) or Elongation Factor alpha 1 (Ef1-α) [90], in addition to other markers such as cytochrome b (Cytb) and the 28S ribosomal RNA gene [91] that can be essential for the complete delimitation of taxa, especially for those closely related [92,93].

Hosts and Leishmania in the Amazon region

Despite the challenges posed by the disturbance of the Colombian Amazon rainforest, such as population growth, deforestation and environmental changes due to climate change, rural areas still favor the conservation of biodiversity and the richness of phlebotomines, whose wild behavior continues to shape their distribution and feeding habits. Among the vertebrate species that are considered as phlebotomine sand flies blood meal sources found in the Colombian Amazon localities analyzed, *H. sapiens* was identified as a blood source of *Ny. antunesi*, which is congruent with previous records documenting its anthropophilic behavior [16], while there are also reports of its affinity for other mammals such as *B. taurus, Choloepus didactylus* (sloth), *D. leporina*, *S. scrofa*, and *T. tetradactyla* and birds such as *Pteroglossus aracari* (toucan) [16]. *H. sapiens* was also the only blood meal source associated with *Ps. panamensis* in this study, however other investigations have found a variety of blood traces in this sand fly, including *Marmosa robinsoni* (robinson's mouse) [94] and *G. gallus* [19,94]. In addition, this species has been reported with mixed feedings of *H. sapiens*/*Equus asinus*, *H. sapiens*/*B. taurus, B. taurus/C. familiaris*, and *S. scrofa/H. sapiens* [27,95].

The anthropophilic behavior of *Ev. (Ald.) walkeri* collected in the two departments studied was evidenced by the detection of blood traces of *H. sapiens*. Traces of *C. lugens* and *S. scrofa* were also identified in this species; previously in Brazil it was found with blood traces of *G. gallus* [13] and *S. scrofa* [96], while the presence of *Leishmania* sp. in the specimens collected from Caquetá is not strange, as there are records of natural *Leishmania* sp. infection in Peru [79] and *Le. (V.) braziliensis* infection in Brazil [13]. Although information on the ecology of *Th. auraensis* is scarce, this study identified the presence of DNA from *H. sapiens*, *S. scrofa* and *F. catus*, suggesting anthropophilic and eclectic behavior. In addition, *Leishmania* sp. DNA was detected, reinforcing the connection between sources of ingestion and possible infection. Previously, *Th. auraensis* had been identified with *Le. (V.) lainsoni* [63] and *L. (V.) braziliensis* in Peru and Brazil [63,64]. In particular, the detection of DNA from *F. catus* is relevant, since this vertebrate has been sporadically reported as a host of *Le. infantum* in the Brazilian Amazon [97].

The presence of *Leishmania* in *Pi. (Pif.) nevesi* is consistent with previous studies that identified *Le. (V.) braziliensis* as the pathogen involved in the infection [13]. Although blood traces of *H. sapiens* and *G. gallus* have been detected previously in this species [13,84], this study highlights the ability of this species to feed even on different blood sources. Blood traces found in *Ny. umbratilis*, a recognized vector of *L. (V.) guyanensis* in several countries including Brazil [98], corresponded with *H. sapiens*, demonstrating its anthropophilic behavior. Although no *Leishmania* DNA was detected in this study, previously, parasites of *L. (V.) guyanensis* were isolated from females collected in the department of Amazonas [85], In addition, *Ny. umbratilis,* has been detected with DNA from *Endotrypanum* sp. [84] and with blood traces of *Plecturocebus bernhardi* and *S. scrofa* mainly in the Brazilian Amazon [96].

Ps. davisi previously detected with DNA from *L. (V.) braziliensis* [13] and *Endotrypanum* sp. [84], was not found positive with parasite DNA in this study, although its blood source preferences include *Pecari tajacu*, *S. scrofa* and *T. tetradactyla* as evidenced in other studies [96], in this research only blood traces from *H. sapiens* were found. [96]. The finding of DNA of *Leishmania* not genotyped in understudied species such as *Ny. yuilli pajoti* and *Ny. fraihai* provides valuable information on their ecology,

highlighting their anthropophilic habits and their interaction with humans in peri and extra-domiciliary areas. The selective preference of some phlebotomine sand flies species for certain hosts, such as *S. scrofa* in the Jericó locality, raises questions about their role in the dynamics of the pathogen, especially when DNA of the parasite was detected in phlebotomine sand flies associated with this vertebrate.

Conclusions

The results presented provide a solid information for future research on how the tripartite interactions between phlebotomine sand flies, hosts, and pathogens are influenced by environmental changes and ecosystem alterations in the Amazon region, including the emergence and increased abundance of opportunistic hematophagous species. Furthermore, these findings underscore the urgent need to protect remaining habitats and mitigate anthropogenic impacts that threaten to significantly alter the ecological dynamics of these areas.

In conclusion, the tripartite relationship among insects, hosts, and *Leishmania* is crucial for understanding transmission dynamics, especially in poorly studied areas of the Amazon with a high prevalence of leishmaniasis. These findings provide valuable information on zoonotic cycles in the region and reflect the impact of human intervention and habitat degradation. The identification of new blood sources suggests that some animals may be underestimated as potential reservoirs of *Leishmania*. This study reinforces the relevance of the "One Health" approach, which integrates human, animal, and environmental health, emphasizing the need to monitor phlebotomine sand flies while preserving biodiversity.

Fig 15. The ecological dynamics of the tripartite relationship among phlebotomine sand flies, blood meal sources and *Leishmania* in forest and peridomicialiary areas in the Amazon region from Colombia.

Supplementary Materials: The following supporting information can be downloaded at:

Fig S1. Alpha Diversity Indices Across Localities in the Colombian Amazon (Amazonas and Caquetá).

Table S1. Ecological estimates of abundance (standardized index), constancy, and dominance of phlebotomine sand fly species from Amazonas and Caquetá.
Table S2. Phlebotomine sand fly species, number of individuals with mitochondrial haplotypes, maximum intraspecific genetic divergence and minimum distance to nearest neighbor of the Colombian Amazonian sand fly species analyzed in this study.
Table S3. Vertebrates acting as sources of blood ingestion in phlebotomine sand flies detected with the molecular marker Ctyb in species collected in Amazonas and Caquetá, Colombian Amazon region.
Table S4. Vertebrates acting as sources of blood ingestion in phlebotomine sand flies detected with the molecular marker 12S in species collected in Amazonas and Caquetá, Colombian Amazon region.
Table S5. Prevalence of *Leishmania* infection and minimum infection rate (MIR) in phlebotomine sand flies collected in the Colombian Amazon region.

Author Contributions: Conceptualization, K.C.T., D.D.G., R.J.V.G., G.C.R., and C.X.M.H.; methodology, K.C.T., R.J.V.G., D.D.G., and C.X.M.H.; specimen identification: K.C.T; Sample processing: K.C.T.; taxonomical curatorship: R.J.V.G.; and K.C.T., software, K.C.T., and R.J.V.G., validation, K.C.T., R.J.V.G., G.C.R., and C.X.M.H.; formal analysis, K.C.T., R.J.V.G.; investigation, K.C.T., R.J.V.G.; resources, R.J.V.G., G.C.R., and C.X.M.H; data curation, K.C.T. and R.J.V.G.; writing-original draft preparation, K.C.T., and R.J.V.G.; writing—review and editing, K.C.T., D.D.G., R.J.V.G., G.C.R., and C.X.M.H; visualization, K.C.T., D.D.G., R.J.V.G., and C.X.M.H.; supervision, R.J.V.G., D.D.G., G.C.R., and C.X.M.H.; project administration, R.J.V.G., G.C.R., and C.X.M.H.; funding acquisition, C.X.M.H. All authors have read and agreed to the published version of the manuscript.

Funding: This research was funded by Universidad Nacional de Colombia, project number Hermes 57545 and the Scholarship Program of MinCiencias SGR, within the framework of Call 15: Formation of High-Level Human Capital for the Department of Caquetá through a master's degree scholarship. The APC was funded by Universidad Nacional de Colombia.

Data Availability Statement: Sequence data associated with the COI marker is available in the GenBank database as follows: PQ783849, PQ783850, PQ783851, PQ783852, PQ783853, PQ783854, PQ783855, PQ783856, PQ783857, PQ783858, PQ783859, PQ783860, PQ783861, PQ783862, PQ783863, PQ783864, PQ783865, PQ783866, PQ783867, PQ783868, PQ783869, PQ783870, PQ783871, PQ783872, PQ783873, PQ783874, PQ783875, PQ783876, PQ783877, PQ783878, PQ783879, PQ783880, PQ783881, PQ783882, PQ783883, PQ783884, PQ783885, PQ783886, PQ783887, PQ783888, PQ783889, PQ783890, PQ783891, PQ783892, PQ783893, PQ783894, PQ783895, PQ783896, PQ783897, PQ783898, PQ783899, PQ783900, PQ783901, PQ783902.
Sequence data associated with the Cytb marker is available in the GenBank database as follows: PQ963791, PQ963792, PQ963793
Sequence data associated with the 12S marker is available in the GenBank database as follows: PQ895496, PQ895497, PQ895498, PQ895499, PQ895500.

Acknowledgments: We gratefully acknowledge the communities of the different localities, to Lina Marcela Manjarrez and her team of assistants trained in community work of the Health of Florencia, Caquetá, to Dr. Luz Mila Murcia Montaño, from the Amazonas Government and the Amazonas Public Health Study Group (GESPA), to Biologist and Master's student Jennifer Danitza Viafara and Biological Engineer, to Master's student Alejandro Castañeda M, for their invaluable support during phlebotomine sand flies collections, and the access to their laboratories.

We express our special gratitude to the Program for the Programa de Estudio y Control de Enfermedades Tropicales (PECET), to its director Dr. Sara M. Robledo and Dr. Laura Cristina Posada-López for providing the space for the identification of phlebotomine sand flies, as well as for the donation of insects from the *Ae. aegypti* colony Rockefeller and *Leishmania* strains used as controls in this study.

Conflicts of Interest: The authors declare no conflicts of interest. The funders had no role in the design of the study; in the collection, analyses, or interpretation of data; in the writing of the manuscript; or in the decision to publish the results.

Bibliography

1. National institute of health (INS). Public health surveillance protocol: Leishmaniasis. Version 6. Bogotá; 2023 May. doi:doi.org/10.33610/IMYH4569
2. World Health Organization. Leishmaniasis. 2020. Available: https://www.paho.org/en/topics/leishmaniasis.
3. National institute of health (INS). Weekly epidemiological report. Bogotá; 2024 Nov. Available: https://www.ins.gov.co/buscador-eventos/Paginas/Vista-Boletin-Epidemilogico.aspx
4. National institute of health (INS). Events of interest in public health. Bogotá; 2023. Available: https://portalsivigila.ins.gov.co/Paginas/Buscador.aspx
5. Rodrigues M, Brito-Sousa JD, Dias ÁLB, Monteiro W, Sampaio V. The role of deforestation on American cutaneous leishmaniasis incidence: spatial-temporal distribution, environmental and socioeconomic factors associated in the Brazilian Amazon. Tropical Medicine & International Health. 2019;24: 348–355. doi:10.1111/tmi.13196
6. Portella T.P, Sudbrack V, Coutinho R.M, Prado P.I, Kraenkel R.A. Bayesian spatio-temporal modeling to assess the effect of land-use changes on the incidence of Cutaneous Leishmaniasis in the Brazilian Amazon. Science of The Total Environment. 2024;953: 2–7. doi:10.1016/j.scitotenv.2024.176064
7. Moreno M, Guzmán-Rodríguez L, Valderrama-Ardila C, Alexander N, Ocampo C.B. Land use in relation to composition and abundance of phlebotomines (Diptera: Psychodidae) in five foci of domiciliary transmission of cutaneous leishmaniasis in the Andean region of Colombia. Acta Trop. 2020;203: 105315. doi:10.1016/j.actatropica.2019.105315
8. Ellwanger J.H, Kulmann-Leal B, Kaminski V.L, Valverde-Villegas J.M, Da Veiga A.B, Spilki F.R, et al. Beyond diversity loss and climate change: Impacts of Amazon deforestation on infectious diseases and public health. An Acad Bras Cienc. 2020;92. doi:10.1590/0001-3765202020191375
9. Institute for Development and Peace Studies (INDEPAZ). Livestock farming and deforestation in the Amazon. Bogotá; 2024 Feb. Available: https://indepaz.org.co/ganaderia-y-deforestacion-en-la-amazonia/
10. Tidman R, Abela-Ridder B, de Castañeda RR. The impact of climate change on neglected tropical diseases: a systematic review. Trans R Soc Trop Med Hyg. 2021;115: 147–168. doi:10.1093/trstmh/traa192
11. García-Leal J, Carrero-Sarmiento D, Hoyos-López R. Diversity of the genus *Lutzomyia* (Diptera: Psychodidae) in municipalities of the department of Córdoba – Colombia. Acta Biolo Colomb. 2021;27: 394–402. doi:10.15446/abc.v27n3.90684
12. do Socorro Carvalho Miranda C, Costa de Souza B, Carvalho Garcia Miranda Filgueiras T, de Sousa AM, Cibelle da Silva Peixoto M, Carvalho Garcia Miranda Filgueiras T, et al. Visceral Leishmaniasis and Land Use and Cover in the Carajás Integration Region, Eastern Amazon, Brazil. Trop Med Infect Dis. 2022;7: 255. doi:10.3390/tropicalmed7100255
13. de Ávila M.M, Brilhante A.F, de Souza C.F, Bevilacqua P.D, Galati EAB, Brazil RP. Ecology, feeding and natural infection by *Leishmania* spp. of phlebotomine sand flies in an area of high incidence of American tegumentary leishmaniasis in the

municipality of Rio Branco, Acre, Brazil. Parasit Vectors. 2018;11: 64. doi:10.1186/s13071-018-2641-y

14. Ulloa G.M, Vásquez-Achaya F, Gomes C, del Valle LJ, Ruiz J, Pons MJ, et al. Molecular Detection of *Bartonella bacilliformis* in *Lutzomyia maranonensis* in Cajamarca, Peru: A New Potential Vector of Carrion's Disease in Peru? Am J Trop Med Hyg. 2018;99: 1229–1233. doi:10.4269/ajtmh.18-0520
15. da Silva M.S, Picelli A.M, Pereira de França K, Galati E, Andrade Filho JD, Julião GR, et al. Entomological inferences highlight the risk of *Leishmania* transmission in the urban area of Porto Velho, Rondônia, Brazil. PLoS One. 2024;19: e0309168. doi:10.1371/journal.pone.0309168
16. Pimentel AC, Sánchez Uzcátegui YDV, de Lima ACS, Silveira FT, Vasconcelos Dos Santos T, Ishikawa EAY. Blood Feeding Sources of *Nyssomyia antunesi* (Diptera: Psychodidae): A Suspected Vector of *Leishmania* (Kinetoplastida: Trypanosomatidae) in the Brazilian Amazon. J Med Entomol. 2022;59: 1847–1852. doi:10.1093/jme/tjac108
17. Roque AL, Jansen AM. Wild and synanthropic reservoirs of *Leishmania* species in the Americas. Int J Parasitol Parasites Wildl. 2014;3: 1–12. doi:10.1016/j.ijppaw.2014.08.004
18. Sandoval-Ramírez CM., Hernández C, Teherán A, Gutierrez-Marin R, Martínez-Vega R, Morales D, et al. Complex ecological interactions across a focus of cutaneous leishmaniasis in Eastern Colombia: novel description of *Leishmania* species, hosts and phlebotomine fauna. R Soc Open Sci. 2020;7: 200266. doi:10.1098/rsos.200266
19. Posada-López L, Velez-Mira A, Cantillo O, Castillo-Castañeda A, Ramírez JD, Galati E, et al. Ecological interactions of sand flies, hosts, and *Leishmania panamensis* in an endemic area of cutaneous leishmaniasis in Colombia. PLoS Negl Trop Dis. 2023;17: e0011316. doi:10.1371/journal.pntd.0011316
20. Bejarano E, Estrada L. Family Psychodidae. Zootaxa. 2016;4122: 1–53. doi:10.11646/zootaxa.4122.1.20
21. Scarpassa VM, Cunha-Machado AS, Alencar R. Multiple evolutionary lineages for the main vector of *Leishmania guyanensis*, *Lutzomyia umbratilis* (Diptera: Psychodidae), in the Brazilian Amazon. Sci Rep. 2021;11: 1–17. doi:10.1038/s41598-021-93072-4
22. Vásquez-Trujillo A, Santamaría-Herreño E, González-Reina AE, Buitrago-Álvarez LS, Góngora-Orjuela A, Cabrera-Quintero OL. *Lutzomyia antunesi* as suspected vector of cutaneous leishmaniasis in the Orinoquian region of Colombia. Rev salud pública. 2018;10 (4):625-632: 1–8.
23. Azpurua J, De La Cruz D, Valderama A, Windsor D. *Lutzomyia* Sand Fly Diversity and Rates of Infection by *Wolbachia* and an Exotic *Leishmania* Species on Barro Colorado Island, Panama. PLoS Negl Trop Dis. 2010;4: 1–9. doi:10.1371/journal.pntd.0000627
24. Molina J, Hildebrand P, Olano V, Muñoz de Hoyos P, Barreto M, Guhl F. Fauna de insectos hematófagos del sur del Parque Nacional Natural Chiribiquete, Caquetá, Colombia. Biomédica. 2000;20: 1–13. doi:10.7705/biomedica.v20i4.1075
25. Martínez Pérez L. Frecuencia de uso de animales domésticos como fuente de alimentación de *Lutzomyia* spp. (Diptera: Psychodidae) en la vereda Toro, San Cayetano, Bolívar. Universidad de Sucre 2017. 2018. Available: http://repositorio.unisucre.edu.co/handle/001/624
26. Ferro C, Marín D, Góngora R, Carrasquilla MC, Trujillo JE, Rueda NK., et al. Phlebotomine Vector Ecology in the Domestic Transmission of American Cutaneous Leishmaniasis in Chaparral, Colombia. The American Society of Tropical Medicine and Hygiene. 2011;85: 847–856. doi:10.4269/ajtmh.2011.10-0560
27. Cera-Vallejo Y, Ardila MM, Herrera L, Martínez L, Pérez-Doria A. Phlebotomine (Diptera: Psychodidae) species and their blood meal sources in a new leishmaniasis focus in Los Montes de María, Bolívar, in northern Colombia. Biomédica. 2024;44: 248–257. doi:10.7705/biomedica.6876
28. Jaimes-Dueñez J, Castillo-Castañeda A, Jiménez-Leaño Á, Duque JE, Cantillo-Barraza O, Cáceres-Rivera DI, et al. Epidemiological features of *Leishmania infantum* in dogs (*Canis lupus familiaris*) suggest a latent risk of visceral

leishmaniasis in the metropolitan area of Bucaramanga, Santander, Eastern Colombia. Prev Vet Med. 2023;219: 1–8. doi:10.1016/j.prevetmed.2023.106021

29. Posada-López L, Rodrigues B.L, Velez I.D, Uribe S. Improving the COI DNA barcoding library for Neotropical phlebotomine sand flies (Diptera: Psychodidae). Parasit Vectors. 2023;16: 1–12. doi:10.1186/s13071-023-05807-z
30. Posada-López L, Galvis-Ovallos F, Galati EAB. Description of *Trichophoromyia velezbernali*, a New Sand Fly Species (Diptera: Psychodidae: Phlebotominae) from Colombian Amazonia. J Med Entomol. 2018;55: 122–127. doi:10.1093/jme/tjx180
31. Morales A, Minter D.M. Estudio sobre flebotomíneos en Araracuara. Caquetá, Colombia, S. A. Incluyendo la descripción de *Lutzomyia araracuarensis* (Díptera, Psychodidae). Biomédica. 1981;1: 94. doi:10.7705/biomedica.v1i3.1790
32. Gutierrez MAC, Lopez ROH, Ramos AT, Vélez ID, Gomez RV, Arrivillaga-Henríquez J, et al. DNA barcoding of *Lutzomyia longipalpis* species complex (Diptera: Psychodidae), suggests the existence of 8 candidate species. Acta Trop. 2021;221: 1–9. doi:10.1016/j.actatropica.2021.105983
33. Contreras Gutiérrez MA, Vivero RJ, Vélez ID, Porter CH, Uribe S. DNA Barcoding for the Identification of Sand Fly Species (Diptera, Psychodidae, Phlebotominae) in Colombia. PLoS One. 2014;9: 1–9. doi:10.1371/journal.pone.0085496
34. Rodrigues BL, Galati EAB. Molecular taxonomy of phlebotomine sand flies (Diptera, Psychodidae) with emphasis on DNA barcoding: A review. Acta Trop. 2023;238: 1–14. doi:10.1016/j.actatropica.2022.106778
35. Hoyos L.Richard, Uribe Sandra.S, Vélez Ivan. Tipificación de especímenes colombianos de *Lutzomyia longipalpis* (Diptera: Psychodidae) mediante "Código de Barras." Rev Colomb Entomol. 2002;38-N1: 1–7.
36. CORPOAMAZONIA. Agenda ambiental departamento del Amazonas. 2008. Available: https://www.corpoamazonia.gov.co/files/Ordenamiento/agendas/01_DMarco_Agenda_Amazonas.pdf
37. Gobernación del Caquetá. Plan de Desarrollo Departamental 2020 - 2023. Caquetá; 2020 Feb. Available: https://www.caqueta.gov.co/noticias/p-lan-de-desarrollo-departamental-2020--2023
38. World Health Organization. Leishmaniasis: Number of cases of cuteneous leishmaniasis reported: 2022. 2024. Available: https://apps.who.int/neglected_diseases/ntddata/leishmaniasis/leishmaniasis.html
39. Departamento Administrativo Nacional de Estadística (DANE). Página para la descarga de datos geoestadísticos. In: Geoportal DANE [Internet]. [cited 18 Dec 2024]. Available: https://geoportal.dane.gov.co/servicios/descarga-y-metadatos/datos-geoestadisticos/
40. Departamento Administrativo Nacional de Estadística (DANE). Página para la descarga de servicios web interoperables. In: Geoportal DANE [Internet]. [cited 18 Dec 2024]. Available: https://geoportal.dane.gov.co/servicios/servicios-web-interoperables/
41. Instituto Amazónico de Investigaciones Científicas - SINCHI. Capa de Coberturas de la tierra de la Amazonia colombiana. In: Datos Abiertos - Instituto SINCHI [Internet]. 2022 [cited 18 Dec 2024]. Available: https://datos.siatac.co/search?collection=Dataset
42. QGIS Development Team. QGIS Geographic Information System. In: Open Source Geospatial Foundation Project [Internet]. 2023 [cited 18 Dec 2024]. Available: qgis.org
43. Galati EAB. Morfologia e terminologia de Phlebotominae (Diptera: Psychodidae). Classificação e identificação de táxons das Américas. Vol I. Apostila da Disciplina Bioecologia e Identificação de Phlebotominae do Programa de Pós-Graduação em Saúde Pública. . São Paulo; 2024. Available: http://www.fsp.usp.br/egalati
44. Young DG, Duncan MA. Guide to the identification and geographic distribution of *Lutzomyia* sand flies in Mexico, the West Indies, Central and South America (Diptera: Psychodidae). Gainesville. Flodda: Associated Publishers; 1994.
45. Marcondes CB. A proposal of generic and subgeneric abbreviations for phlebotomine sandflies (Diptera: Psychodidae:

Phlebotominae) of the World. Entomol News. 2007;118(4):351-356.

46. Porter CH, Collins FH. Species-Diagnostic Differences in a Ribosomal DNA Internal Transcribed Spacer from the Sibling Species *Anopheles Freeborni* and *Anopheles Hermsi* (Diptera: Culicidae). Am J Trop Med Hyg. 1991;45: 271–279. doi:10.4269/ajtmh.1991.45.271

47. Folmer O, Black M, Hoeh W, Lutz R, Vrijenhoek R. DNA primers for amplification of mitochondrial cytochrome c oxidase subunit I from diverse metazoan invertebrates. Mol Mar Biol Biotechnol. 1994;3(5), 294–299: 1–6.

48. Kitano T, Umetsu K, Tian W, Osawa M. Two universal primer sets for species identification among vertebrates. Int J Legal Med. 2007;121: 1–10. doi:10.1007/s00414-006-0113-y

49. Kocher TD, Thomas WK, Meyer A, Edwards SV, Pääbo S, Villablanca FX, et al. Dynamics of mitochondrial DNA evolution in animals: amplification and sequencing with conserved primers. Proceedings of the National Academy of Sciences. 1989;86: 1–5. doi:10.1073/pnas.86.16.6196

50. Stevens L, Dorn PL, Hobson J, de la Rua NM, Lucero DE, Klotz JH, et al. Vector Blood Meals and Chagas Disease Transmission Potential, United States. Emerg Infect Dis. 2012;18: 646–649. doi:10.3201/eid1804.111396

51. Montalvo AM, Fraga J, Maes I, Dujardin JC, Van der Auwera G. Three new sensitive and specific heat-shock protein 70 PCRs for global *Leishmania* species identification. Eur J Clin Microbiol Infect Dis. 2011;31(7):1453-61. doi:10.1007/s10096-011-1463-z

52. Kumar S, Stecher G, Li M, Knyaz C, Tamura K. MEGA X: Molecular Evolutionary Genetics Analysis across Computing Platforms. Mol Biol Evol. 2018;35: 1547–1549. doi:10.1093/molbev/msy096

53. National Center for Biotechnology Information (NCBI). Basic Local Alignment Search Tool (BLAST). [cited 19 Dec 2024]. Available: https://blast.ncbi.nlm.nih.gov/Blast.cgi

54. Rozas J, Ferrer-Mata A, Sánchez-DelBarrio JC, Guirao-Rico S, Librado P, Ramos-Onsins SE, et al. DnaSP 6: DNA Sequence Polymorphism Analysis of Large Data Sets. Mol Biol Evol. 2017;34: 3299–3302. doi:10.1093/molbev/msx248

55. Silveira Neto S, Monteiro RC, Zucchi RA, de Moraes RCB. Uso da análise faunística de insetos na avaliação do impacto ambiental. Sci Agric. 1995;52: 9–15. doi:10.1590/S0103-90161995000100003

56. VectorSurv. Pool Infection Rate. [cited 13 Jan 2025]. Available: https://vectorsurv.org/docs/tools/calculators/infection-rate/

57. Krzywinski M, Schein J, Birol I, Connors J, Gascoyne R, Horsman D, et al. Circos: An information aesthetic for comparative genomics. Genome Res. 2009;19: 1639–1645. doi:10.1101/gr.092759.109

58. Hebert PD, Ratnasingham S, deWaard JR. Barcoding animal life: cytochrome c oxidase subunit 1 divergences among closely related species. Proc R Soc Lond B Biol Sci. 2003;270. doi:10.1098/rsbl.2003.0025

59. Posada-López L. Inventario de especies y diversidad haplotípica MtCOI de Phlebotominae (Diptera: Psychodidae) en zonas de importancia para la transmisión de leishmaniasis en Colombia. Facultad de Ciencias Escuela de Biociencias, Universidad Nacional de Colombia Sede Medellín . 2016. Available: https://repositorio.unal.edu.co/handle/unal/58710

60. Osorno-Mesa E, Morales-Alarcón A, de Osorno F, Ferro-Vela C. Phlebotominae de Colombia (Diptera, Psychodidae) IX. Distribución geográfica de especies de *Brumptomyia* y *Lutzomyia* Franca 1924, encontradas en Colombia.

61. Azevedo AC, Costa SM, Pinto MC, Souza JL, Cruz HC, Vidal J, et al. Studies on the sandfly fauna (Diptera: Psychodidae: Phlebotominae) from transmission areas of American Cutaneous Leishmaniasis in state of Acre, Brazil. Mem Inst Oswaldo Cruz. 2008;103: 760–766. doi:10.1590/S0074-02762008000800003

62. Carneiro ACG, de Souza EA, Barroso EP, de Ávila MM, Melchior LAK, Rocha RDC, et al. Phlebotomine Fauna (Diptera: Psychodidae) and Infection by *Leishmania* spp. in Forest Fragments of a University Campus, Western Amazon. J Med Entomol. 2023;60: 218–223. doi:10.1093/jme/tjac162

63. Valdivia HO, De Los Santos MB, Fernandez R, Baldeviano GC, Zorrilla VO, Vera H, et al. Natural *Leishmania* Infection

of *Lutzomyia auraensis* in Madre de Dios, Peru, Detected by a Fluorescence Resonance Energy Transfer–Based Real-Time Polymerase Chain Reaction. The American Society of Tropical Medicine and Hygiene. 2012;87: 511–517. doi:10.4269/ajtmh.2012.11-0708

64. Araujo-Pereira T, Pita-Pereira D, Boité MC, Melo M, Costa-Rego TA, Fuzari AA, et al. First description of *Leishmania (Viannia)* infection in *Evandromyia saulensis*, *Pressatia* sp. and *Trichophoromyia auraensis* (Psychodidae: Phlebotominae) in a transmission area of cutaneous leishmaniasis in Acre state, Amazon Basin, Brazil. Mem Inst Oswaldo Cruz. 2017;112: 75–78. doi:10.1590/0074-02760160283

65. Teles CBG, Pessoa FAC, Medeiros JF, Camargo LMA, Araujo-Pereira T de, Pita-Pereira D de, et al. *Trichophoromyia auraensis* is a putative vector. Mem Inst Oswaldo Cruz. 2017;112: 517–519. doi:10.1590/0074-02760170024

66. Silveira FT, Ishikawa EA, De Souza AA, Lainson R. An outbreak of cutaneous leishmaniasis among soldiers in Belém, Pará State, Brazil, caused by *Leishmania (Viannia) lindenbergi* n. sp. Parasite. 2002;9: 43–50. doi:10.1051/parasite/200209143

67. Vasquez Trujillo A, Gonzalez Reina AE, Gongora Orjuela A, Prieto Suarez E, Palomares JE, Buitrago Alvarez LS. Seasonal variation and natural infection of *Lutzomyia antunesi* (Diptera: Psychodidae: Phlebotominae), an endemic species in the Orinoquia region of Colombia. Mem Inst Oswaldo Cruz. 2013;108: 463–469. doi:10.1590/S0074-0276108042013011

68. Silva ANR, Júnior AMP, de Paulo PFM, da Silva MS, Castro TS, Costa GDS, et al. Detection of *Leishmania* species (Kinetoplastida, Trypanosomatidae) in phlebotomine sand flies (Diptera, Psychodidae) from Porto Velho, Northern Brazil. Acta Trop. 2021;213: 105757. doi:10.1016/j.actatropica.2020.105757

69. Barreto M, Burbano ME, Barreto P. *Lutzomyia* sand flies (Diptera: Psychodidae) from middle and lower Putumayo department, Colombia, with new records to the country. Mem Inst Oswaldo Cruz. 2000;95: 633–637. doi:10.1590/S0074-02762000000500009

70. Cabrera OL, Mosquera L, Santamaría E. Flebótomos (Diptera: Psychodidae) del departamento de Guaviare, Colombia, con nuevos registros para el país. Biomédica. 2009;29: 73. doi:10.7705/biomedica.v29i1.43

71. González C, Paz A, Ferro C. Predicted altitudinal shifts and reduced spatial distribution of *Leishmania infantum* vector species under climate change scenarios in Colombia. Acta Trop. 2014;129: 83–90. doi:10.1016/j.actatropica.2013.08.014

72. Vasconcelos dos Santos T, Prévot G, Ginouvès M, Duarte R, Silveira FT, Póvoa MM, et al. Ecological aspects of Phlebotomines (Diptera: Psychodidae) and the transmission of American cutaneous leishmaniasis agents in an Amazonian/ Guianan bordering area. Parasit Vectors. 2018;11: 612. doi:10.1186/s13071-018-3190-0

73. Alexander B, Ferro C, Young DG, Morales A, Tesh RB. Ecology of phlebotomine sand flies (Diptera: Psychodidae) in a focus of *Leishmania (Viannia) braziliensis* in northeastern Colombia. Mem Inst Oswaldo Cruz. 1992;87: 387–395. doi:10.1590/S0074-02761992000300009

74. Contreras-Gutiérrez MA, Vélez ID, Porter C, Uribe SI. Lista actualizada de flebotomíneos (Diptera: Psychodidae: Phlebotominae) de la región cafetera colombiana. Biomédica. 2014;34. doi:10.7705/biomedica.v34i3.2121

75. Rangel EF, Lainson R. Proven and putative vectors of American cutaneous leishmaniasis in Brazil: aspects of their biology and vectorial competence. Mem Inst Oswaldo Cruz. 2009;104: 937–954. doi:10.1590/S0074-02762009000700001

76. Young D. G., Morales A, Kreutzer RD, Alexander JB, Corredor A, Tesh RB. Isolations of *Leishmania braziliensis* (Kinetoplastida: Trypanosomatidae) from Cryopreserved Colombian Sand Flies (Diptera: Psychodidae). J Med Entomol. 1987;24: 587–589. doi:10.1093/jmedent/24.5.587

77. da Silva MS, Júnior AMP, Costa NVC, Costa GDS, Rodrigues MMS, Medeiros JF. Use of light emitting diodes (LEDs) are effective and useful for sand fly ecoepidemiology studies in an Amazonian environment. Acta Trop. 2022;233: 1–7. doi:10.1016/j.actatropica.2022.106550

78. Santos Walter Souza, Ortega Fellipe Diogo, Alves Veracilda Ribeiro, Garcez Lourdes Maria. Flebotomíneos (Psychodidae: Phlebotominae) de área endêmica para leishmaniose cutânea e visceral no nordeste do estado do Pará, Brasil. Rev Panamazonica Saude. 2019;10: 1–6. doi:10.5123/S2176-6223201900059
79. Zorrilla V, De Los Santos MB, Espada L, Santos RDP, Fernandez R, Urquia A, et al. Distribution and identification of sand flies naturally infected with *Leishmania* from the Southeastern Peruvian Amazon. PLoS Negl Trop Dis. 2017;11: 1–14. doi:10.1371/journal.pntd.0006029
80. Young DG, Morales A. New Species and Records of Phlebotomine Sand Flies from Colombia (Diptera: Psychodidae). J Med Entomol. 1987;24: 662. doi:10.1093/jmedent/24.6.651
81. Kato H, Calvopiña M, Criollo H, Hashiguchi Y. First human cases of *Leishmania (Viannia) naiffi* infection in Ecuador and identification of its suspected vector species. Acta Trop. 2013;128: 710–713. doi:10.1016/j.actatropica.2013.09.001
82. Cantanhêde LM, Cupolillo E. *Leishmania (Viannia) naiffi* Lainson & Shaw 1989. Parasit Vectors. 2023;16: 194. doi:10.1186/s13071-023-05814-0
83. Resadore F, Júnior AMP, de Paulo PFM, Gil LHS, Rodrigues MMS, Araújo MDS, et al. Composition and Vertical Stratification of Phlebotomine Sand Fly Fauna and the Molecular Detection of *Leishmania* in Forested Areas in Rondônia State Municipalities, Western Amazon, Brazil. Vector-Borne and Zoonotic Diseases. 2019;19: 347–357. doi:10.1089/vbz.2018.2372
84. Araujo-Pereira T, Pita-Pereira D, Baia-Gomes SM, Boité M, Silva F, Pinto IS, et al. An overview of the sandfly fauna (Diptera: Psychodidae) followed by the detection of *Leishmania* DNA and blood meal identification in the state of Acre, Amazonian Brazil. Mem Inst Oswaldo Cruz. 2020;115. doi:10.1590/0074-02760200157
85. Posada-Lopez L, Galati EA, Shaw J, Galvis-Ovallos F. Incriminating leishmaniases vectors in Colombia: An overview and roadmap for future research. Acta Trop. 2024;260: 107409. doi:10.1016/j.actatropica.2024.107409
86. Santamaría E, Ponce N, Zipa Y, Ferro C. Presencia en el peridomicilio de vectores infectados con *Leishmania (Viannia) panamensis* en dos focos endémicos en el occidente de Boyacá, piedemonte del valle del Magdalena medio, Colombia. Biomédica. 2012;26: 82–94. doi:10.7705/biomedica.v26i1.1503
87. López M, Erazo D, Hoyos J, León C, Fuya P, Lugo L, et al. Measuring spatial co-occurrences of species potentially involved in *Leishmania* transmission cycles through a predictive and fieldwork approach. Sci Rep. 2021;11: 6789. doi:10.1038/s41598-021-85763-9
88. Soares OSR, Rodrigues BL, Shimabukuro PHF. Accessing the sand fly diversity of Tocantins, Northern Brazil: species delimitation using COI DNA barcoding. Biota Neotrop. 2024;24: 2–6. doi:10.1590/1676-0611-bn-2024-1680
89. Rodrigues BL, Baton LA, Shimabukuro PHF. Single-locus <scp>DNA</scp> barcoding and species delimitation of the sandfly subgenus *Evandromyia* (*Aldamyia*). Med Vet Entomol. 2020;34: 420–431. doi:10.1111/mve.12458
90. Di Muccio T, Marinucci M, Frusteri L, Maroli M, Pesson B, Gramiccia M. Phylogenetic analysis of Phlebotomus species belonging to the subgenus *Larroussius* (Diptera, Psychodidae) by ITS2 rDNA sequences. Insect Biochem Mol Biol. 2000;30: 387–393. doi:10.1016/S0965-1748(00)00012-6
91. Vivero RJ, Contreras-Gutiérrez MA, Bejarano EE. Changes in the carboxyl-terminal domain of cytochrome b as a taxonomic character in *Lutzomyia* (Diptera: Psychodidae). Rev Colomb Entomol. 2009;35: 83–88. doi:10.25100/socolen.v35i1.9194
92. Dupuis JR, Roe AD, Sperling FA. Multi-locus species delimitation in closely related animals and fungi: one marker is not enough. Mol Ecol. 2012;21: 4422–4436. doi:10.1111/j.1365-294X.2012.05642.x
93. Liu J, Jiang J, Song S, Tornabene L, Chabarria R, Naylor GJP, et al. Multilocus DNA barcoding – Species Identification with Multilocus Data. Sci Rep. 2017;7: 16601. doi:10.1038/s41598-017-16920-2

94. González C, León C, Paz A, López M, Molina G, Toro D, et al. Diversity patterns, *Leishmania* DNA detection, and bloodmeal identification of Phlebotominae sand flies in villages in northern Colombia. PLoS One. 2018;13: e0190686. doi:10.1371/journal.pone.0190686
95. Paternina LE, Verbel-Vergara D, Romero-Ricardo L, Pérez-Doria A, Paternina-Gómez M, Martínez L, et al. Evidence for anthropophily in five species of phlebotomine sand flies (Diptera: Psychodidae) from northern Colombia, revealed by molecular identification of bloodmeals. Acta Trop. 2016;153: 86–92. doi:10.1016/j.actatropica.2015.10.005
96. Costa GDS, Júnior AMP, Castro TS, de Paulo PFM, Ferreira GEM, Medeiros JF. Sand fly fauna and molecular detection of *Leishmania* species and blood meal sources in different rural environments in western Amazon. Acta Trop. 2021;224: 106150. doi:10.1016/j.actatropica.2021.106150
97. Dutra-Rêgo F, Silva MSD, Isnard AP, Medeiros JF, Andrade Filho JD, Freire ML. You are what you eat: a systematic review exploring the interaction between Brazilian sand flies and their vertebrate food sources. Mem Inst Oswaldo Cruz. 2024;119. doi:10.1590/0074-02760240055
98. Ready PD, Arias JR, Freitas RA. A pilot study to control *Lutzomyia umbratilis* (Diptera: Psychodidae), the major vector of *Leishmania braziliensis guyanensis*, in a peri-urban rainforest of Manaus, Amazonas State, Brazil. Mem Inst Oswaldo Cruz. 1985;80: 27–36. doi:10.1590/S0074-02761985000100005

Abstract_graphic

Click here to access/download;Figure;Abstract_graphic_PNTD-D-25-00172.tif

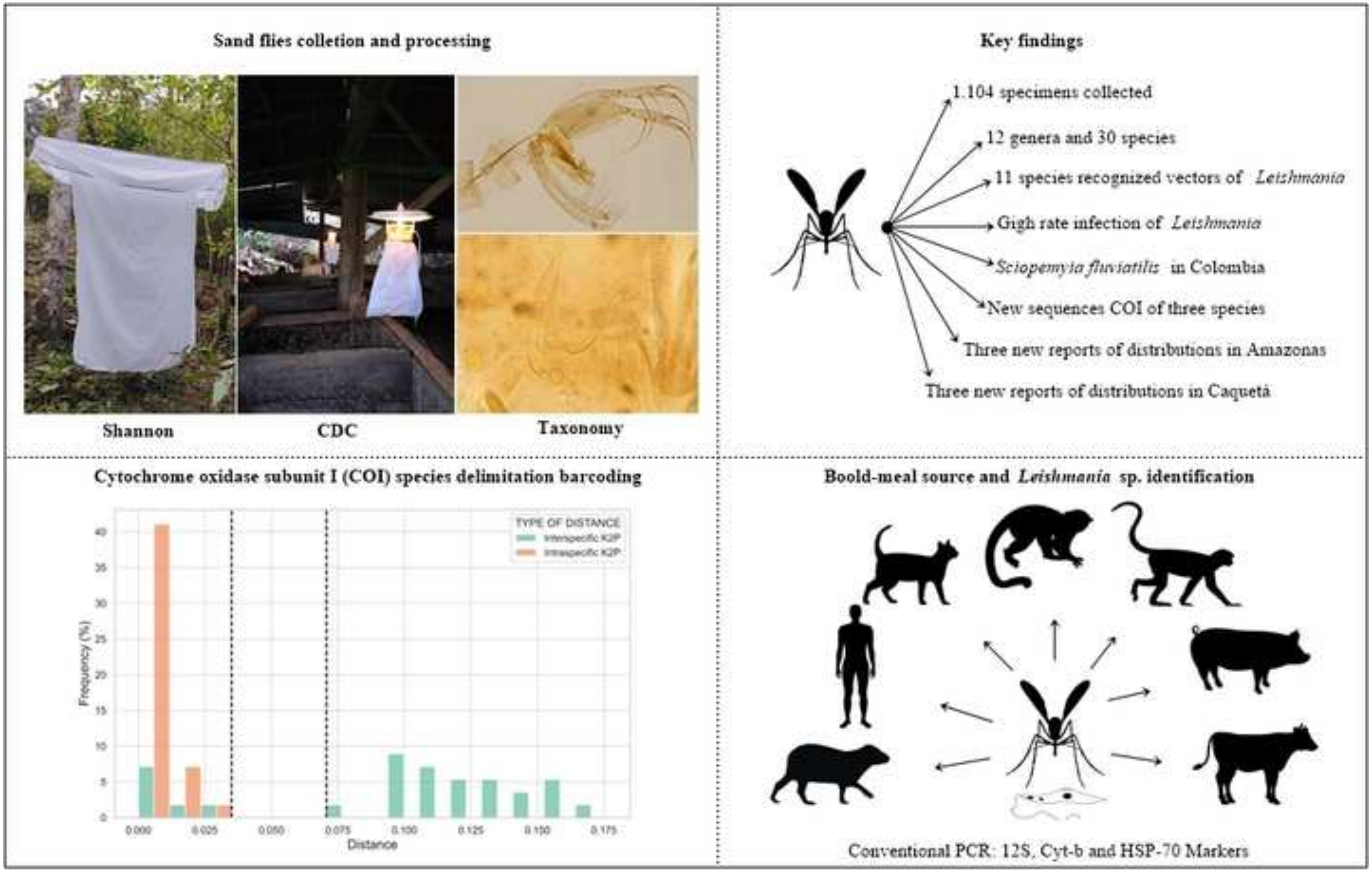

Click here to access/download;Figure;Fig_1.docx

Fig 1. Geographic location of the study area and phlebotomine sand fly collection sites: (A) Departments of Amazonas and Caquetá, Colombia, South America. (B) Municipality of Florencia, locality Jericó, Macagual, Santo Domingo and Sebastopol. (C) Municipality of Leticia, locality Leticia, San Pedro de los Lagos and Tanimboca. Source of layers [39–41] and Qgis, v. 3.34.12 [42].

Figure 1

Click here to access/download;Figure;Fig_1.tif

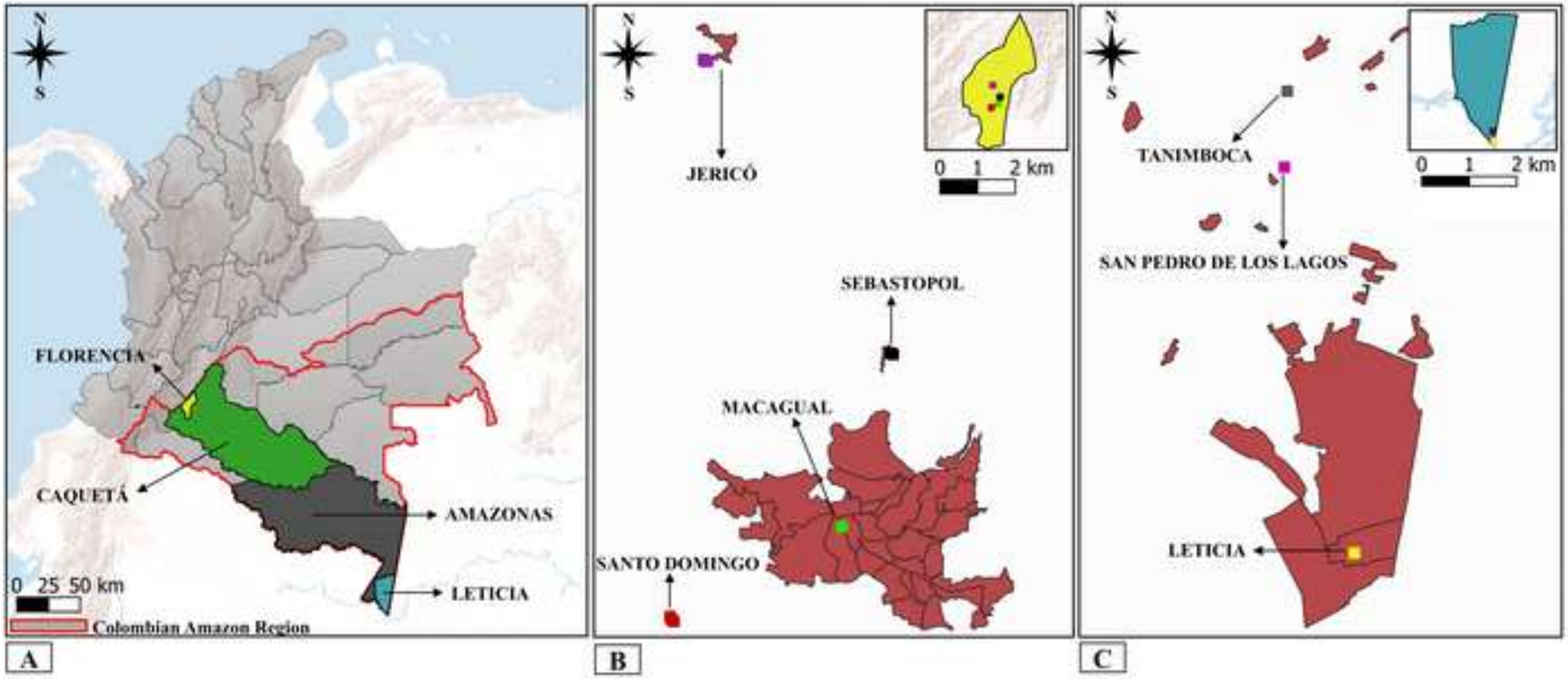

Legend Figure 2 Click here to access/download;Figure;Fig_2.docx

Fig 2. Relative abundances of sand fly species collected in the departments of Amazonas (Tanimboca and San Pedro de los Lagos) and Caquetá (Sebastopol, Jericó, Macagual and Santo Domingo). **N:** Total specimens by species and by genus, **%**: Percentage abundance by species and by genus.

Figure 2

Click here to access/download;Figure;Fig_2.tif

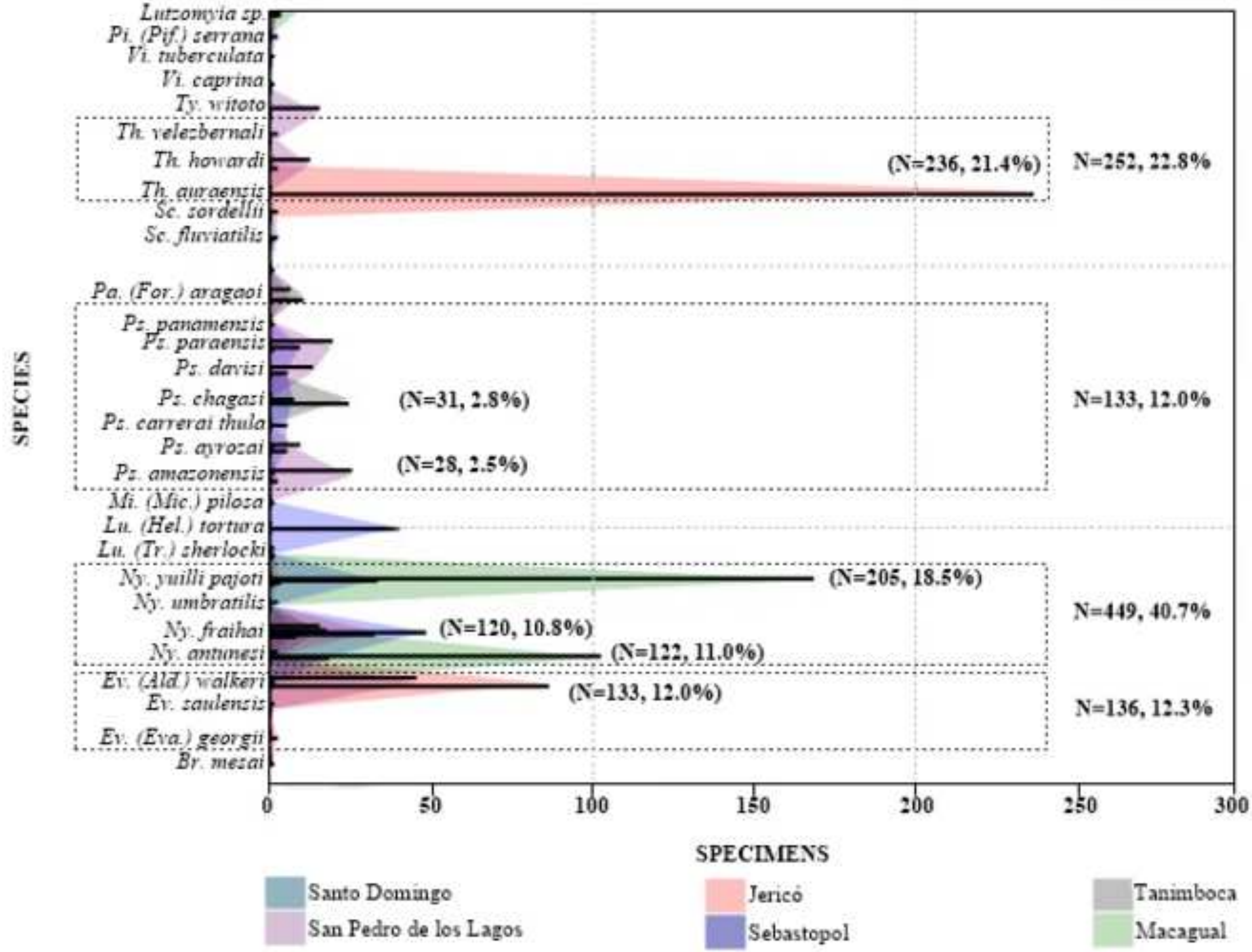

Legend Figure 3 Click here to access/download;Figure;Fig _3.docx

Fig 3. Principal component analysis (PCoA) indicates the distribution of phlebotomine sandfly species from Bray-Curtis similarity and standardized species abundance index (SISA). **SD**: super dominant, **D**: dominant, **ND**: non-dominant; **SA**: super abundant, **MA**: Very abundant, **C**: common, **R**: rare, **W**: Constant.

Figure 3

Click here to access/download;Figure;Fig_3.tif

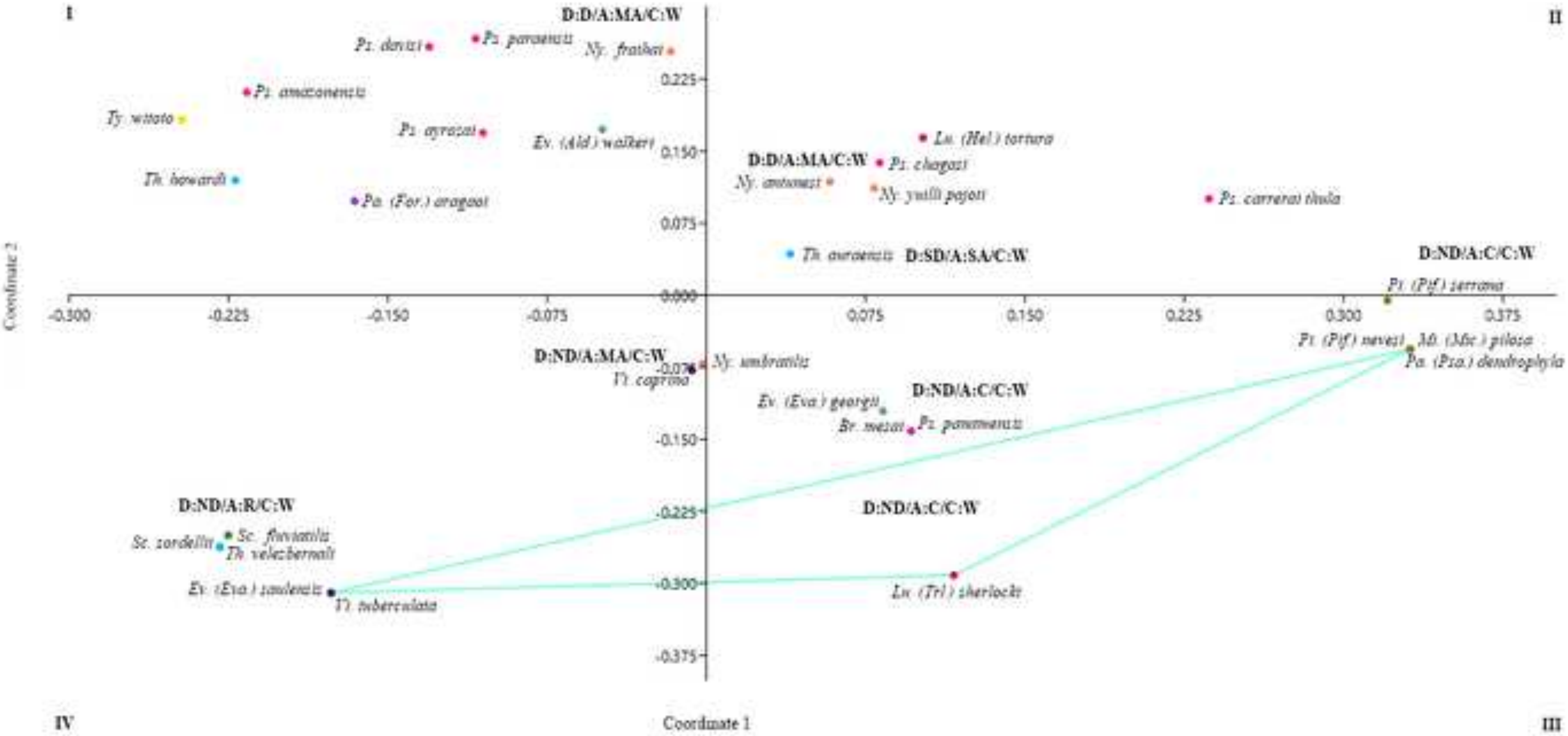

Click here to access/download;Figure;Fig_4.docx

Fig 4. Genetic divergence analysis from phlebotomine sand fly sequences collected in the departments of Amazonas and Caquetá. (A) Nucleotide heterogeneity according to FINGERPRINT, (B) ABGD "barcode gap" K2P, (C) ABGD "barcode gap" uncorrected p distances. (D) Transitions/transversions rates versus genetic distance.

Figure 4

Click here to access/download;Figure;Fig_4.tif

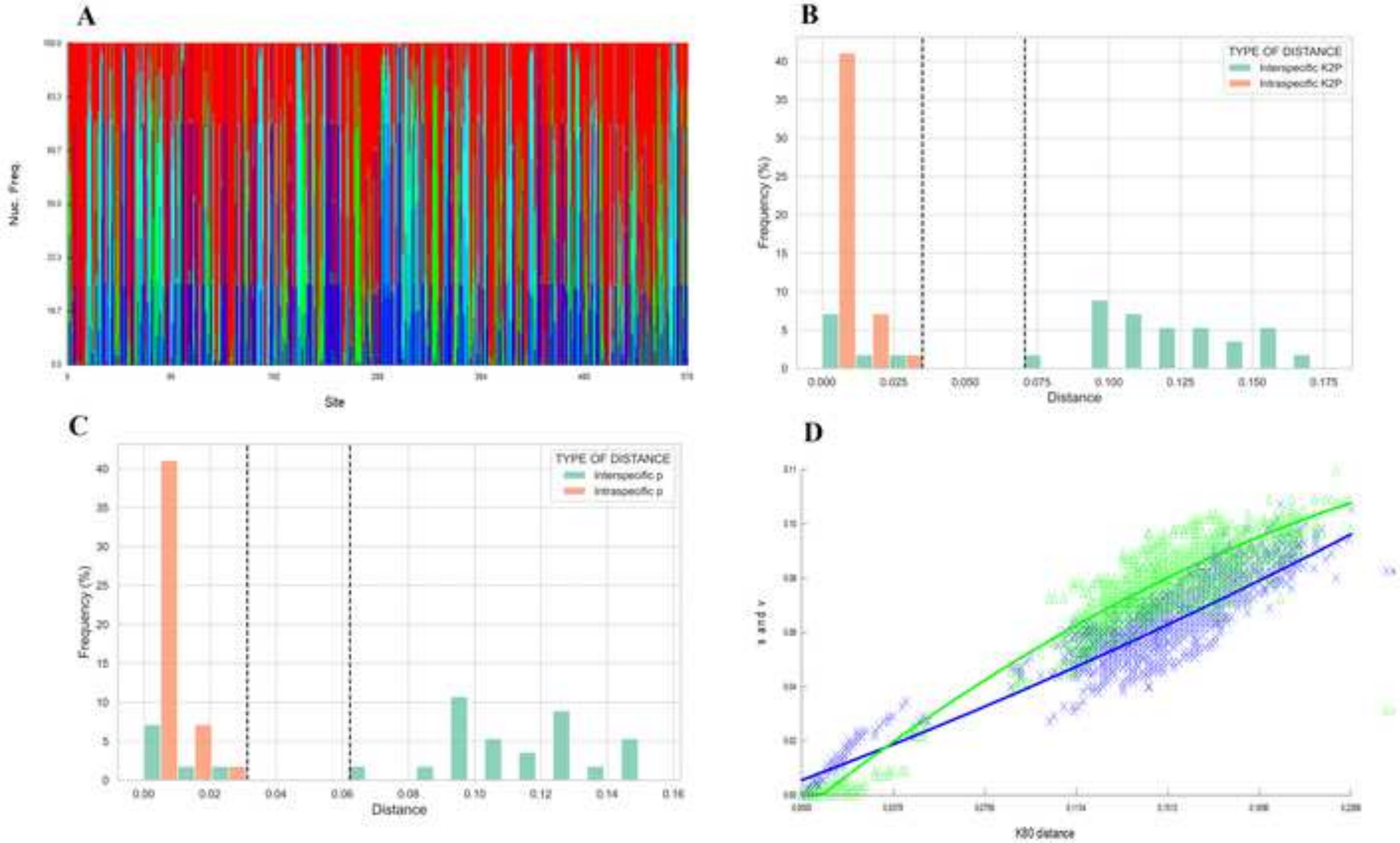

Legend Figure 5 Click here to access/download;Figure;Fig_5.docx

Fig 5. Neighbor-joining gene tree and morphology of phlebotomine sand fly COI barcode sequences, with emphasis on *Psychodopygus*. (A) NJ Dendrogram. (B-C) Male and female genitalia of *Ps. amazonensis*. (D-E) Male and female genitalia of *Ps. chagasi*. (F-G) Male and female genitalia of *Ps. paraensis*.

Figure 5

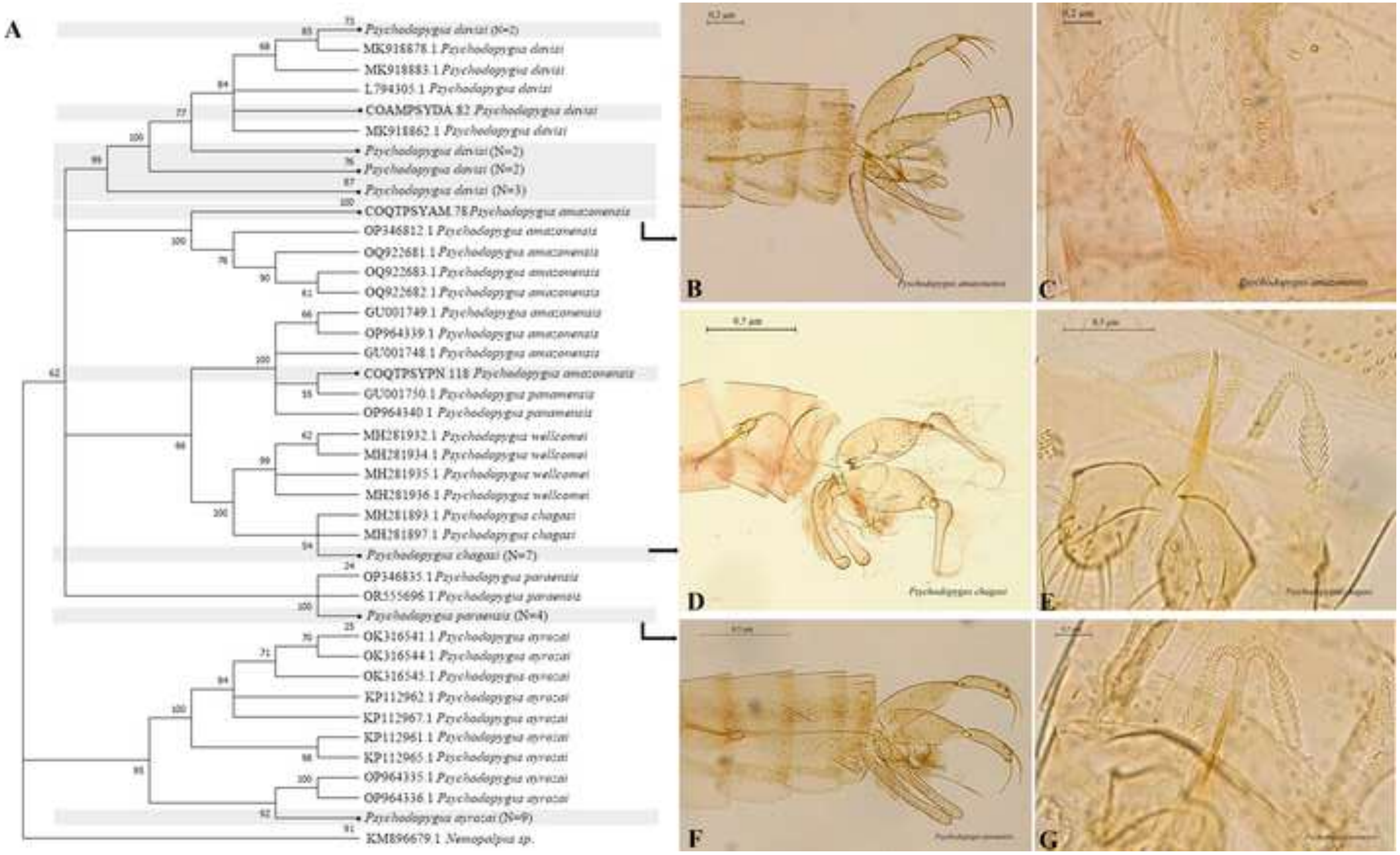
A
Psychodopygus davisi (N=2)
MK918878.1 Psychodopygus davisi
MK918883.1 Psychodopygus davisi
L794305.1 Psychodopygus davisi
COAMPSYDA 82 Psychodopygus davisi
MK918862.1 Psychodopygus davisi
Psychodopygus davisi (N=2)
Psychodopygus davisi (N=2)
Psychodopygus davisi (N=3)
COQTPSYAM 78 Psychodopygus amazonensis
OP346812.1 Psychodopygus amazonensis
OQ922681.1 Psychodopygus amazonensis
OQ922683.1 Psychodopygus amazonensis
OQ922682.1 Psychodopygus amazonensis
GU001749.1 Psychodopygus amazonensis
OP964339.1 Psychodopygus amazonensis
GU001748.1 Psychodopygus amazonensis
COQTPSYPN 118 Psychodopygus amazonensis
GU001750.1 Psychodopygus panamensis
OP964340.1 Psychodopygus panamensis
MH281932.1 Psychodopygus wellcomei
MH281934.1 Psychodopygus wellcomei
MH281935.1 Psychodopygus wellcomei
MH281936.1 Psychodopygus wellcomei
MH281893.1 Psychodopygus chagasi
MH281897.1 Psychodopygus chagasi
Psychodopygus chagasi (N=7)
OP346835.1 Psychodopygus paraensis
OR555696.1 Psychodopygus paraensis
Psychodopygus paraensis (N=4)
OK316541.1 Psychodopygus ayrozai
OK316544.1 Psychodopygus ayrozai
OK316545.1 Psychodopygus ayrozai
KP112962.1 Psychodopygus ayrozai
KP112967.1 Psychodopygus ayrozai
KP112961.1 Psychodopygus ayrozai
KP112965.1 Psychodopygus ayrozai
OP964335.1 Psychodopygus ayrozai
OP964336.1 Psychodopygus ayrozai
Psychodopygus ayrozai (N=9)
KM896679.1 Nemopalpus sp.
B
C
D
E
F
G

Click here to access/download;Figure;Fig_6.docx

Fig 6. Neighbor-joining gene tree and morphology of phlebotomine sand fly COI barcode sequences, with emphasis on *Nyssomyia*. (A) NJ Dendrogram. (B-C) Male and female genitalia of *Ny*. *antunesi*. (D-E) Male and female genitalia of *Ny*. *pajoti*. (F-G) Male and female genitalia of *Ny*. *fraihai*.

Figure 6

Click here to access/download;Figure;Fig_6.tif

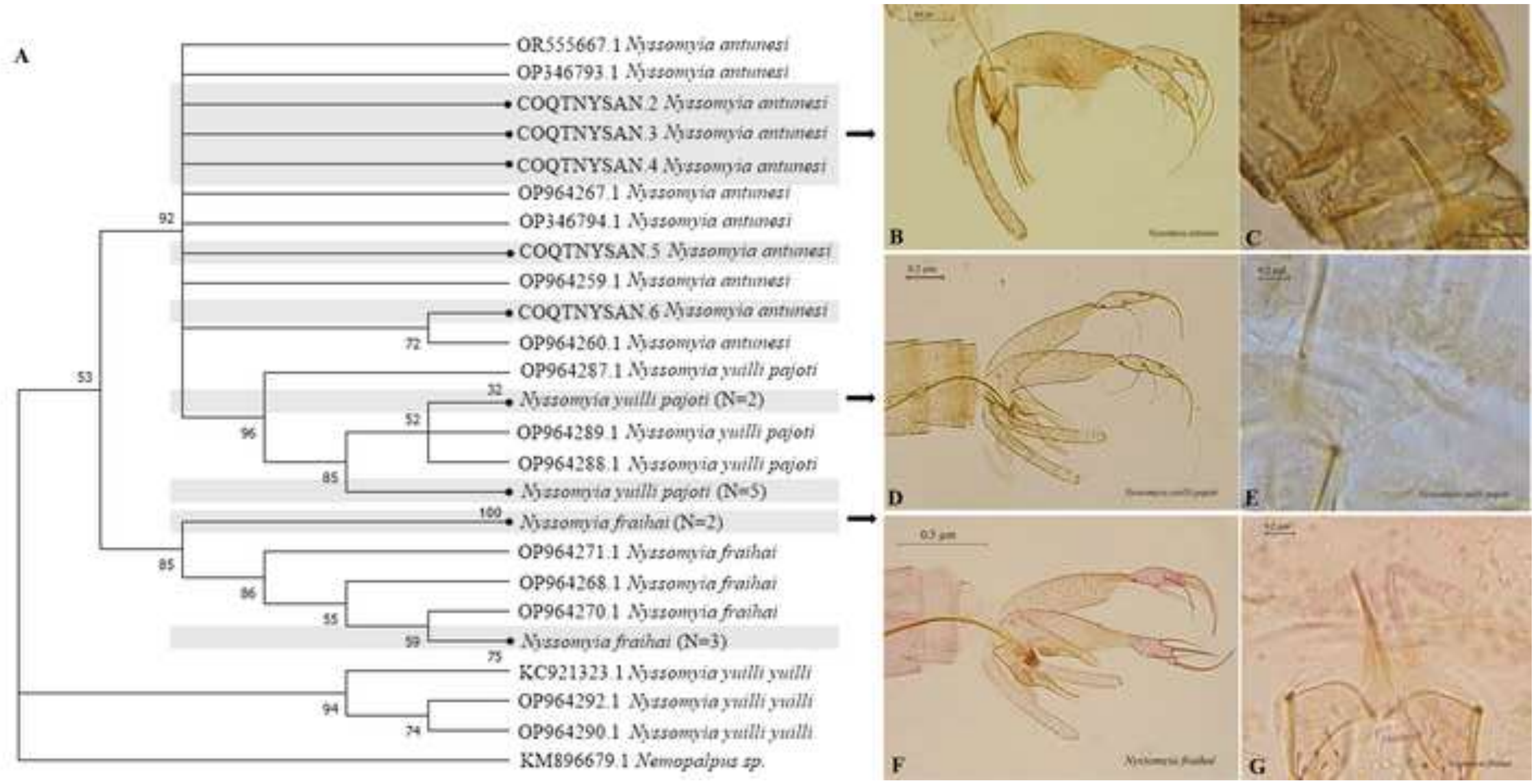

Click here to access/download;Figure;Fig_7.docx

Fig 7. Neighbor-joining gene tree and morphology of phlebotomine sand fly COI barcode sequences, with emphasis on *Evandromyia* and *Psathyromyia*. (A) NJ Dendrogram. (B) female genitalia of *Ev. (Eva.) georgii*. (C) female genitalia of *Ev. saulensis.* (D-E) male and female genitalia of *Ev. (Ald.) walkeri*. (F) male genitalia of *Pa. (For.) aragaoi*. (G) male genitalia of *Pa. (Psa.) dendrophyla.*

Figure 7 Click here to access/download;Figure;Fig_7.tif

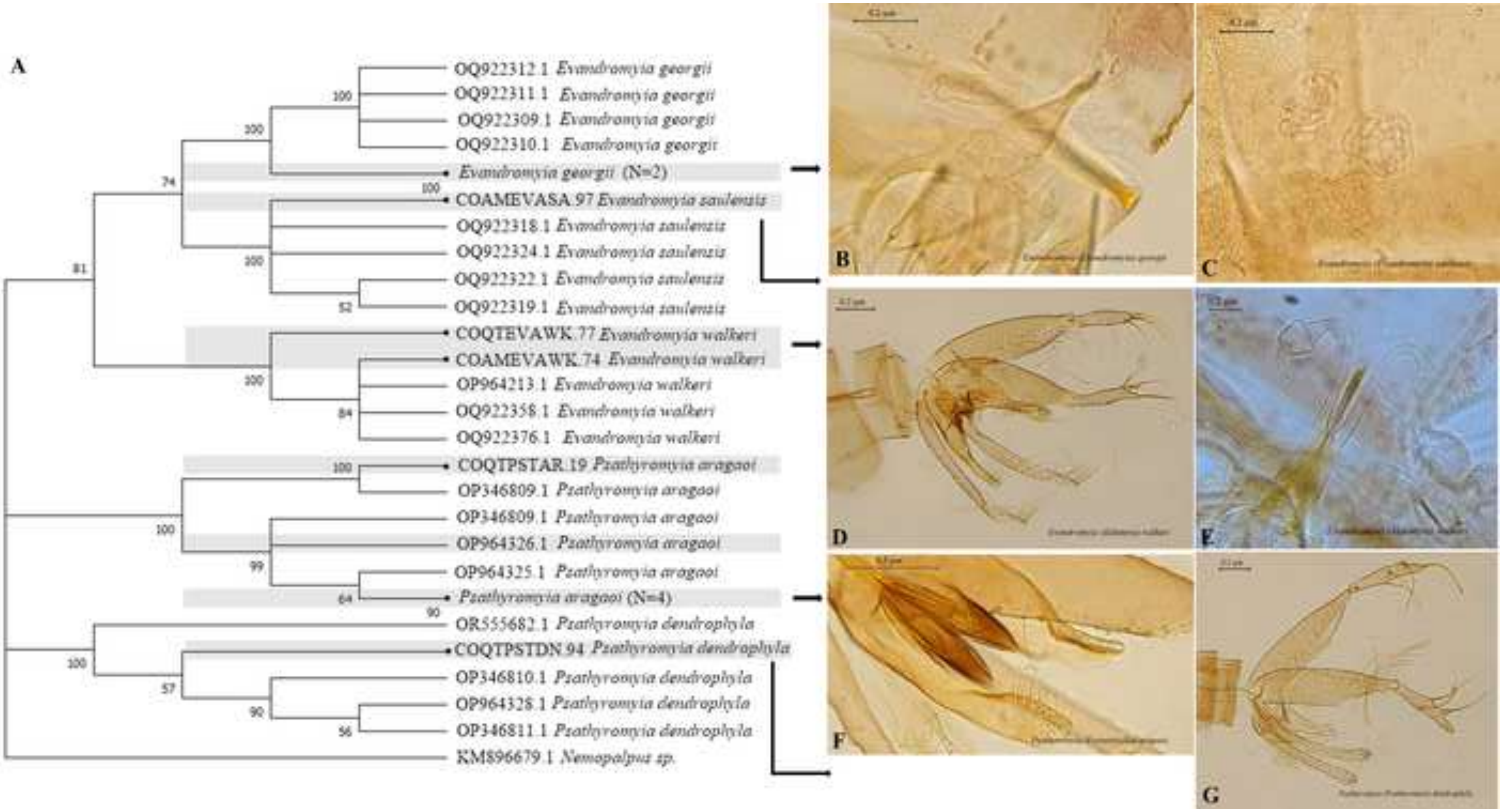

Legend Figure 8 Click here to access/download;Figure;Fig_8.docx

Fig 8. Neighbor-joining gene tree and morphology of phlebotomine sand fly COI barcode sequences, with emphasis on *Trichophoromyia* and *Trichopygomyia*. (A) NJ Dendrogram. (B-C) male and female genitalia of *Th. auraensis*. (D-E) male and female genitalia of *Th. howardi*. (F) male genitalia of *Ty. witoto*.

Figure 8

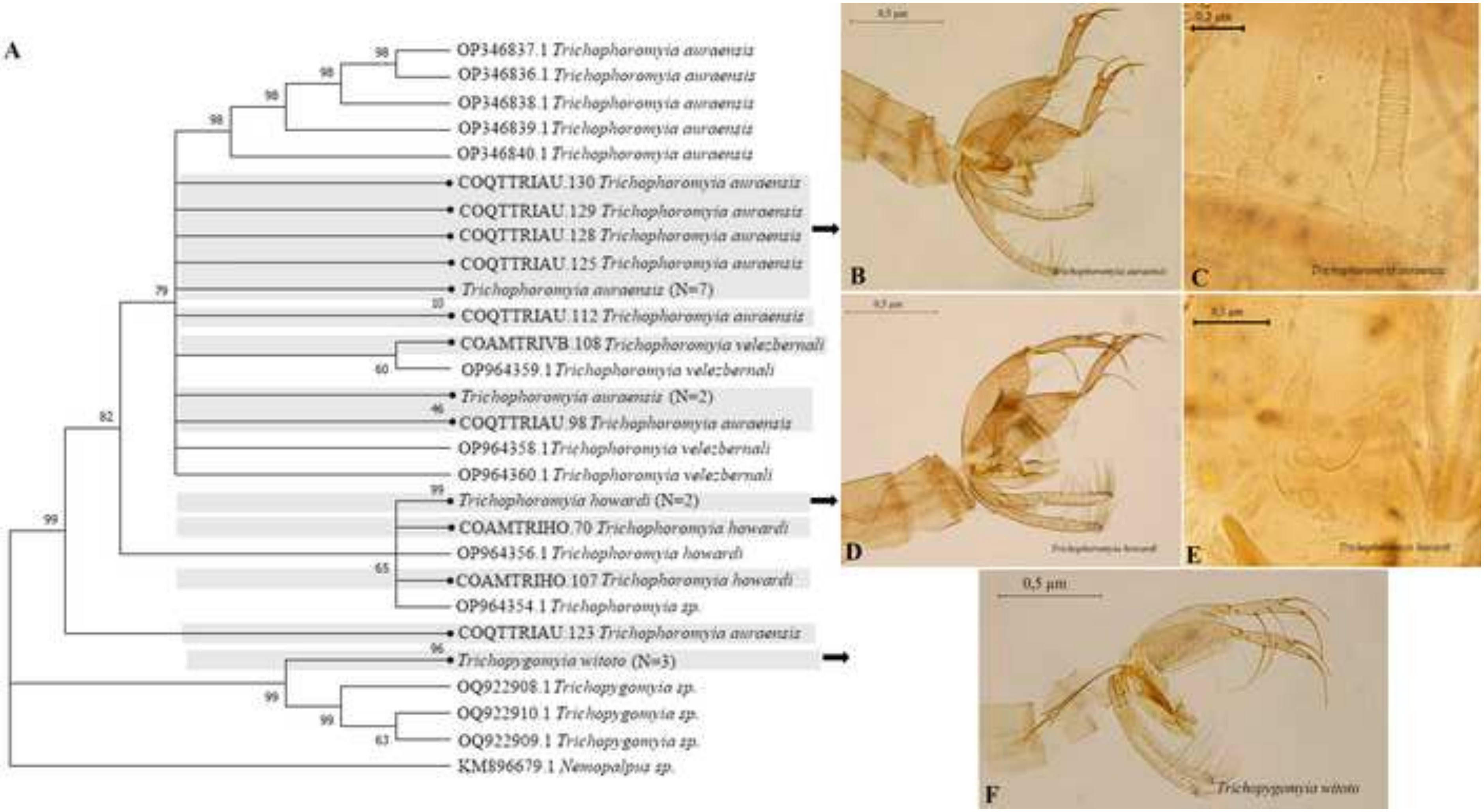
A
OP346837.1 Trichophoromyia auraensis
OP346836.1 Trichophoromyia auraensis
OP346838.1 Trichophoromyia auraensis
OP346839.1 Trichophoromyia auraensis
OP346840.1 Trichophoromyia auraensis
COQTTRIAU.130 Trichophoromyia auraensis
COQTTRIAU.129 Trichophoromyia auraensis
COQTTRIAU.128 Trichophoromyia auraensis
COQTTRIAU.125 Trichophoromyia auraensis
Trichophoromyia auraensis (N=7)
COQTTRIAU.112 Trichophoromyia auraensis
COAMTRIVB.108 Trichophoromyia velezbernali
OP964359.1 Trichophoromyia velezbernali
Trichophoromyia auraensis (N=2)
COQTTRIAU.98 Trichophoromyia auraensis
OP964358.1 Trichophoromyia velezbernali
OP964360.1 Trichophoromyia velezbernali
Trichophoromyia howardi (N=2)
COAMTRIHO.70 Trichophoromyia howardi
OP964356.1 Trichophoromyia howardi
COAMTRIHO.107 Trichophoromyia howardi
OP964354.1 Trichophoromyia sp.
COQTTRIAU.123 Trichophoromyia auraensis
Trichopygomyia witoto (N=3)
OQ922908.1 Trichopygomyia sp.
OQ922910.1 Trichopygomyia sp.
OQ922909.1 Trichopygomyia sp.
KM896679.1 Nemopalpus sp.
98
98
98
98
79
82
99
60
46
99
65
96
99
99
63
B
C
D
E
0,5 µm
Trichopygomyia witoto
F

Click here to access/download;Figure;Fig_9.docx

Fig 9. Neighbor-joining gene tree and morphology of phlebotomine sand fly COI barcode sequences, with emphasis on *Lutzomyia* and *Sciopemyia*. (A) NJ Dendrogram. (B-C) male and female genitalia of *Lu. (Trl.) sherlocki*. (D-E) male and female genitalia of *Lu. (Hel.) tortura*.

Figure 9

Click here to access/download;Figure;Fig_9.tif

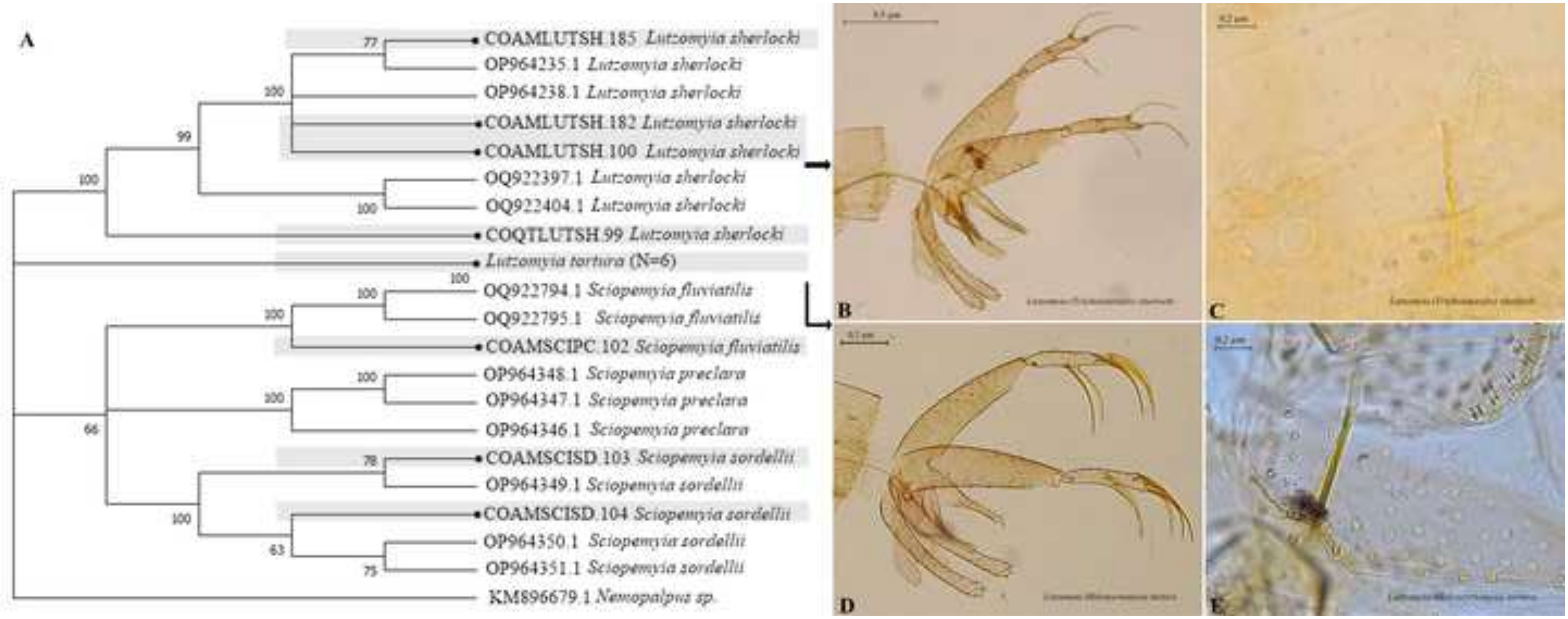

 Click here to access/download;Figure;Fig_10.docx

Fig 10. Neighbor-joining gene tree and morphology of phlebotomine sand fly COI barcode sequences, with emphasis on genera of lesser abundance. (A) NJ Dendrogram. (B) male genitalia of *Br. mesai*. (C) female genitalia of *Vi. caprina*. (D) female genitalia of *Vi. tuberculata*.

.

Figure 10

Click here to access/download;Figure;Fig_10.tif

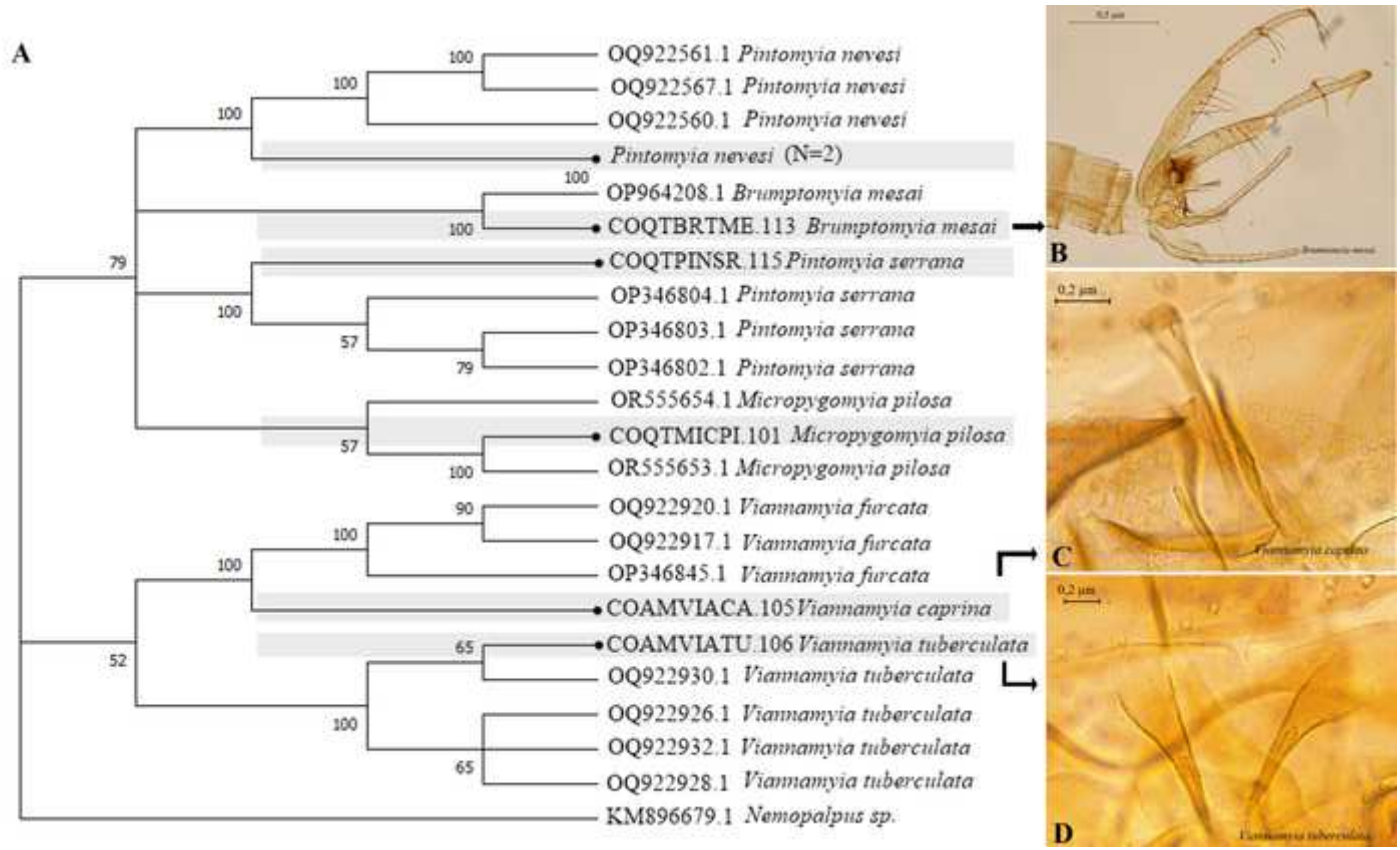

Click here to access/download;Figure;Fig_11.docx

Fig 11. Neighbor-joining gene tree and blood food sources detected in phlebotomine sand flies collected in the Colombian Amazon region, using the molecular marker Cytochrome B (Cytb). (A) Dendrogram NJ. (B) Vertebrate species identified.

Figure 11 Click here to access/download;Figure;Fig_11.tif

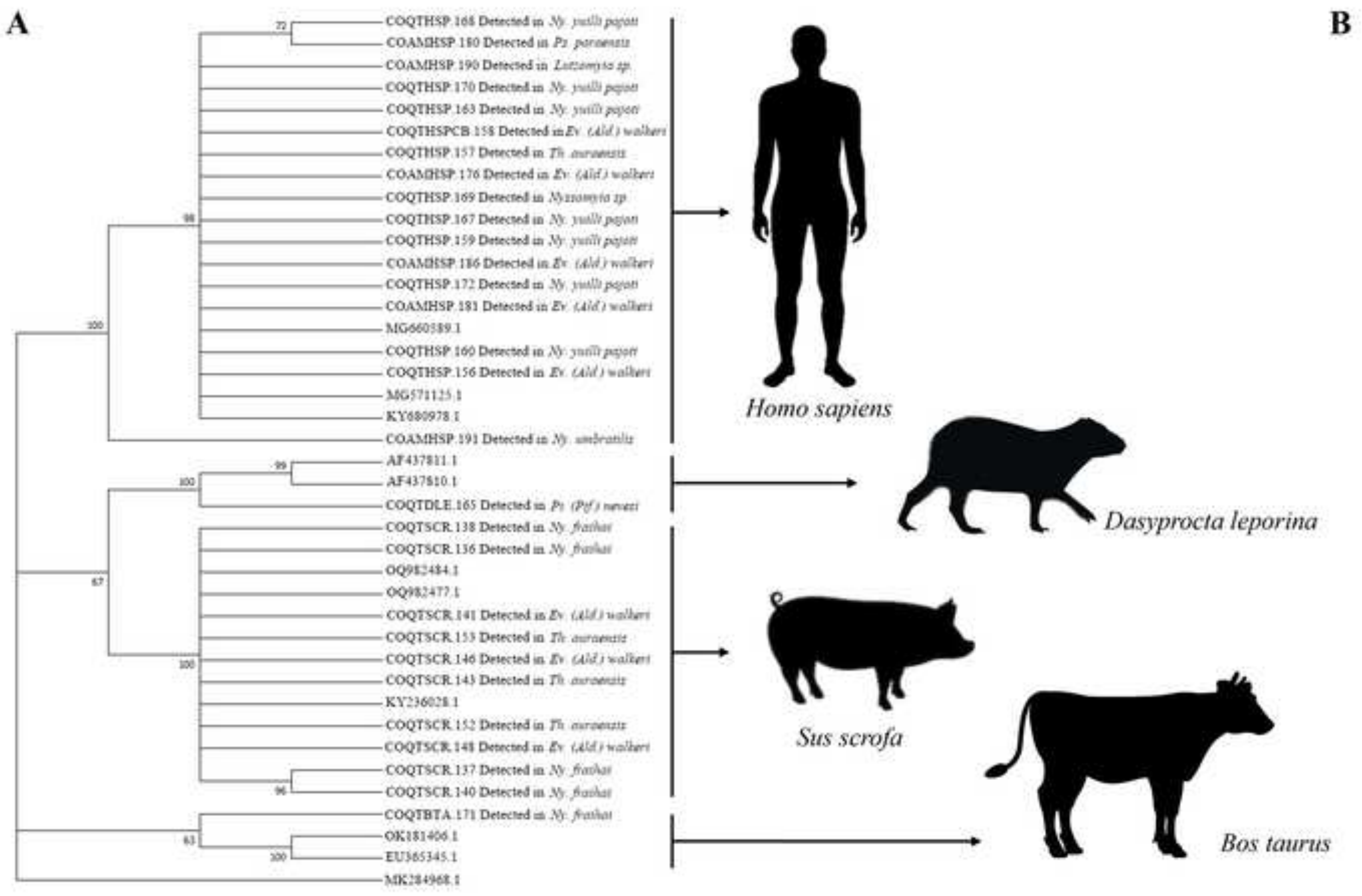

Legend Figure 12 Click here to access/download;Figure;Fig_12.docx

Fig 12. Neighbor-joining gene tree and blood food sources detected in phlebotomine sand flies collected in the Colombian Amazon region, using the molecular marker 12S. (A) Dendrogram NJ. (B) Vertebrate species identified.

Figure 12

Click here to access/download;Figure;Fig_12.tif

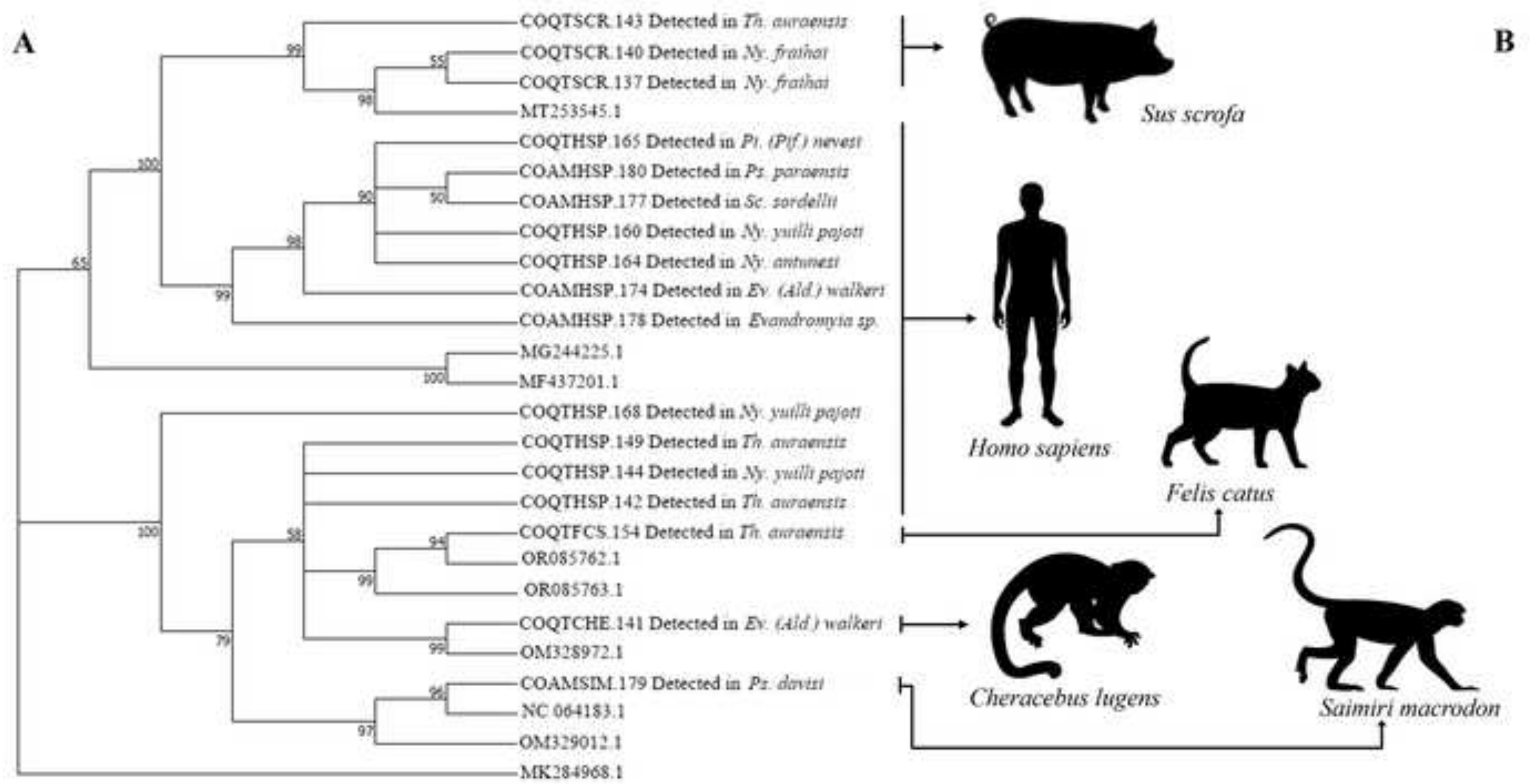

Legend Figure 13 Click here to access/download;Figure;Fig_13.docx

Fig 13. Relationship between sources of blood ingestion and the detection of *Leishmania* DNA in phlebotomine sand flies from the department of Amazonas and Caquetá. ***Ny.a***: *Ny. antunesi*, ***Ny. yp***: *Ny. yuilli pajoti*; ***Ev. w***: *Ev. (Ald.) walkeri*; ***Ny. fr***: *Ny. fraihai*, ***Th. au***: *Th. auraensis*, ***Pi. ne***: *Pi. (Pif.) nevesi*, **Sc. so:** *Sc. sordellii*, ***Ps. da***: *Ps. davisi*, ***Ps.pr***: *Ps. paraensis*.

Figure 13

Click here to access/download;Figure;Fig_13.tif

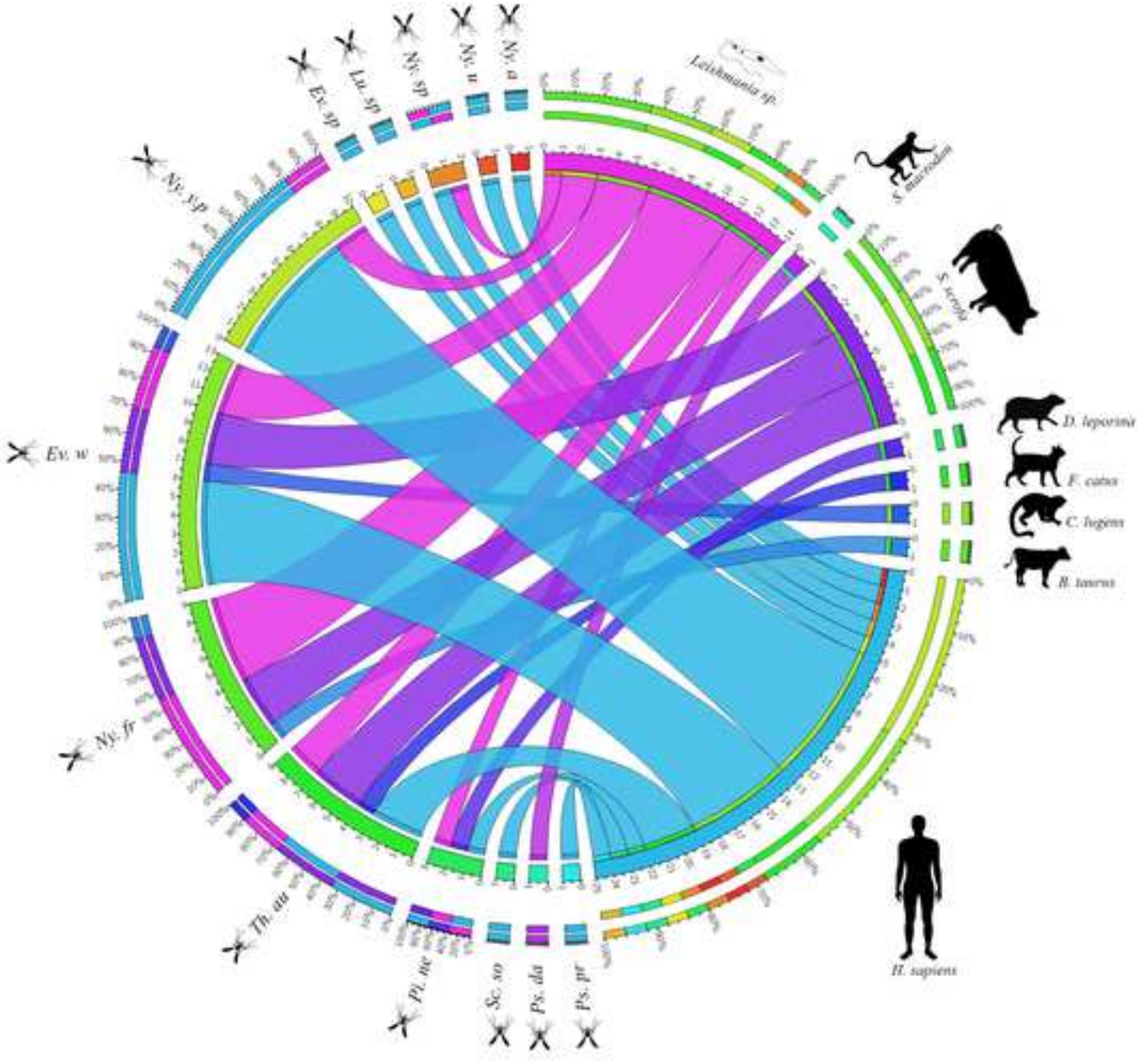

Click here to access/download;Figure;Fig_14.docx

Fig 14. Partial amplification of the HSP-70N gene Molecular for detections of *Leishmania* sp. in DNA total of phlebotomine sand flies collected in the Colombian Amazon region. **Ladder:** marker of 100 bp plus Scientific Inc., Waltham, MA, USA; samples **L_7, L_137** and **L_140:** DNA of females from *Ny. fraihai*; **C-** negative control; **C+**: positive control.

Figure 14

Click here to access/download;Figure;Fig_14.tif

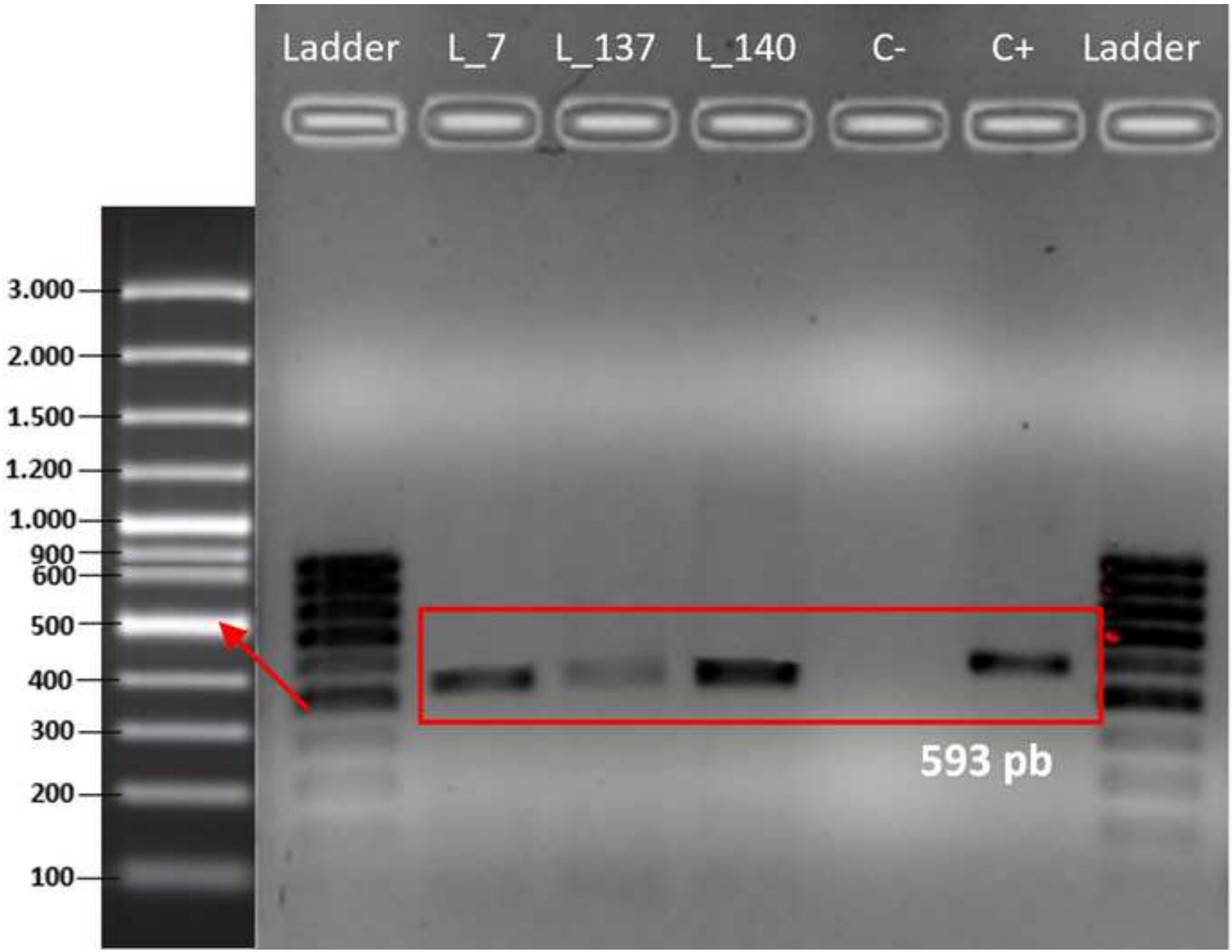

Click here to access/download;Figure;Fig_15.docx

Fig 15. The ecological dynamics of the tripartite relationship among phlebotomine sand flies, blood meal sources and *Leishmania* in forest and peridomicialiary areas in the Amazon region from Colombia.

Figure 15

Click here to access/download;Figure;Fig_15.tif

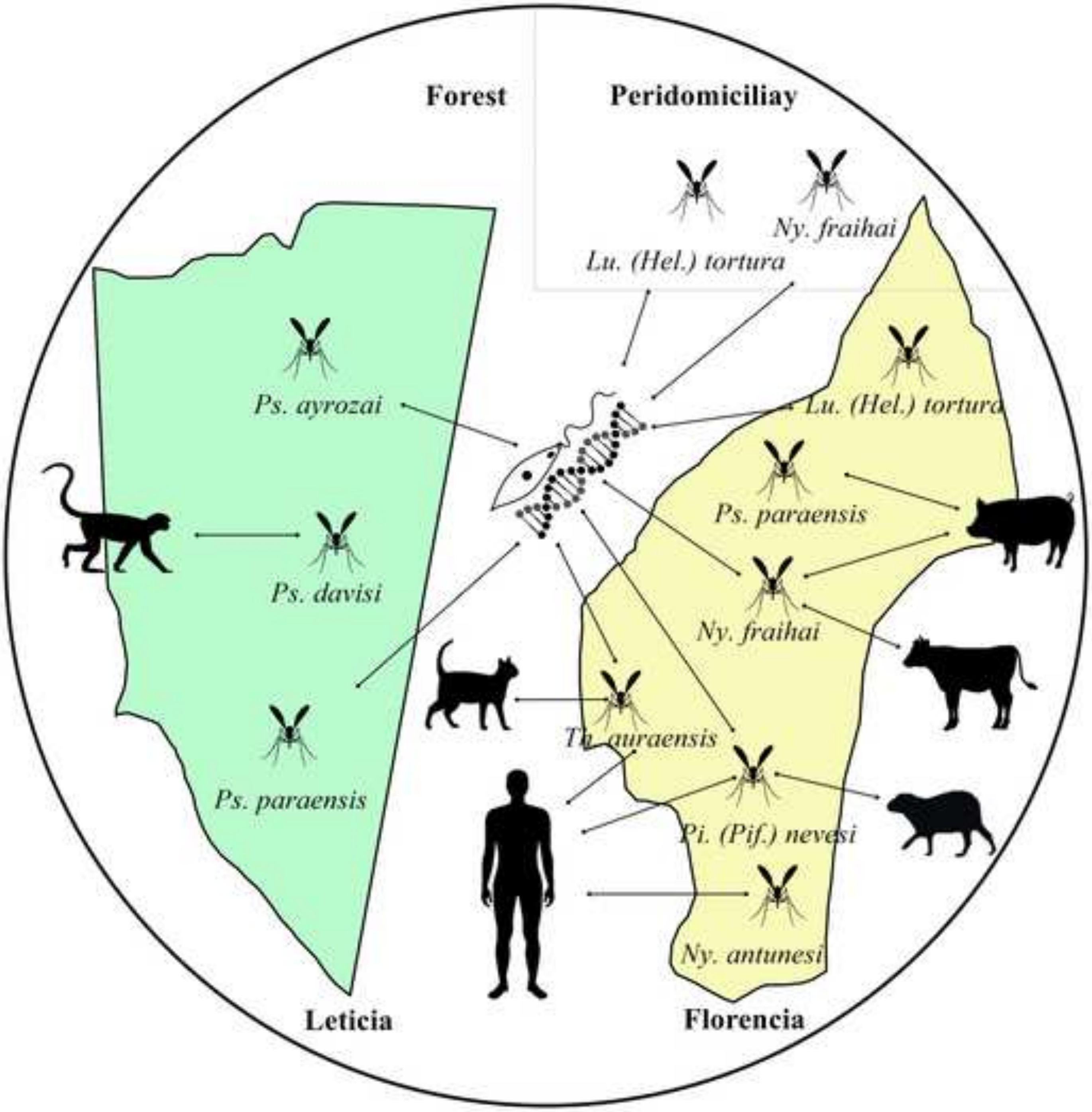

Table 1. Taxonomic inventory of phlebotomine sand flies, sex and feeding status of the females collected in the departments of Amazonas (Tanimboca and San Pedro de los Lagos localities) and Caquetá (Sebastopol, Jericó, Macagual, and Santo Domingo localities).

				**Caquetá**							
	**Caraño**				**Santo Domingo**			**San Martín**			
**ol**	**Jericó**			**Santo Domingo**			**Macagual**			**Tanimbo**	
**Male**	**Female**	**Female B-F**	**Male**	**Female**	**Female B-F**	**Male**	**Female**	**Female B-F**	**Male**	**Female**	**Fe**
-	-	-	1	-	-	-	-	-	-	-	
-	-	2	-	-	-	-	-	-	-	-	
-	-	-	-	-	-	-	-	-	-	-	
-	4	9	73	-	-	1	1	-	-	-	
-	-	-	-	4	1	13	101	1	-	-	
-	28	4	-	40	1	7	17	-	-	-	
-	-	-	-	-	-	-	-	-	-	1	
-	-	2	1	17	8	8	166	1	1	-	
-	1	-	-	-	-	1	-	-	-	-	
-	-	-	-	25	-	15	-	-	-	-	
-	-	-	-	-	-	1	-	-	-	-	
2	-	-	-	-	-	-	-	-	-	1	
-	-	-	-	2	1	2	-	-	-	-	
-	-	-	-	3	-	2	-	-	-	-	
13	-	-	-	2	-	5	-	-	-	-	
-	-	-	-	5	-	-	-	-	-	-	
-	-	-	1	6	-	3	-	-	-	-	
-	1	-	-	-	-	-	-	-	-	-	
6	-	-	-	-	-	-	-	-	-	-	
-	-	-	-	-	-	1	-	-	-	-	
-	-	-	-	1	-	1	-	-	-	-	

-	-	-	-	1	-	-	-	-	-	-	
-	-	-	-	-	-	-	-	-	-	1	
-	-	-	-	-	-	-	-	-	-	-	
-	112	8	116	-	-	-	-	-	-	-	
-	-	-	2	-	-	-	-	-	-	-	
-	-	-	-	-	-	-	-	-	-	-	
-	-	-	-	-	-	-	-	-	-	-	
-	-	-	-	-	-	-	-	-	-	1	
-	-	-	-	-	-	-	-	-	-	-	
-	11	1	3	8	-	3	8	1	-	1	
(43,75)	157 (41,32)	26 (6,84)	197 (51,84)	114 (60,64)	11 (5,85)	63 (33,51)	293 (98,65)	3 (1,01)	1 (0,34)	5 (71,43)	2
)		380 (41,62)			188 (20,59)			297 (32,53)			7 (3,66
				913 (82,70)							

*****New record for the department of Amazonas, ****** New record for the department of Caquetá, ° New record for Colombia, + Species of epidemiological relevance, **N (%)**: Relative abundance of captured phlebotomine sand flies, **B-F**: Fed females.

Click here to access/download
Supporting Information - Compressed/ZIP File Archive
suplementary.zip
