# Supplementary material for: Taxonomic, molecular and ecological approach reveals high diversity of vector sand flies, varied blood source supply and a high detection rate of Leishmania DNA in Colombian Amazon region
Source: PLoS Negl Trop Dis. 2025 Sep 5;19(9):e0013445. doi: 10.1371/journal.pntd.0013445 (PMC12412933; doi:10.1371/journal.pntd.0013445)
Supplement: Attachment — Submitted filename: Response to Reviewers-080725.docx [file pntd.0013445.s013.docx]

**RESPONSE TO REVIEWERS**

Respected reviewer, thank you very much for taking the time to evaluate manuscript “Taxonomic, molecular and ecological approach reveals high diversity of vector sand flies, varied blood source supply and a high detection rate of *Leishmania* DNA in Colombian Amazon region” and leave your valuable considerations, which were undoubtedly very useful to improve the manuscript.

Below you will find detailed revisions with their respective responses.

**Recent suggestions 07–07–2025**

**Funding**: Please amend your detailed Financial Disclosure statement

**Response**: The modification is made, resulting in the following text: “This study received financial support from project Hermes 57545 of the Universidad Nacional de Colombia, "Alianza estratégica interdisciplinaria Leticia, Medellín y La Paz para el estudio del microbioma de insectos vectores de enfermedades tropicales y su relación con el cambio climático y la sociedad", Convocatoria Nacional para el Fomento de Alianzas Estratégicas interdisciplinarias que articulen los procesos misionales de la Universidad Nacional de Colombia 2022–2024, awarded to CXMH and Scholarship Program of Ministerio de Ciencia, Tecnología e Innovación, Call 15, for Human Capital Development in the context of the Bicentennial and the 2021–2022 Biennial Plan, awarded to KCT. The funders had no role in study design, data collection and analysis, decision to publish, or preparation of the manuscript”.

**Data access**: “Please note that, though access restrictions are acceptable now, your entire minimal dataset will need to be made freely accessible if your manuscript is accepted for publication. This policy applies to all data except where public deposition would breach compliance with the protocol approved by your research ethics board. If you are unable to adhere to our open data policy, please kindly revise your statement to explain your reasoning and we will seek the editor's input on an exemption”.

**Response**: We understand the importance of data access regulation, accept and adhere to the open access policies of for publication.

Initially, we plan to release the sequences on December 19–2025, or once we have the concept of acceptance of the manuscript. However, if necessary, we will proceed to process the corresponding release as soon as possible.

**Conflict of interest statement**: “We have modified your conflict of interest statement to conform to the style of the magazine. Please double-check the statement and let us know if there are any errors.”

**Response**: We confirm that the amended statement is correct.

**Graphic Summary, and Figures:** Confirm if you have edited your own images in the programs listed or if you have used elements of the programs such as icons or images, provide a link to the license explaining copyright and reuse.

**Response**: No predesigned images or icons from the free software programs used were used. These programs were used exclusively to edit the scientific names of the images and to organize and design the composition from images, silhouettes and icons of their own creation.

**PRINCIPAL REVISIONS**

**Figure 8B:** The male genitalia of *Th*. *auraensis* appears similar to that of *Th*. *cellulana*. “It is possible that the neighbor-joining gene tree of the sand fly COI barcode sequences reflects problems with the identification of *Th*. *auraensis*. It is possible that *Th*. *auraensis* exists alongside *Th*. *cellulana*. In this case, I believe that morphologically the females are not distinguishable by their spermathecae. Therefore, the results obtained would be related to the genus and not to the species”.

**Response**: Dear Dr., there is indeed an inconsistency with the taxonomic assignment of *Th*. *auraensis*. After a detailed review of all collected specimens which are mounted on Euparal medium, we confirmed that they correspond to *Th*. *cellulana*.

The doubt in the original assignment originated mainly from the morphology of the parameres, however, after close observation, we identified that some variations were associated with the mounting of the specimens. This allowed us to recognize more clearly the specific diagnostic features of *Th*. *cellulana*.

Male genitalia of *Th*. *cellulana*. Red arrow pointing to the characteristic paramere of this species. Black arrow pointing to a paramere with a different visual position, which caused taxonomic doubts.

Consequently, we have corrected the taxonomic assignment throughout the document, including figures and supplementary information, replacing *Th*. *auraensis* with *Th*. *cellulana*.

Accordingly, we have corrected the taxonomic assignment throughout the document, including figures and supplementary information, replacing *Th*. *auraensis* with *Th*. *cellulana*. We have also notified GenBank to make the corresponding change in the species associated with the sequences presented above.

It should be noted that this correction does not generate substantial modifications in the content of the manuscript, as it is related to the taxonomic inventory and the presentation of the results. However, the pertinent adjustments have also been made in the discussion section, as shown below.

**Line 669 to 680:** Previous version text:

“However, studies in the Brazilian Amazon evidenced that *Th. auraensis* and *Ny. antunesi* represented approximately 40% of the fauna captured [65,66]. Notably*, Th. auraensis* has been found to be naturally infected with *Le.* (*Vi.*) *lainsoni*, *Le*. (*Vi*.) *braziliensis* and *Le*. (*Vi*.) *guyanensis* DNA in Peru and Brazil [67,68], where its possible participation in the transmission of this parasite has been reported”.

**Corrected version text:** “Since the description of *Th*. *cellulana* in Caquetá in 1979 [62], it had not been recorded in this department until the present study. To date, its distribution is exclusively Amazonian, including regions of the Colombian Amazon (departments of Guaviare and Putumayo) [63,64], Peruvian and Ecuadorian [65,66], where the reported abundance does not exceed 3% of the total composition. Although the epidemiological importance of this species has not yet been explored, the growing interest in the ecoepidemiological aspects associated with the genus *Trichophoromyia* has led to consider several of its species as suspected vectors [67]. Such is the case of two of the five species recorded in Caquetá, *Th*. *ubiquitalis*, found with flagellates of *L*. (*V*.) *lainsoni* in Brazil [68], and detected with DNA from *L*. (*V*.) *lainsoni*, *L*. (*L*.) *amazonensis* and *L*. (*V*.) sp. in Brazil and Ecuador [69–71]. And *Th*. *auraensis* has been found infected with DNA from *L*. (*V*.) *braziliensis*, *L*. (*V*.) *lainsoni* and *L*. (*V*.) *guyanensis* in Peru and Brazil [72,73]”.

**Line 802 to 812**: Previous version with highlighted text:

“… In addition, *Leishmania* sp. DNA was detected, reinforcing the connection between sources of ingestion and possible infection. Previously, *Th. auraensis* had been identified with *Le.* (*Vi.*) *lainsoni* [67] and *Le.* (*Vi.*) *braziliensis* in Peru and Brazil [67,116]. In particular, the detection of DNA from *F. catus* is relevant, since this vertebrate has been sporadically reported as a host of *Le. infantum* in the Brazilian Amazon [117]”.

**Corrected version with highlighted text:** “…In addition, *Leishmania* sp. DNA was detected, reinforcing the connection between sources of ingestion and possible infection. Although not previously recorded in this species, this finding underscores the importance of focusing future research on ecoepidemiological aspects that will provide accurate information on the possible role of *Th*. *cellulana* in *Leishmania* transmission”.

**Figure 5C:** The genus *Psychodopygus*, seem have identification problem, represents the spermathecae of *Ps*. *amazonensis*, appears to be from *Ps*. *davisi*.

**Response**: Dear Dr., there is indeed an inconsistency between the morphology of the specimen shown in the photograph with respect to the scientific name recorded in it.

It is an error when editing the scientific name in the photograph, however, to verify that there is no error in the identification, all specimens of these two species were reviewed to corroborate the correct identification.

Indeed, within the inventory, the identifications are correct, however, to show evidence of this, we attach the photographs of the species.

**Figure 5G:** The photo of the spermathecae of *Psychodopygus* *paraensis* is very similar to those of *Ps*. *ayrozai*. The reviewer recommends checking the species identification not only by the spermathecae, but also by observing the characteristics of the cibarium of the females.

**Response**: There is indeed an inconsistency between the morphology of the specimen shown in the photograph with respect to the scientific name recorded in it.

It is an error when editing the scientific name in the photograph, however, to verify that there is no error in the identification, all specimens of these two species were reviewed to corroborate the correct identification.

Indeed, within the inventory, the identifications are correct, however, to show evidence of this, we attach the photographs of the species.

It should be noted that in the identification, not having the thorax in all species, we took into account the characters of the head as the cibarium, where in these species the presence of the central dental monticule helps us to confirm the presence of *Ps*. *paraensis*.

**Note**: In view of the above modification, the entire document has been updated to reflect this change.

**REVIEWER 1**

**Line 28**: In the phrase “including vectors of *Leishmania* mainly in the genus *Lutzomyia and Psychodopygus*” change the word cursive format “*and*” for “and”.

**Response**: The changeover to without italics format is performed.

**Line 43**: Replace in the following phrase “A total of 1,104 specimens were collected, representing 12 genera and 30 species, eleven are recognized vectors of *Leishmania*” the word “eleven” for numeric data.

**Response**: The suggestion is confirmed, however, when the species *Th*. *auraensis* is replaced by *Th*. *cellulana*, the number of epidemiologically important species becomes 10. In this case, “11” is replaced by “10”.

**Line 48**: It is suggested to use a number after the comma in data associated with percentages.

**Response**: The change is made to the entire document, tables, images and supplementary information.

Example of corrected section: “*Homo sapiens* (28.8% Cytb; 18.6% 12S) and *Sus scrofa* (16.9% Cytb; 6.8% 12S) were the main food sources detected. While *Nyssomyia fraihai* (2.6%), *Trichophoromyia cellulana* (1.3%), *Nyssomyia yuilli pajoti* (1.3%) and *Evandromyia* (*Aldamyia*) *walkeri* (1.0%) grouped the highest detection rate of *Leishmania* DNA (9.0%)”.

**Line 68**: By referring the data only to cutaneous and mucosal leishmaniasis, in the text “Leishmaniases are a group of zoonotic diseases caused by *Leishmania*”, it is suggested to speak of Tegumentary Leishmaniases, since *L*. *infantum* didn't is included.

**Response**: The phrase Tegumentary is included in the text.

**Line 68 to 70**: In accordance with the taxonomic rules, it is suggested to eliminate the italic formatting of parenthesis.

Example of original section: *Leishmania (Le.) Leishmania* *(Le.) amazonensis, Le. Viannia (Vi.) braziliensis, Le. (Vi.) guyanensis, Le. (Le.)* 70 *mexicana,* and *Le. (Vi.) panamensis* in the Colombian Amazon region [1,2].

**Response**: The change is made to the entire document, tables, images and supplementary information.

**Line 72**: Within the text “Phlebotominae Rondani, 1840” it is suggested to remove the author from the family Phlebotominae.

**Response**: The author's reference to the family Phlebotominae is deleted.

**Line 72**: In the text “A significant number of leishmaniasis cases (4,403) were confirmed in the Colombian Amazon region alone” It is suggested to specify to which clinical form of Leishmaniasis the cases refer to.

**Response**: It is specified that more than 95% of the cases are cutaneous leishmaniasis.

**Line 78 to 79**: In the phrase “increase of pathogen transmission risk, not only of parasites, but also of viruses and bacteria associated with these vectors”, It is suggested to delete the text after “transmission risk”.

**Response**: Phrase “not only of parasites, but also of viruses and bacteria associated with these vectors” deleted.

**Line 86**: In the phrase “phlebotomine sand flies [11], increasing their interaction with wild and domestic” It is suggested that the word “phlebotomine” be deleted.

**Response**: The word “phlebotomine” is deleted and the document, tables, images and complementary information are unified.

**Line 88 to 89**: In the text “potential reservoirs of *Leishmania* [10,12,13] and/or other microorganisms of public health interest such as *Bartonella bacilliformis* [14]”, delete the words “of *Leishmania*”, “and/or other”, “of public health interest such as *Bartonella bacilliformis*”.

**Response**: The words are eliminated, and the following result is generated “but also as potential reservoirs of microorganisms [10,12–14].”.

**Line 93 to 94**: It is suggested to remove author and year for all species, since if this information is included for each sand flies species, it is necessary to include it for all species (*Leishmania*, hosts, etc.) in the document.

Example of original section: “*Psychodopygus (Ps.) ayrozai* (Barretto & Coutinho, 1940) which, besides resenting blood traces of *Homo (H.) sapiens,* was also associated with other sources of ingestion”.

**Response**: The author and year of related species are removed throughout the document, tables and supplementary information, in order to provide uniformity.

Example of corrected section: “*Psychodopygus ayrozai* which, besides resenting blood traces of *Homo sapiens*, was also associated with other sources of ingestion”.

**Line 98 to 99**: In the text “in phlebotomine sand flie of *Nyssomyia (Ny.) antunesi* (Coutinho, 1939) [16]. *Trypanosoma minasense* DNA” delete the phrase “phlebotomine sand flie of”.

**Response**: The suggested sentence is eliminated.

**Line 107 to 118**: It is suggested to rewrite and reduce the paragraph, so that it is shorter and less confusing.

Example of original section: “Due to the above, it is imperative to expand studies on blood ingestion in phlebotomine sand flies, which play a critical role as primary or secondary vectors, and that are involved in the increase of cutaneous leishmaniasis (CL) transmission risk, mainly in urban and rural areas of the Colombian Amazon [24,25]. Understanding the ecological interactions occurring in the Amazon region is essential to elucidate the *Leishmania* transmission cycle and assess whether habitat disturbances have facilitated the geographic expansion of vectors, parasites, and vertebrate hosts, especially in border areas [26], considering that previous studies have been conducted in the Andean and Caribbean regions of Colombia and in the Brazilian Amazon, demonstrating how the habitat disturbance of these insects favors the feeding plasticity of species such as *Ps. panamensis* (Shannon, 1926) and *Ny. antunesi,* while the Colombian Amazon region remains understudied in this aspect [16,18,19,27,28]”.

**Response**: The text is rewritten and reads as follows “Therefore, it is essential to understand the extent of dietary plasticity of these insects, especially in areas of the Colombian Amazon, where the availability of vertebrates could influence their role as primary or secondary vectors mainly of cutaneous leishmaniasis (CL) [24,25]. Which contributes to a better understanding of the epidemiology of the disease, particularly in border areas [26]”.

**Line 141 to 144**: In the text “this methodology has not been adopted by local health entities, as resource allocation tends to prioritize the surveillance of other diseases such as malaria and dengue due to their high incidence and public health impact” it is considered that “cost of this type of technique for health services is not yet a reality”.

**Response**: To avoid attributing the cost of the methodology as a possible cause of its non-implementation in the health entities, it has been decided to delete this sentence from the document.

**Line 154 to 155**: It is recommended to delete the following text “In this regard, studies such as the one conducted by Posada-López *et al*. are significant, as using integrative taxonomy from specimens collected in Leticia, Amazonas, led to the identification of 61 new mitochondrial haplotypes associated with 24 species and, for the first time, assigned Barcode sequences of *Evandromyia (Ev.) (Evandromyia)(Eva.) georgii* (Freitas & Barrett, 2002), *Psathyromyia (Pa.) (Psathyromyia)(Psa.) punctigeniculata* (Floch & Abonnenc, 1944) and *Ny. yuilli pajoti* (Abonnenc, Léger& Fauran 1979) [29]. Although it is currently the only study integrating classical and molecular taxonomy in the region, the results highlight the need for further research to expand the availability of sequences, particularly of missing species or those of epidemiological importance in the Amazon. This is especially critical in the department of Caquetá, where studies are even more limited”, due to the length of the introduction and it is not necessary.

**Response**: Suggested text is deleted.

**Line 160**: It is recommended to standardize the use of the phrase “sandfly” or “sand fly”.

**Response**: Is standardized with the phrase “sand fly”, is reviewed in the document, tables and supplementary information.

**Line 185**: In the text “The study area is part of the Colombian Amazon region, characterized by the presence

185 of tropical rainforest (Bh-T) (Holdridge, 1996)”, the text reference is requested to be replaced by a numerical reference.

**Response**: The textual reference is replaced with numerical reference.

**Line 192**: At the beginning of text “sand fly collection was conducted in secondary and primary forests”, modification of the letter is requested “s” by capital letter “S”.

**Response**: The change to “Sand fly” is made.

**Line 194 and 197**: In the phrase “*Canis (C.) lupus* (dogs), *Felis (F.) catus* (cats) and *Gallus (G.)*” remove italic formatting applied to parentheses.

**Response**: We have applied the suggested format of italics in parentheses throughout the document. In addition, and following the recommendation of reviewer 2, in lines 93 to 94, we have eliminated the nomenclature of the genera when it appeared in parentheses.

Example of corrected section: “*Canis lupus* (dogs), *Felis catus* (cats) and *Gallus gallus*”

**Line 212 to 219**: In the text “Two collection campaigns were performed in Caquetá and Amazonas departments in August and November 2023 respectively, for three consecutive days per sampling area. Twelve CDC (Centers for Disease Control and Prevention) light traps were installed for 12 hours (18:00 to 6:00) in the intra, peri and extra-domicile. Each collection point was georeferenced with a GPSmap_60CSx, GARMIN. A Shannon trap was installed between 18:00 and 19:00 for one night at the point of major collection, while active search was conducted day and night in resting phlebotomine sites with entomological aspirators and Prockopack® in potential spots such as broiler houses or poultry farms”, it is suggested to specify how many collection campaigns were carried out per department, and it is requested to include the sampling effort for each department or locality studied.

**Response**: The collection information is edited, and the following modification is generated “During the year 2023, two collection campaigns were carried out, the first in August in Caquetá and the second in November in Amazonas. In each locality of both departments, 12 CDC (Centers for Disease Control and Prevention) light traps were installed in intra, peri and extradomiciliary areas, where they operated for 12 hours (18:00 to 6:00) each night, for three consecutive days. On the second night of collection, a Shannon trap was set up for one hour (18:00 to 19:00) at the point where the CDC traps recorded the highest abundance of sand flies on the first night. The collection was complemented with a search in potential resting sites, such as walls chicken coops, rabbit hutches and pig housing, using mouth and Prockopack [46] aspirators up for one hour (18:00 to 19:00). Each collection point was georeferenced with a GPSmap_60CSx, GARMIN”.

**Line 219**: The evaluator asks ¿Prokopack Aspirator?

**Response**: The Prokopack is a device often used for the collection of Culicidae, mainly *Aedes* *aegypti*. However, in this study, its implementation was very useful in the collection of sand flies that were resting on the internal walls of poultry and swine farms. The device made it possible to collect a large number of specimens efficiently and without fragmenting them. This methodology prevents professionals from inhaling gases, microorganisms or excrement particles present in animal housing.

**Line 226**: In the text “for taxonomic allocation using the Galati 2024 nomenclature system”, it is suggested to remove the author's year and replace the phrase “nomenclature system” with “classification”.

**Response**: The suggested correction is made.

**Line 232**: In the phrase “The use of generic abbreviations” include “and subgeneric”.

**Response**: The change is made, leaving the text as follows “The use of generic and subgeneric abbreviations”.

**Line 233**: In the text “was conducted following the scheme proposed by Marcondes, 2007 [45]”, delete “the scheme proposed by” and “, 2007”.

**Response**: The suggested correction is made.

**Line 242 and 243**: The evaluator asks: ¿Females with blood and without blood were separated for analysis?.

**Response**: Yes ma'am, the females fed were differentiated and separated for analysis, however, for clarity the following information is included in the document “Individual abdomens from fed females were differentiated, separated and preserved in RNAlater until DNA extraction and blood source identification. Previous to extraction and in order to separate the tissue from the preservative salts, each specimen was transferred under sterile conditions to a new vial and washed with 70% ethanol for one minute at 2.000 revolutions per minute (rpm), then transferred to a new vial and washed with ultrapure water for one minute at 2.000 rpm, for finally transferred to the vial where DNA extraction would be performed” within the methodology “Molecular analyses: DNA extraction of sand flies”.

**Line 254**: In the text “*Ny. antunesi* DNA was included as a positive control”, explain the specie's choice.

**Response**: *Ny*. *antunesi* is an important vector that is part of the fauna of the study region. Previously we had DNA extracted, which resulted negative for *Leishmania* and negative for blood source detection, for this reason and for its quality in previous PCR processes, we considered it a good candidate as a control in this study. However, to clarify the reason for its use, we include the following in this document: “*Ny. antunesi* DNA was previously used in PCR standardization, whose identity was confirmed by sequencing, and which was negative for *Leishmania* infection and blood ingestion, was included as a positive control”.

**Line 288**: The reviewer asks if the *Ny*. *antunesi* DNA mentioned in the manuscript is it of laboratory or colonization.

**Response**: *Ny*. *antunesi* is an important vector that is part of the fauna of the study region. Previously we had DNA extracted, which resulted negative for *Leishmania* and negative for blood source detection, for this reason and for its quality in previous PCR processes, we considered it a good candidate as a control in this study. However, for clarity on the provenance of the species, we include in the document the following phrase “*Ny*. *antunesi* of wild type”.

**Line 333**: In the phrase “Standardized Index of Species Abundance (SISA), described by Roberts and Hsi (1979)”, the text reference is requested to be replaced by a numerical reference.

**Response**: The textual reference is replaced with numerical reference.

**Line 334**: In the text “the ANAFAU software, following the methodology described by Silveria *et al.* 1995” it is requested to remove the year of reference.

**Response**: The year “1995” is removed from the text.

**Line 351**: In the following text “A total of 1,104 phlebotomine sand flies were collected during the entomological survey, 352 with females…”, delete the underlined phrase.

**Response**: Deleted text.

**Line 356 to 269**: Delete the text “The genera *Micropygomyia* Barretto, 1962 and *Brumptomyia* França & Parrot, 1921 were each represented by a single species (Table 1).

*Viannamyia (Vi.) caprina* (Osorno-Mesa, Morales & Osorno, 1972) (1 ♀), *Vi. tuberculata* (Mangabeira, 1941) (1 ♀) and *Sciopemyia (Sc.) fluviatilis* (Floch & Abonnenc, 1944) (3 ♀) were recorded for the first time in Amazonas, the latter representing the first record for Colombia, as far as we know.

Additionally, in Caquetá, several species were reported for the first time: *Brumptomyia (Br.) mesai* Sherlock, 1962 (1 ♂), *Ev.* *(Ald.) georgii* (2 ♀), *Ny. fraihai* (Martins, Falcão & Silva, 1979) (98♀, 7♂), *Ny. yuilli pajoti* (195♀, 10♂), *Lu. Tricholateralis (Trl.) sherlocki* Martins, Silva & Falcão, 1971 (1♀, 1♂), *Lu. Helcocyrtomyia (Hel.) tortura* Young & Rogers, 1984 (25♀, 15♂), *Ps. carrerai thula* (Young, 1979) (3♀, 2♂), *Trichophoromyia (Th.) howardi* (Young, 1979) (2 ♂) and *Pintomyia (Pi.) Pifanomyia (Pif.) serrana* (Damasceno & Arouck, 1949) (1♀, 1♂) (Table 1).” Since the information is contained in Table 1.

**Response**: The suggested text is eliminated; however, the detailed information on the species that make up the new reports is used to expand the discussion on line 643 to 647 at the suggestion of evaluator 2.

**Line 389**: Requests to include within the legend of Fig 2 the meaning of the dashed line that is evident in the image.

**Response**: The meaning of the dashed line is clarified.

**Line 401**: In the analysis of the distribution and diversity of species, according to Silveira et al. the correct term for species with few specimens collected is accidental instead of “Rare”, the change of term is requested.

**Response**: The word “Rare” is replaced by “Accidental” and modified throughout the document.

**Line 405**: In the text “*Pa. Foratiiniella (For.) aragaoi*” delete the word “*Foratiiniella*” and leave only the initial of the gender in parentheses.

**Response**: The nomenclature of all species in the document is corrected and unified.

**Line 626**: Reviewer's question: ¿Why was the *Leishmania* species not identified?.

**Line 669**: The evaluator has doubts about the absence of *Leishmania* genotyping.

**Response for two questions**: Our initial objective was to detect the presence of *Leishmania* DNA in the collected specimens, with the purpose of estimating detection rates. Although taxonomic differentiation is essential, it was not possible to consistently sequence the amplicons obtained, possibly due to DNA degradation or low concentration. We recognize that ITS1-2 are good markers and widely used for the detection and genotyping of *Leishmania* species. However, at the time of this study we did not have a standardized protocol or the availability of primers for their application. In future studies, complementary analyses using more specific markers and more sensitive techniques, such as the standardization and use of ITS1-2 markers, nested PCR, qPCR and next-generation sequencing (NGS), are contemplated to achieve a more accurate characterization of the species circulating.

**Line 632 to 636**: It is suggested to delete figure 14, since it does not represent decisive information to be included in the main document.

**Response**: Figure 14 is removed from the document, however, it is sent to supplements with the code “Fig S2” in order to have the information available for any questions.

**Line 661**: In the discussion text "Colombian Amazon, a region of high biodiversity and great vulnerability to the effects of climate change and human intervention.", ¿has it been evaluated? Please indicate the results.

**Response**: Although the effects of climate change and human intervention in the study areas were not directly evaluated in this research, it is considered pertinent to mention them as relevant contextual factors. To support this statement and give greater clarity to the text, a reference to this information was included. Consequently, the text was amended as follows: “Colombian Amazon, a region of high biodiversity and great vulnerability to the effects of climate change and human disturbances [58]”.

**Line 678-679**: In the underlined text “This can be attributed to the ecotope selected for collection, the number of traps used, the sampling effort time, and differences in bioclimatic variables recorded during collection”, the evaluation variables were not presented in the document.

**Response**: Related text is eliminated, and the focus is on the variables presented in the research, as follows: “This can be attributed to the ecotope selected for collection, the number of traps used, sampling effort”.

**Line 695**: In the text “Notably*, Th. auraensis* has been found to be naturally infected with *Le.* *(V.) lainsoni* [63] and *Le. (V.) braziliensis* [63,64] in Peru and Brazil, where is considered a putative vector of *Le. Viannia* [65]” the concern is generated by the phrase “putative vector of *Le. Viannia*”.

**Response**: The paragraph is replaced by information on *Th*. *cellulana* and the epidemiological relevance of *Th*. *ubiquitalis* and *Th*. *auraensis*, which also circulate in Caquetá, is highlighted.

**Line 709 to 717**: About the paragraph “The presence, absence, abundance, and richness of the species recorded in this study, compared with those previously reported, may differ due to changes in environmental conditions and the level habitat disturbance at the time of collection, as fluctuations in temperature, precipitation and humidity, combined with an increasing anthropogenic pressures, has favored heat waves and prolonged periods of drought, that may reduce the survival of some species, but can also accelerate immature development and promote the adaptation of others to new environments [10]. This dynamic alters vector composition and ecology, favoring their expansion in rainy seasons and their presence in previously non-endemic areas [10,71]” it was not observed on the methodology.

**Response**: Given that the following research did not include an exhaustive sampling of data associated with climate change and environmental conditions that could be comparable with previous studies in which this information is also lacking, it is considered that the paragraph should be deleted.

**Line 729 and 730**: It is suggested to include the information about the food source of the *Sciopemyia* genus: Costa JCR, Marchi GH, Santos CS, Andrade MCM, Chaves Junior SP, Silva MAN, Melo MN, Andrade AJ. First molecular evidence of frogs as a food source for sand flies (Diptera: Phlebotominae) in Brazilian caves. Parasitol Res. 2021 May;120(5):1571-1582.

**Response**: We include the citation and generate an update on the species of the genus and medical importance.

**REVIEWER 2**

**Line 28**: In the phrase “including vectors of *Leishmania* mainly in the genus *Lutzomyia and Psychodopygus*” change the word cursive format “*and*” for “and”.

**Response**: The changeover to without italics format is performed.

**Line 68 to 70**: In the text “Leishmaniases are a group of zoonotic diseases caused by *Leishmania (Le.) Leishmania (Le.) amazonensis, Le. Viannia (Vi.) braziliensis, Le. (Vi.) guyanensis, Le. (Le.) mexicana,* and *Le. (Vi.) panamensis* in the Colombian Amazon región” it is suggested to first refer to the subgenres “*Leishmania (Le.)* and *Viannia* (*Vi.*)” and then list the species in order “*Le*. (*Le*.) *amazonensis, Le*. (*Le*.) *mexicana*, *Le*. (*Vi*.) *brazilensis*, *Le*. (*Vi*.) *guyanensis* and *Le*. (*Vi*.) *panamensis*”.

**Response**: The suggestions made are included and the text is as follows: “Tegumentary Leishmaniases are a group of zoonotic diseases caused by *Leishmania* of the subgenera *Leishmania* and *Viannia*, *L.* (*L*.) *amazonensis*, *L*. (*L*.) *mexicana*, *L*. (*V*.) *braziliensis*, *L*. (*V*.) *guyanensis*, and *L*. (*V*.) *panamensis* in the Colombian Amazon region.

**Line 72**: The correct inclusion of the authors is requested of the subfamily Phlebotominae, switch from “subfamily Phlebotominae Rondani, 1840” to “subfamily Phlebotominae Rondani & Berté, 1840”.

**Response**: The suggestion is considered; however, according to reviewer 1 “It is suggested to remove author and year for all species, since if this information is included for each sand flies species, it is necessary to include it for all species (*Leishmania*, hosts, etc.) in the document", therefore the author and year of related species are removed throughout the document, tables and supplementary information, in order to provide uniformity.

**Line 93 to 94**: “According to the international code of zoological nomenclature, this way of citing means that this species belongs to the subgenus *Psychodopygus (Ps.*)” it is suggested to remove the nomenclature of the genus in parenthesis.

Example of original section: “sand flies in disturbed habitats increased the risk of contact with vertebrate reservoirs like *Psychodopygus (Ps.) ayrozai* which, besides resenting blood traces *Homo (H.) sapiens*”.

**Response**: Nomenclature associated with gender is eliminated, the change is made throughout the document, tables and supplementary information.

Example of corrected section: “sand flies in disturbed habitats increased the risk of contact with vertebrate reservoirs like *Psychodopygus ayrozai* which, besides resenting blood traces of *Homo sapiens*”.

**Line 130 to 131**: The deletion of the parentheses of the authors of the phlebotomine genera is requested.

**Response**: The suggestion is considered; however, according to reviewer 1 “It is suggested to remove author and year for all species, since if this information is included for each sand flies species, it is necessary to include it for all species (*Leishmania*, hosts, etc.) in the document", therefore the author and year of related species are removed throughout the document, tables and supplementary information, in order to provide uniformity.

**Line 148 to 149**: It is suggested to eliminate the nomenclatures of genera and subgenera that are represented in parentheses.

Example of original section: “first time, assigned Barcode sequences of *Evandromyia (Ev.) (Evandromyia (Eva.) georgii*”*.*

**Response**: The nomenclature of gender and subgenre is eliminated from the entire document, tables and supplementary information.

Example of corrected section: *Evandromyia* (*Ev.*) *georgii* if the first cite is made and *Ev.* (*Eva*.) *georgii* if cited a second time.

**Line 178 and 179**: It is suggested to change the language of the longitude symbology associated with the Amazon coordinates from Spanish “O” to English “W”.

**Response**: The language of the symbology is changed.

**Line 198**: In the phrase “*F catus*” it is suggested to include punctuation after the initial of the gender.

**Response**: Punctuation is included after the gender.

**Line 217**: It is suggested to specify the time measurement “hours” in the following text “18:00 and 19:00 for one night at the point of major collection”.

**Response**: Phlebotomine collection times are specified.

**Line 226**: In the text “(Carl Roth GmbH + Co. KG, Alemania)” translate the country related to the reference reagent into English.

**Response**: Translation done.

**Line 359 to 369**: It is suggested to eliminate the nomenclatures of genera that are represented in parentheses and replace the initial nomenclature of the subgenus with the full name.

Example of original section: “*Sciopemyia (Sc.) fluviatilis”* and “*Lu. Helcocyrtomyia (Hel.) tortura*”.

**Response**: The nomenclature of genre and subgenre is eliminated and unified in the document as follows: *Sciopemyia fluviatilis* if the first cite is made and *Sc. fluviatilis* if cited a second time. Or *Lutzomyia* (*Hel*.) *tortura* if the first cite is made and *Lu*. (*Hel*.) *tortura* if cited a second time.

**Line 418**: It is requested to include the word “and” in the text “(Shannon_H: 2.014 and 2.268)”.

**Response**: inclusion of the word “and” performed.

**Line 638:** Replace comma punctuation “,” to period “.” associated with data in percentages (n=8, 2,58%).

**Response**: Replacement of punctuation in all documentation, tables and supplementary information is performed.

**Line 668**: Correct the writing of the species “*Ny. pajoti*” by “*Ny. yuilli pajoti*”.

**Response**: The correction is made, and the spelling of the species is checked for homogeneity throughout the document.

**Line 694 to 695 and 700 to 701**: Review the nomenclature of *Leishmania* species, taking into account the previous nomenclature.

**Response**: The document is corrected and the homogeneity of the spelling of the species throughout the document is checked, resulting in the following form: *L*. (*V*.) *lainsoni*, *L.* (*V*.) *braziliensis*, *L.* (*L*.) *infantum*.

**Line 824**: Check the reference associated with the text “In particular, the detection of DNA from *F. catus* is relevant, since this vertebrate has been sporadically reported as a host of *Le. infantum* in the Brazilian Amazon [97]”, because “its authors report infection of F. catus by *Leishmania* *infantum* in Bahia, northeastern Brazil”.

**Response**: The reference is reviewed, and although it contains general information associated with the text, the article where the research and the results obtained are fully evidenced is directly searched, the text and the reference are updated as follows: “In particular, the presence of DNA from *F. catus* is relevant, as it has been detected naturally infected and categorized as harboring active of *L*. (*L*.) *infantum* in an endemic area of Brazil [104]”.

**Abtrac graphic**: Within the key findings, in the text “Gigh rate infection of *Leishmania*” Change the word “Gigh” to “High”.

**Response**: Change made.

**REVIEWER 3**

**Line 68 to 70, 88 and 89**: In the text “Leishmaniases are a group of zoonotic diseases caused by *Leishmania (Le.) Leishmania* *(Le.) amazonensis, Le. Viannia (Vi.) braziliensis, Le. (Vi.) guyanensis, Le. (Le.)* *mexicana,* and *Le. (Vi.) panamensis*” “*Leishmania*” and “*Bartonella bacilliformis*”, it is suggested to include the author and year of each taxon.

**Response**: The relevance of the suggestion is considered, however, according to reviewer 1 “It is suggested to remove author and year for all species, since if this information is included for each sand flies species, it is necessary to include it for all species (*Leishmania*, hosts, etc.) in the document", therefore the author and year of related species are removed throughout the document, tables and supplementary information, in order to provide uniformity.

**Line 97 and 98**: Correction of the spelling of the species of *Leishmania*. Example: “*Le.* (*Vi.*) *lainsoni, Le. (Vi). guyanensis*”.

**Response**: The entire document, tables and supplementary information is proofread and revised.

**Line 305**: In the sentence “COI, 12S rRNA, Cytb and HSP-70 N sequences were edited and aligned using the Finch” delete the HSP-70 N marker if it was not possible to obtain *Leishmania* sequences.

**Response**: The HSP-70 N marker associated with *Leishmania* was eliminated.

**Line 344**: in paragraph “To estimate the infection rate in positive *Leishmania* samples, the minimum infection rate is the following formula: Minimal Infection Rate (MIR) = Number of infected phlebotomine sand flies/Total phlebotomine sand flies tested x 100 [56]”. The reviewer asks “Here were the pools and the individuals? Was a pool considered a sample?.

**Response**: *Leishmania* detection was carried out on a total of 310 females. Of these, 182 were grouped into 51 pools composed of two to six specimens, while the remaining 128 were analyzed individually. *Leishmania* DNA was only detected in individual samples (N = 28), with no positive results in the pools.

Due to the absence of positivity in the pools, the Minimal Infection Rate (MIR) was calculated considering the total number of females analyzed (n = 310), without discriminating between individual samples and pools.

**Line 363**: It is suggested that the text “Additionally, in Caquetá, several species were reported for the first time: *Brumptomyia”* is better for discussion.

**Response:** Your valuable suggestion is considered, and the information is transferred for discussion on lines 644 to 648 as a complement to the new circulation records.

Example of corrected section: “In addition, three new circulation records are presented for Amazonas, *Vi*. *caprina*, *Vi*. *tuberculata* and *Sc*. *fluviatilis*, nine Caquetá, *Br*. *mesai*, *Ev*. (*Ald*.) *georgii*, *Ny*. *fraihai*, *Ny*. *yuilli pajoti*, *Lu*. (*Trl*.) *sherlocki*, *Lu*. (*Hel*.) *tortura*, *Ps*. *carrerai* *thula*, *Th*. *howardi* and *Pi*. (*Pif*.) *serrana*, and one for Colombia, *Sc*. *fluviatilis*, as far as we know”.

**Line 581 to 582**: Reviewer's question ¿why two molecular markers “Cytb and 12S” were used in the detection of sources of blood intake?.

**Response**: For the identification of blood ingestion sources, we used two molecular markers (Cytb and 12S rRNA) to increase the probability of host DNA detection at different levels of preservation.

The Cytb marker, widely used in dietary studies, is ideal for fresh samples, as it amplifies a highly variable region that allows accurate interspecific discrimination, supported by the abundant information available in databases. However, being a relatively long fragment, it is more susceptible to degradation during the digestion process in the insect gut, which can result in the loss of valuable information if the DNA is not amplified.

To mitigate this limitation, we complemented the analysis with the 12S rRNA marker that amplifies shorter fragments. Although it has a lower capacity for interspecific discrimination due to the low level of genetic variability, it is highly sensitive to degraded DNA or DNA present in low concentrations (frequent conditions in hematophagous insects) and allows to compensate for nonspecificities that may occur during Cytb amplification, such as the detection of insect DNA. Therefore, supplementing the detection with an additional marker contributes to a more accurate identification of blood sources.

However, in order to clarify this reason, lines 267 to 269 of the methodology include the following information: “To increase the probability of detecting host DNA at different levels of preservation within the gut of females identified with blood traces, two molecular markers were used in independent PCR reactions. In the first reaction…”

**Line 626**: The reviewer asks: ¿Why hasn't the specific species of *Leishmania* been identified?, ¿ The molecular marker Hsp-70 hasn't been sequenced? Why weren't the ITS1 or ITS2 markers used, which are widely used to identify the species of *Leishmania*?.

**Response**: Our initial objective was to detect the presence of *Leishmania* DNA in the collected specimens, with the purpose of estimating detection rates. Although taxonomic differentiation is essential, it was not possible to consistently sequence the amplicons obtained, possibly due to DNA degradation or low concentration. We recognize that ITS1-2 are good markers and widely used for the detection and genotyping of *Leishmania* species. However, at the time of this study we did not have a standardized protocol or the availability of primers for their application. In future studies, complementary analyses using more specific markers and more sensitive techniques, such as the standardization and use of ITS1-2 markers, nested PCR, qPCR and next-generation sequencing (NGS), are contemplated to achieve a more accurate characterization of the species circulating.

However, in order to clarify this reason, lines 836 to 839 of the discussion, include the following information: “Although in this study it was not possible to perform consistent sequencing of the *Leishmania* amplicons, possibly due to DNA degradation or low concentration. Future studies contemplate the use of more sensitive techniques, such as ITS1-2 markers, nested PCR, qPCR and NGS, for accurate characterization of circulating species”.

**REVIEWER 4**

**Line 121 to 125**: In the paragraph, the reviewer suggests a better description of the sand flies species diversity of the two study areas separately.

**Response**: Information on species separated by department is included and the text is worded as follows: “Specifically, in Belén de los Andaquíes, Milán, Solano and Solita Caquetá, the circulation of 46 species of sand flies distributed in 13 genera has been recorded, with *Psychodopygus* and *Psathyromyia* being the most abundant [20,24,29]. Meanwhile, in Amazonas, studies carried out mainly in the kilometers surrounding Leticia, Amacayacu National Natural Park and Puerto Nariño, have recorded 58 species belonging to 14 genera, the most representative being *Lutzomyia*, *Psathyromyia* and *Psychodopygus* [20,30–32]. These investigations have contributed to the knowledge on the distribution and diversity of sand flies, however, the role of the species in the transmission dynamics of the disease has yet to be addressed”.

**Line 217 to 218**: Provide more details about where the active searches with captors were carried out.

**Response**: Detailed information on collection sites with captors is included in the document: “The collection was complemented with a search in potential resting sites, such as walls chicken coops, rabbit hutches and pig housing, using mouth and Prockopack [46] aspirators up for one hour (18:00 to 19:00)”.

**Line 305**: In the sentence “COI, 12S rRNA, Cytb and HSP-70 N sequences were edited and aligned using the Finch” delete the HSP-70 N marker if it was not possible to obtain *Leishmania* sequences.

**Response**: Dear doctor, indeed, only DNA detection was performed, as it was not possible to consistently sequence the amplicons obtained, possibly due to DNA degradation or low concentration. For this reason, the information is eliminated from the methodology "Sequence analysis PCR products".

**Line 626**: If possible, insert the species of *Leishmania* found in the phlebotomines analyzed, as there is only a relationship between the blood food source data.

**Response**: Our initial objective was to detect the presence of *Leishmania* DNA in the collected specimens, with the purpose of estimating detection rates. Although taxonomic differentiation is essential, it was not possible to consistently sequence the amplicons obtained, possibly due to DNA degradation or low concentration. We recognize that ITS1-2 are good markers and widely used for the detection and genotyping of *Leishmania* species. However, at the time of this study we did not have a standardized protocol or the availability of primers for their application. In future studies, complementary analyses using more specific markers and more sensitive techniques, such as the standardization and use of ITS1-2 markers, nested PCR, qPCR and next-generation sequencing (NGS), are contemplated to achieve a more accurate characterization of the species circulating.

**Line 658**: To facilitate fluidity in reading the discussion, it is suggested that the discussion be detailed as the results section.

**Response**: The discussion was structured into subtopics to facilitate reading, improve the fluency of the text and avoid possible confusion.

**EDITOR'S SUGGESTIONS**

**Author Contributions:** Update the contributions of the co-authors taking into account the CRediT Taxonomy to describe the contributions individually.

**Response:** Co-author contributions are updated individually as detailed below, and are removed from the main document, as it is in a separate format at the time of submission.

**Katerine Caviedes-Triana**

**Roles:** Conceptualization, Methodology, Specimen Identification, Taxonomical Curatorship, Software, Validation, Formal Analysis, Investigation, Data Curation, Writing – Original Draft Preparation, Writing – Review & Editing, Visualization.

**E-mail:** kcaviedes@unal.edu.co

**Affiliation:** Grupo de Microbiodiversidad y Bioprospección, Faculty of Sciences, Universidad Nacional de Colombia, Medellín, Colombia. Street 59 A # 63-20, Medellín Postal Code 050003, PECET (Programa de Estudio y control de enfermedades tropicales), Facultad de Medicina, Universidad de Antioquia, Medellín, Colombia.

**Orcid:** [https://orcid.org/0009-0005- 5838-3782](https://orcid.org/0009-0005-5838-3782)

**Daniela Duque-Granda**

**Roles:** Conceptualization, Methodology, Writing – Review & Editing, Visualization, Investigation.

**E-mail:** daduquegr@unal.edu.co

**Affiliation:** Grupo de Microbiodiversidad y Bioprospección, Faculty of Sciences, Universidad Nacional de Colombia, Medellín, Colombia. Street 59 A # 63-20, Medellín Postal Code 050003.

**Orcid:** <https://orcid.org/0000-0002-4994-2686>

**Gloria Cadavid-Restrepo**

**Roles:** Conceptualization, Validation, Funding acquisition, Resources, Writing – Review & Editing, Supervision, Project Administration.

**E-mail:** gecadavi@unal.edu.co

**Affiliation**: Grupo de Microbiodiversidad y Bioprospección, Faculty of Sciences, Universidad Nacional de Colombia, Medellín, Colombia. Street 59 A # 63-20, Medellín Postal Code 050003.

**Orcid:** <https://orcid.org/0000-0003-1026-6358>

**Rafael Vivero-Gomez**

**Roles:** Conceptualization, Methodology, Taxonomical Curatorship, Software, Validation, Formal Analysis, Investigation, Resources, Data Curation, Writing – Original Draft Preparation, Writing – Review & Editing, Visualization, Supervision.

*** E-mail**: rjviverog@unal.edu.co

**Affiliation**: Grupo de Microbiodiversidad y Bioprospección, Faculty of Sciences, Universidad Nacional de Colombia, Medellín, Colombia. Street 59 A # 63-20, Medellín Postal Code 050003, PECET (Programa de Estudio y control de enfermedades tropicales), Facultad de Medicina, Universidad de Antioquia, Medellín, Colombia.

**Orcid:** <https://orcid.org/0000-0001-6768-4097>

**Claudia X. Moreno-Herrera**

**Roles:** Conceptualization, Funding acquisition, Methodology, Validation, Investigation, Project administration, Resources, Writing – Review & Editing, Visualization, Supervision.

*** E-mail:** cxmoreno@unal.edu.co

**Affiliation**: Grupo de Microbiodiversidad y Bioprospección, Faculty of Sciences, Universidad Nacional de Colombia, Medellín, Colombia. Street 59 A # 63-20, Medellín Postal Code 050003.

**Orcid:** <https://orcid.org/0000-0002-8132-5223>

**Author summary:** The inclusion of author summary is requested since it was not in the document.

**Response**: The author summary is included, after the abstract graphic.

**Line 39 and 219:** Removal of the logo associated with the registered trademarks is requested “®”.

**Response**: The trademark logo associated with the Prokopack device is removed and a bibliographic reference is included.

**Line 866:** It is recommended to include within the document the related citation of Figure 15 entitled **“**The ecological dynamics of the tripartite relationship among phlebotomine sand flies, blood meal sources and *Leishmania* in forest and peridomicialiary areas in the Amazon region from Colombia”.

**Response**: The respective citation of the figure is made in the text of the conclusions.

**Table 1**: Delete the Table file and include it in the manuscript file as a cell-based editable object.

**Response**: Table 1 is removed from the files and left only in the document.

**Graphic Summary, and Figures 5, 6, 7, 8, 9 and 10:** Confirm your authorship of the figures or provide written permission from the photographer to publish the photo(s) under our CC BY 4.0 license.

**Response**: The photographs contained in the graphic summary were taken directly by the lead author Katerine Caviedes-Triana. The graphic summary was designed in the image editing program

PhotoScape V3.7 <http://www.photoscape.org/ps/main/screenshot.php?lc=es>.

While the photographs of the genital structures of the sand flies in figures 5, 6, 7, 8, 9 and 10 were taken by the lead author, Katerine Caviedes-Triana, using the Leica_ICC50W microscope and the names were edited in the Leica LAS EZ program <https://www.leica-microsystems.com/search/#q=Leica%20ICC50%20W>.

No predesigned images or icons from the free software programs used were used. These programs were used exclusively to edit the scientific names of the images and to organize and design the composition from images, silhouettes and icons of their own creation.

**Figures Graphical Abstract, 11B, 12B, 13, and 15:** Confirm whether you drew the images within the figures.

**Response:** All silhouettes were designed freehand by the main author of this document Katerine Caviedes-Triana, using the free program MediBang Paint <https://medibangpaint.com/en/requirement/>.

**Figure 1:** Provide information on permissions associated with the use of map layers.

**Response**: The map was designed in Qgis software, and the georeferenced information used in this study was downloaded from the portal of the National Administrative Department of Statistics - DANE and the Open Data Portal of SIAT-AC - SINCHI Institute, which are licensed under the Creative Commons Attribution 4.0 International License (CC BY 4.0).

Link of conditions of use of the layers: Layers downloaded from the DANE: [Geoportal DANE - Acerca del Geoportal Licencia y condiciones de uso](https://geoportal.dane.gov.co/acerca-del-geoportal/licencia-y-condiciones-de-uso/#gsc.tab=0), Layer downloaded from the SIAT-AC - Instituto SINCHI: <https://datos.siatac.co/pages/coberturas>.

Direct link to the list of layers where the base layer is located “Versión MGN2024-Nivel Departamento” <https://geoportal.dane.gov.co/servicios/descarga-y-metadatos/datos-geoestadisticos/>.

Direct link to the list of layers where you can find the layer of municipalities “Versión MGN2024-Nivel Municipio” <https://geoportal.dane.gov.co/servicios/descarga-y-metadatos/datos-geoestadisticos/>

Direct link to the list of layers where the urban sector layer is located. “Versión MGN2024-Nivel Sector urbano” <https://geoportal.dane.gov.co/servicios/descarga-y-metadatos/datos-geoestadisticos/>.

Direct link to download the layer of the Colombian Amazon boundary layer “Límite de la Amazonia colombiana, Escala: 1:100.000” <https://datos.siatac.co/datasets/7fbe5e6357354c7c9baf8a44369ceef7/about>.

**Data Availability**: Data Availability Statement: Provide on the online submission form the DOI/access number

for each dataset or a direct link to access each dataset.

**Response**: The data that support the findings of this study were deposited in the GenBank database with under the accession numbers: Sequence COI marker: PQ783849, PQ783850, PQ783851, PQ783852, PQ783853, PQ783854, PQ783855, PQ783856, PQ783857, PQ783858, PQ783859, PQ783860, PQ783861, PQ783862, PQ783863, PQ783864, PQ783865, PQ783866, PQ783867, PQ783868, PQ783869, PQ783870, PQ783871, PQ783872, PQ783873, PQ783874, PQ783875, PQ783876, PQ783877, PQ783878, PQ783879, PQ783880, PQ783881, PQ783882, PQ783883, PQ783884, PQ783885, PQ783886, PQ783887, PQ783888, PQ783889, PQ783890, PQ783891, PQ783892, PQ783893, PQ783894, PQ783895, PQ783896, PQ783897, PQ783898, PQ783899, PQ783900, PQ783901, PQ783902.

Sequence Cytb marker: PQ963791, PQ963792, PQ963793

Sequence 12S marker: PQ895496, PQ895497, PQ895498, PQ895499, PQ895500.

**Funding**: Specify the meaning of the initials, to be as specific as possible.

**Response**: The modification is made, resulting in the following text: “The modification is made, resulting in the following text: “This study received financial support from project Hermes 57545 of the Universidad Nacional de Colombia, "Alianza estratégica interdisciplinaria Leticia, Medellín y La Paz para el estudio del microbioma de insectos vectores de enfermedades tropicales y su relación con el cambio climático y la sociedad", Convocatoria Nacional para el Fomento de Alianzas Estratégicas interdisciplinarias que articulen los procesos misionales de la Universidad Nacional de Colombia 2022–2024, awarded to CXMH and Scholarship Program of Ministerio de Ciencia, Tecnología e Innovación, Call 15, for Human Capital Development in the context of the Bicentennial and the 2021–2022 Biennial Plan, awarded to KCT. The funders had no role in study design, data collection and analysis, decision to publish, or preparation of the manuscript”.

**Competing Interests:** Specify whether the authors have conflicts of interest.

**Response:** The authors have declared that no competing interests exist.
